# Potential use of engineered nanoparticles in ocean fertilization for large-scale atmospheric carbon dioxide removal

**DOI:** 10.1038/s41565-022-01226-w

**Published:** 2022-11-28

**Authors:** Peyman Babakhani, Tanapon Phenrat, Mohammed Baalousha, Kullapa Soratana, Caroline L. Peacock, Benjamin S. Twining, Michael F. Hochella

**Affiliations:** 1 Earth Surface Science Institute, School of Earth and Environment, University of Leeds, Leeds, UK; 2 Research Unit for Integrated Natural Resources Remediation and Reclamation (IN3R), Department of Civil Engineering, Faculty of Engineering, Naresuan University, Phitsanulok, Thailand; 3 Center of Excellence for Sustainability of Health, Environment and Industry (SHE&I), Faculty of Engineering, Naresuan University, Phitsanulok, Thailand; 4 Center for Environmental Nanoscience and Risk, Department of Environmental Health Sciences, Arnold School of Public Health, University of South Carolina, Columbia, SC USA; 5 Faculty of Logistics and Digital Supply Chain, Naresuan University, Phitsanulok, Thailand; 6 Bigelow Laboratory for Ocean Sciences, East Boothbay, ME USA; 7 Earth Systems Science Division, Energy and Environment Directorate, Pacific Northwest National Laboratory, Richland, WA USA; 8 Department of Geosciences, Virginia Tech, Blacksburg, VA USA

**Keywords:** Nanoparticles, Environmental, health and safety issues

## Abstract

Artificial ocean fertilization (AOF) aims to safely stimulate phytoplankton growth in the ocean and enhance carbon sequestration. AOF carbon sequestration efficiency appears lower than natural ocean fertilization processes due mainly to the low bioavailability of added nutrients, along with low export rates of AOF-produced biomass to the deep ocean. Here we explore the potential application of engineered nanoparticles (ENPs) to overcome these issues. Data from 123 studies show that some ENPs may enhance phytoplankton growth at concentrations below those likely to be toxic in marine ecosystems. ENPs may also increase bloom lifetime, boost phytoplankton aggregation and carbon export, and address secondary limiting factors in AOF. Life-cycle assessment and cost analyses suggest that net CO_2_ capture is possible for iron, SiO_2_ and Al_2_O_3_ ENPs with costs of 2–5 times that of conventional AOF, whereas boosting AOF efficiency by ENPs should substantially enhance net CO_2_ capture and reduce these costs. Therefore, ENP-based AOF can be an important component of the mitigation strategy to limit global warming.

## Abstract (web summary)

This Analysis considers the potential benefits and challenges of applying engineered nanoparticles for artificial-ocean-fertilization-driven carbon sequestration

## Main

**Fig. 1:**
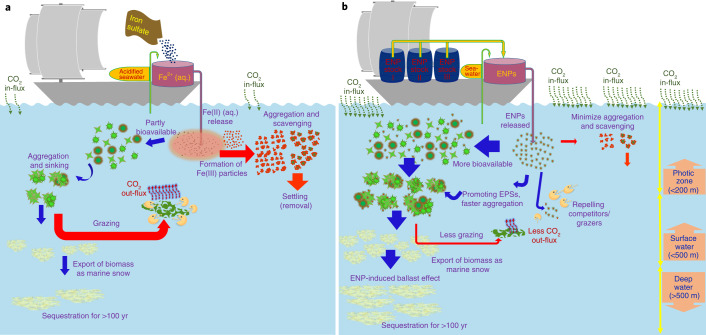
Existing and proposed conceptual models of AOF. Schematics of ocean fertilization based on the conventional approach using iron sulfate (**a**) and the new approach with ENPs as proposed in this study (**b**). In **a**, after dissolving iron sulfate in acidified seawater onboard the ship, the solution is released into the ocean where the soluble iron (Fe(II) (aq.)) may partly be utilized by phytoplankton and cause a bloom, drawing down CO_2_ from the atmosphere. The particulate matter that remains from phytoplankton blooms may then undergo aggregation and subsequent sedimentation. If the biomass resulting from this bloom reaches the deep sea (for example, >1000 m), it may sequester carbon for centuries. However, this whole process might be hindered at the stage when the Fe(II) (aq.) is released into the seawater by the formation of particulate iron (oxyhydr)oxides, which can then aggregate and settle out of the photic zone rather than being bioavailable to phytoplankton. The process might also be hindered by low production rates and/or low settling rate of biomass compared with grazing and remineralization rates. In **b**, the stage of the rapid removal of added nutrients may be minimized by engineering the surface of ENPs, for example, using polymer or bimetallic coating. This may cause added nanoparticle nutrients to become more bioavailable to phytoplankton over a longer period and potentially result in a greater bloom than in the conventional scenario. ENPs may also induce EPS release from phytoplankton cells which can increase phytoplankton aggregation and sedimentation, promote the ballast effect, and thus result in a more effective export of their biomass into the deep ocean.

Recent international climate remediation scenarios rely on massive carbon dioxide removal (CDR) from the atmosphere, in addition to sharp CO_2_ emission reduction, to keep global warming at less than 2 °C (refs. ^1,2^). One CDR approach, artificial ocean fertilization (AOF), involves the intentional addition of a limiting nutrient (typically iron) to stimulate phytoplankton growth and CO_2_ uptake in the oceans (Fig. 1a)^2–9^. A fraction of the stimulated phytoplankton biomass subsequently sinks, exporting carbon to the deep ocean and potentially the ocean floor, for hundreds to thousands of years.

To date, 13 field-scale experiments employing artificial addition of Fe^2+^ into the ocean over 25–300 km^2^ have been conducted, in addition to several field-scale studies monitoring natural occurrences of ocean fertilization by iron^4,6,10^. These studies have shown that although considerable phytoplankton blooms can be stimulated in most artificial additions, their efficiencies in CO_2_ drawdown are much less than those observed for naturally occurring fertilization, for example, via terrestrial dust deposition through the atmosphere^6^. Slow export rates of biomass, especially if not enhanced compared to remineralization rates (the rates at which organic carbon is converted into CO_2_ by bacteria), undo the potential benefits of AOF. Remineralization can also lead to oxygen depletion in subsurface waters and the production of methane and nitrous oxide with higher global warming potential than CO_2_ (ref. ^11^). Nevertheless, with increasingly wide public concerns about the impacts of climate change and the need for negative emission technologies to meet the targets of the 2015 Paris Agreement, recently there has been a renewed interest in AOF^1–5^.

Due to their relatively high number density, and very high specific surface area and diffusivity, nanoparticles (1–100 nm) are probably bioavailable to phytoplankton in the ocean^12–15^. The important role of naturally occurring nanoparticles is already established in natural ocean fertilization^12–14,16–19^. For instance, natural iron (oxyhydr)oxide nanoparticles have been proposed as a source of bioavailable iron in fluxes from glaciers^14,15^, continental sediments^13^, volcanic ashes^12^ and hydrothermal vent emissions^19^. Natural nanoparticles may also supply other nutrients such as phosphorus^16^. These findings suggest that the use of engineered nanoparticles (ENPs) in AOF may lead to desirable efficiencies because of their nanocharacteristics, which resemble those of their natural counterparts. In addition, it is possible to design ENPs to mitigate the drawbacks of the current AOF approach, thereby maximizing their efficiency. If considerable CO_2_ drawdown is achieved by using ENPs, this may allow applications of the approach as a CDR technology at smaller scales or specific locations, and thus allay some of the concerns regarding risks of geoengineering the entire marine ecosystem and downstream ‘nutrient stealing’.

ENP applications in environmental systems at relatively large scales have been widely studied in the past two decades, such as nanoscale zero-valent iron (NZVI) slurries used for soil/groundwater remediation^20–22^ and the application of various ENPs for enhancing sustainable agriculture^23^ and aquaculture^24,25^. In soil/groundwater remediation, despite initial challenges regarding ENP delivery to contaminant source zones and concerns about ENP risks to drinking water resources, the technology has already been commercialized widely around the world^20^.

Here we summarize possible opportunities for ENPs to address the drawbacks and risks of AOF. We further estimate the environmental impacts and economic costs of ENP use for AOF by conducting life-cycle assessment (LCA; Supplementary Information, section 1) and life-cycle costing (LCC; Supplementary Information, section 2) and evaluate the toxic and beneficial aspects of ENPs to marine ecosystems based on data compiled from 123 studies selected among 265 peer-reviewed studies.

## Ocean fertilization challenges and nanotechnology solutions

### Bioavailability and phytoplankton growth enhancement

Metallic nanoparticles may be more bioavailable to phytoplankton than soluble forms due to higher local concentrations generated around phytoplankton cells^26^. This is because of the association of ENPs with planktonic cell surfaces, which may lead to a higher surface concentration of ENPs and their dissolution products and thus more bio-uptake^26^. Iron added in soluble form in previous AOF experiments immediately formed colloidal iron (oxyhydr)oxides and eventually micrometre-sized particles or aggregates that probably sank out of the photic zone, reducing iron bioavailability (Fig. 1a)^4,14,17,18,27–29^. ENPs, due to their surface modification, will address this problem (Fig. 1b), using polymers (for example, carboxymethyl cellulose) and/or metals (for example, aluminium hydroxide) to control their aggregation and dissolution rates, especially within the critical size range of 10–100 nm, thus tuning their functionality and longevity in environmental media^20,21,30^.

**Fig. 2:**
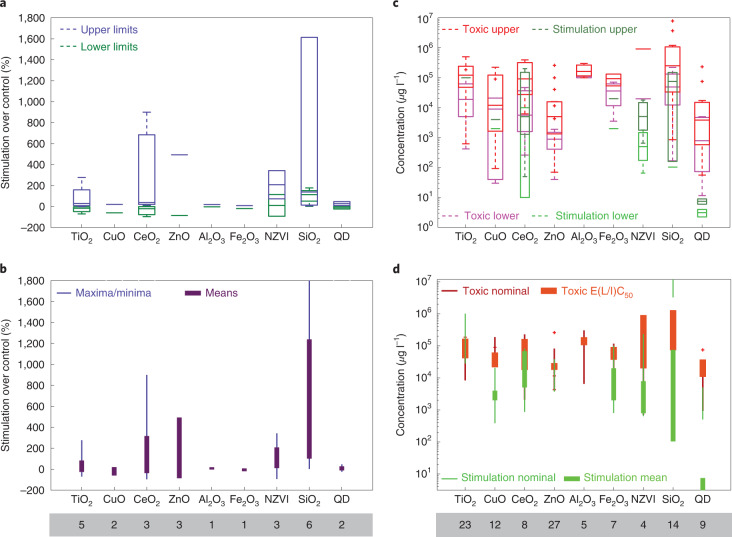
The distinction between stimulating and toxic concentration ranges. **a**, Box plot of lower and upper limits of growth stimulation compared to the control observed for different ENPs. QD, quantum dot. **b**, Minimum and maximum values of the same data (thin bars) compared to the mean values (thick bars). **c**, Concentration ranges of different ENPs causing toxic impacts (acute or chronic toxicity, including median effective (EC_50_), lethal (LC_50_) or inhibitory (IC_50_) concentrations) on different species related to marine ecosystems such as phytoplankton, zooplankton, fish and crustaceans (magenta and red) compared to concentration ranges resulting in promotion of algae/phytoplankton growth (light and dark green). **d**, Nominal ranges of mean concentrations investigated for toxicity in different studies (thin orange bars), and the ranges of mean toxic concentrations (EC_50_, LC_50_ or IC_50_, thick orange bars) observed for different ENPs compared to the nominal ranges of mean concentrations investigated in algae growth stimulation studies (thin green bars) with mean concentration ranges resulting in growth stimulation (thick green bars). The numbers beneath the name of each ENP indicate the number of studies involved in the data ranges. Growth stimulation data are related to marine algal species (21% of data), freshwater species (33% of data) and species common between the two environments (46% of data). The detailed dataset and the list of references are available in Supplementary Tables 1 and 6. On each box of the box plots, the middle mark shows the median, and the bottom and top sides indicate the 25th and 75th percentiles, respectively. The ‘+’ markers indicate individual outliers, and the whiskers show the extent of non-outlier data.

Numerous studies have demonstrated the stimulating effects of ENPs on algal growth (Fig. 2a,b and Supplementary Table 1). Notable algae population enhancement may be achieved using NZVI, SiO_2_ and CeO_2_ ENPs with a mean stimulation effect (cell abundance or growth) increased by 35–756% compared with controls^31–34^. In particular, the role of NZVI in enhancing marine microalgae growth was comparable to or higher than commonly used EDTA-Fe at equimolar concentrations of 11.7 µM (ref. ^31^). This was attributed to similar or higher bioavailability of ENPs, which can bind to extracellular polymeric substances (EPSs) around the algae cell, leading to ENP uptake via endocytosis^31^. Importantly, algal growth rates were similar for a wide range of NZVI concentrations (1.17–117 µM)^31^, revealing another advantage of using ENPs: algal growth is less dependent on ENP concentration in contrast to the substantial impact of the concentration when soluble iron is used^35,36^.

On the question as to whether the concentration of added ENPs should be comparable with natural colloid concentrations for ENPs to be bioavailable, we note that in general it is the interactions and thus affinity between species in solution (whether ionic or nanoparticle species) and surfaces (here phytoplankton EPSs) which control the species bioavailability, not the concentrations^26,31^. Our estimate of the potential ENP concentration range for AOF (as described in the Supplementary Information, section 5) is ~10^10^–10^14^ particles per litre, which is similar to or greater than reported background colloid and nanoparticle concentrations in seawater of ~10^7^–10^12^ particles per litre^37,38^. This ENP number range is also ~800–10^10^ times the potential number concentration of phytoplankton being produced (4 × 10^2^–4 × 10^8^ particles per litre; Supplementary Information, section 5), suggesting that added ENPs are not overshadowed by ambient colloids or by potential phytoplankton numbers created during AOF.

### Co-limitation by other nutrients

**Fig. 3:**
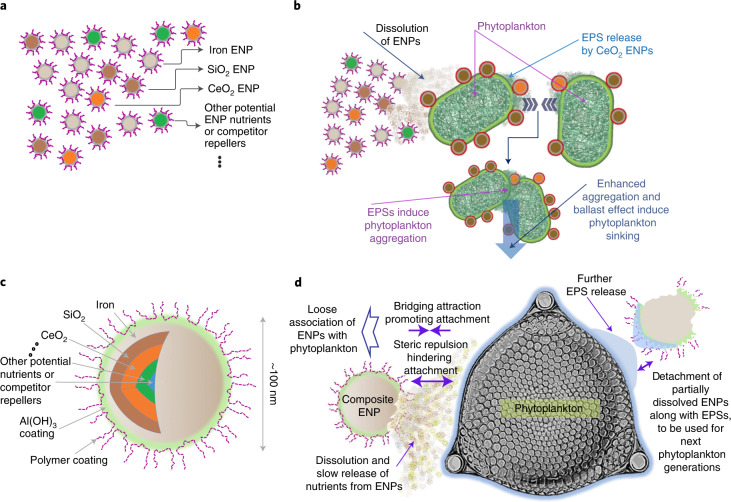
Two possible scenarios for the use of ENPs in AOF. Two possible scenarios in which ENPs can be used in AOF include applying a mixture of ENPs with different coatings (**a**,**b**) and the use of a versatile nanocomposite (**c**,**d**). In general, in response to ENPs, phytoplankton cells produce EPSs which may promote steric repulsion against attachment^34^, while polymer bridging attraction and potentially hydrophobic attractions^63^ may together lead to a loose adherence of ENPs onto phytoplankton surface EPSs^69^. The latter is favourable for AOF because it can lead to ENP detachment from the cell surface after partial utilization, which may provide availability for the next generations of phytoplankton. Detachment of ENP–EPS complex from phytoplankton cells may also result from further excretion of EPSs by phytoplankton in the presence of ENPs^47,64^. In the mixed ENP scenario (**a**,**b**), nanoparticles such as iron ENPs may supply nutrients for phytoplankton via dissolution while promoting EPS generation, which may aggregate phytoplankton cells and increase sinking velocity. Some ENPs such as SiO_2_, which can be designed to interact with cells, may attach to the cell surface EPS and further enhance their sinking rate through the ballast effect (**b**). In the second scenario (**c**,**d**), multilayer core–shell ENPs are designed with thicknesses of different layers in accordance with the amount and the time of nutrients required by phytoplankton species at the time when a nutrient becomes limiting (**d**). All particles may be coated with polymers and/or with metallic layers such as aluminium hydroxide (Al(OH)_3_)^30^ to minimize ENP–ENP attachment via creating steric repulsion or screening attractive magnetic forces. These may also control their interactions with cells as well as their dissolution.

Incomplete bloom development in several AOF experiments was partly attributed to the depletion of secondary nutrients, particularly co-limitation by silicic acid for diatom growth^4,6,39^. Such depletion of secondary limiting nutrients may be addressed effectively within the context of nanotechnology by using a mixture of ENPs or a versatile core–shell nanocomposite as illustrated in Fig. 3. The enhancement in microalgae cell growth in the presence of silica ENPs has already been demonstrated (Fig. 2a,b). Composite nanoparticles containing all nutrients required by phytoplankton can be designed and manufactured in an efficiently engineered architecture for delivery to phytoplankton (Fig. 3c,d) to minimize the total amount of nutrient mass required for a large-scale AOF scheme, thus lowering some of its risks and side effects. Such utilization of nanocomposites currently prevails in other contexts of nanotechnology such as environmental remediation, agricultural/aquaculture enhancement and microalgae biorefinery^21,24,25,40^. Phytoplankton nutrient co-limitation by trace metal micronutrients such as zinc, cobalt, manganese and aluminium has been reported^41,42^, while stimulations of algal growth in the presence of ZnO and Al_2_O_3_ ENPs have also been observed, suggesting that ENPs are promising in addressing co-limitation by other nutrients (Fig. 2a,b).

### Light limitation

Light limitation has been proposed as an important factor in reducing the efficacy of AOF^4^. Within the agritechnology realm, applying ENPs such as TiO_2_ and ZnO to foliage has shown an increased solar light absorption and generally enhanced photosynthetic efficiency^23^. The use of CeO_2_ ENPs, which are biocompatible with a broad range of microorganisms, has shown promise in filtering harmful ultraviolet light and allowing the absorption of useful visible light, thereby protecting microalgae^43,44^. Although this might not directly address limitations by the overall spectrum of the light in ocean settings such as the Southern Ocean, ENPs like CeO_2_ nanoparticles may still improve light-use efficiency by phytoplankton. In general, because ENPs are smaller in size than the wavelength of visible light, they tend to have lower shading effects than larger particulate nutrients such as Fe(III) (oxyhydr)oxides which are naturally formed in conventional AOF. Moreover, light insufficiency hinders photoreduction of colloidal Fe(III) to soluble Fe(II) and thus decreases iron bioavailability for phytoplankton uptake^29^. This may, in turn, accelerate iron removal in particulate form from the water column. On the other hand, potentially well-dispersed ENPs are generally directly bioavailable to phytoplankton without needing further dissolution under light limiting conditions^26,31,33^.

### Export efficiency to deep sea

Application of ENPs to AOF may also enhance the export efficiency of accumulated phytoplankton biomass. Individual phytoplankton cells, especially non-diatom species and live cells, can have a density lower than that of seawater, reducing their sinking potential^45^. Phytoplankton sinking rates can be enhanced when materials with a higher density are adsorbed onto cells or incorporated into aggregates to induce a ballast effect. Silica ENPs (at a concentration of 75 mg l^−1^) were found effective in facilitating the sedimentation and removal of cyanobacteria from the water column^46^. Uptake of silica increased cyanobacteria density and sinking velocity through the formation of cyanobacteria–SiO_2_ complexes. Even a 1% increase in the density of phytoplankton can double the sinking speed^45^. This means the mass ratio of added SiO_2_ ENPs to phytoplankton biomass generated during the bloom could be just 1:100 to double the biomass export rate via the ballast effect.

Another important factor affecting phytoplankton export is their aggregation tendency. Marine phytoplankton EPSs are critical for the formation of marine snow, which is deemed key to phytoplankton export to the deep sea^47^. The use of SiO_2_ and CeO_2_ ENPs has also been proposed to facilitate oil-spill removal by promoting the production of EPSs by marine phytoplankton, leading to enhanced aggregation and sinking^47^. Accordingly, SiO_2_ ENPs might not only address co-limitation by multiple nutrients but also may facilitate the export of the phytoplankton as a ballast agent and phytoplankton EPS promoter. More efficient export of ballasted organic matter may reduce subsurface deoxygenation and methane and nitrous oxide production caused by water column remineralization.

### Longevity of biomass production

Crustacean grazers can shorten phytoplankton bloom lifetime and reduce AOF efficiency^39,48,49^. There are opportunities for ENPs to protect a developing bloom by repelling grazers and competitors, and this may be controlled by tuning the doses of various ENPs considered for AOF. Mesocosm experiments have shown that ENPs such as CuNPs and AuNPs, when added in combination with nutrients (nitrogen and phosphorus), can enhance the bloom continuation by more than 50 days^50^, due to the adverse effect of ENPs on planktonic species competitors. Further, aluminium oxide ENPs skewed the selective feeding pattern of *Daphnia* towards algal feed that was not exposed to ENPs^51^.

Multilayer core–shell ENPs can be designed with a sequence of layers according to the sequence of the micronutrient limitations and subsequent grazing pressure during different stages of phytoplankton bloom development and sinking (Fig. 3c,d). This protects the inner layers of multilayer core–shell ENP against dissolution and therefore dilution in seawater, sustaining the biomass production in surface waters for longer at a lower overall mass of nutrient added. The influence of dilution on the concentration of ENPs is less than that of their soluble form because the diffusivity of ENPs within the size range of 10–100 nm (15.5 down to 1.55 cm^2^ yr^−1^ at 25 °C) is generally one to two orders of magnitude less than that of soluble Fe^2+^ (222.8 cm^2^ yr^−1^ at 25 °C) under quiescent conditions^52^.

Although factors such as biomass sinking, phytoplankton bloom longevity and aggregate size might be countervailing and are currently the subject of ongoing research^7,8,53^, ENPs’ abilities to enhance phytoplankton growth and promote EPS production (which enhances cell aggregation and biomass export) are all advantageous for carbon dioxide removal. Further research is also required to understand these concomitant factors under natural conditions and in the presence of ENPs.

Overall, although future research may test and develop more functionalized hybrid ENPs specifically made for AOF schemes and investigate their fate and ecological risks in the ocean, among the currently used ENPs there are various candidates, including NZVI, iron oxide, SiO_2_, ZnO, CeO_2_, Al_2_O_3_ and TiO_2_ nanoparticles, that may be relevant for future AOF applications (Table 1).

**Table 1:** Summary of the potential role of different ENPs in AOF and their possible drawbacks

ENP	Potential mechanisms of action in AOF	Possible drawbacks
NZVI and iron oxide	Primary limiting nutrient, bioavailability, capability for engineering colloidal stability and chemical durability with surface modification, biocompatible, potential co-benefit for fish growth enhancement	Aggregation and transformation if not engineered properly; high CO_2_-equivalent emissions when used as nanocomposites
SiO_2_	Secondary limiting nutrient, ballast effect for enhanced biomass export to the deep ocean, ENP stability enhancement when used in nanocomposites, promoting the production of EPSs from phytoplankton and enhancing their aggregation and sedimentation, biocompatible, potential co-benefit for fisheries enhancement	Needs high input concentrations if used for ballast effect
CeO_2_	Addressing light limitation, facilitating other nutrient uptake, promoting the production of EPSs from phytoplankton and enhancing their aggregation and sedimentation, protecting against reactive oxygen species	Toxic at high concentrations, low dissolution and relatively unknown fate in the marine environment; unlike other main ENP candidates, its element is not already abundant in the ocean
Al_2_O_3_	Facilitating other nutrient uptake including iron and phosphorous, enhancing carbon export, coating for stabilizing nanocomposites against aggregation and protecting their cores against corrosion, repelling against grazers	Toxic at high concentrations
ZnO	Secondary limiting nutrient, addressing light limitation, coating for controlled dissolution in a nanocomposite, inhibiting grazers, potential co-benefit for fish growth enhancement	Toxic at high concentrations; might generate harmful biodegrading free radicals as a result of photoreactivity
TiO_2_	Addressing light limitation, facilitating other nutrient uptake	Might generate harmful biodegrading free radicals as a result of their photoreactivity; elemental titanium not already abundant in the ocean
Copper (oxide)	Coating for controlled dissolution in a nanocomposite, imposing stress on planktonic species competitors, potential co-benefit for fisheries enhancement	Toxic at high concentrations
QD	Energy donor to algae light-harvesting protein	Toxic at high concentrations; its elements are not already abundant in the ocean
HAp	Source of phosphorus, slow-release of nitrogen when used in nanocomposites	Toxicity (destruction of the mucilage cell wall and extrusion of intracellular substances)

QD, quantum dot; HAp, hydroxyapatite.

## LCA and LCC

**Fig. 4:**
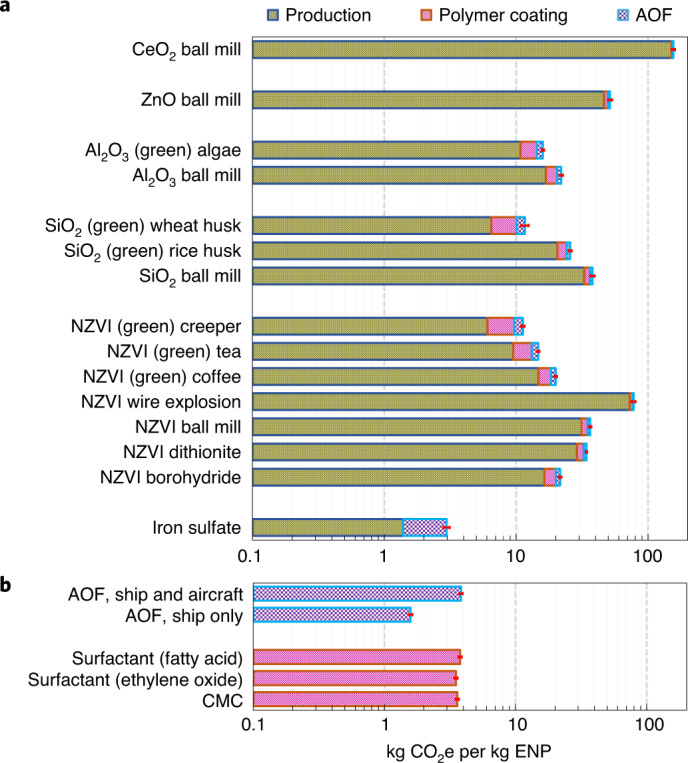
LCA results. CO_2_e emissions determined from LCA for the production of NZVI, SiO_2_, Al_2_O_3_, ZnO and CeO_2_ ENPs by several synthesis methods (**a**), their polymer coatings and their use in AOF (**b**). Iron sulfate previously used in AOF has also been included in the analyses for comparison. AOF processes include transportation from the manufacturing site to the distribution region, distribution over the ocean via either ship or aircraft, and monitoring the AOF performance using similar ships to those used for the distribution. Error bars are uncertainties based on 99% confidence intervals of estimates obtained from Monte Carlo analysis^70^. ENP synthesis methods include sodium borohydride reduction, dithionite reduction, green syntheses using green tea, coffee and Virginia creeper (*Parthenocissus tricuspidata*) leave extracts, ball milling and electrical wire explosion for NZVI; green syntheses and ball milling for SiO_2_; green synthesis and ball milling for Al_2_O_3_; and ball milling for ZnO and CeO_2_. CMC, carboxymethyl cellulose.

**Fig. 5:**
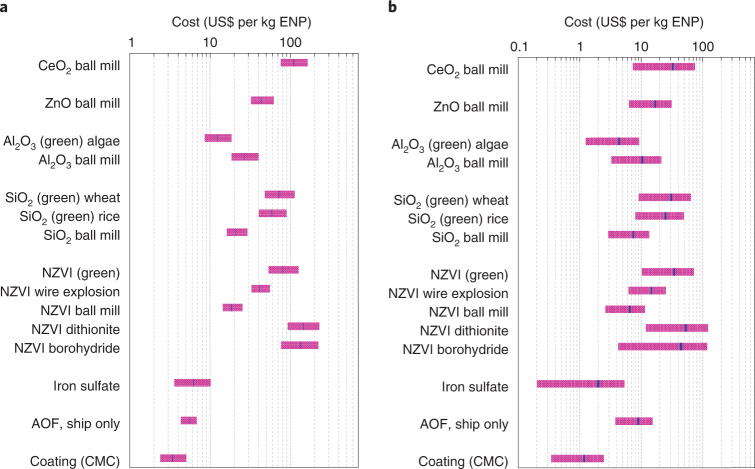
LCC results. Costs after (**a**) and before (**b**) including the labour costs, capital costs and environmental costs for the production of NZVI, SiO_2_, Al_2_O_3_, ZnO and CeO_2_ ENPs by various synthesis methods, using ENP polymer coatings and AOF operation processes (delivery and distribution, using ship only). The analysis has also been conducted for iron sulfate for comparison. The purple bars show the ranges of costs estimated, and the blue lines near the middle of the bars indicate the averaged results.

We conducted an LCA and an LCC analysis for several relevant ENPs (NZVI, SiO_2_, Al_2_O_3_, ZnO and CeO_2_), their polymer coatings and AOF operation (transportation of materials, distribution over the ocean and monitoring of the subsequent impacts). We considered a range of ENP synthesis methods including chemical, mechanical and green (plant extract-based^54^) approaches (Figs. 4 and 5, Supplementary Fig. 1, and Supplementary Tables 2–5). CO_2_-equivalent (CO_2_e) emissions per kg ENPs for all processes involved are 38.3 kg on average (ranging from 11.2 to 156 kg) which is 13 times (or 4–53 times) larger than those of iron sulfate conventionally used in AOF (Fig. 4a,b). The total cost for these processes is on average US$72 per kg ENPs (US$21–153; Fig. 5a). These figures will be smaller if we only consider the best synthesis methods for NZVI, SiO_2_ and Al_2_O_3_ for which several synthesis methods are possible (for example, green methods with 12.9 kg CO_2_e emissions and a cost of US$63.6 per kg ENPs for use in AOF, on average). On average, these lead to 4–10 times larger CO_2_ emissions compared with conventionally used iron sulfate. Henceforth, we discuss these ENP types only, as these may also be the main ENPs for AOF.

While estimating carbon export is difficult and uncertain, especially over long-term periods^53,55^, based on the previous field-scale AOF studies, each kilogram of added iron can lead to ~80–800 kg of CO_2_ removal during the course of a fertilization event^56^ (values converted from molar ratio given in the reference to mass ratio), although 50–67% of this may be compensated by CO_2_ release from excess microbial respiration, decreasing the estimate to 40–400 kg of CO_2_ (refs. ^56,57^). The lower limit of this range is still ~3-fold higher than the amount of CO_2_e emissions per kg NZVI, SiO_2_ and Al_2_O_3_ ENPs synthesized using green methods (12.9 kg), and still higher when synthesized with the ball-milling method (30.0 kg). Based on this efficiency range, our analyses show that AOF with iron sulfate costs ~US$0.030–0.300 per kg CO_2_ removed, which is within previously reported ranges of US$0.008–0.450 (refs. ^9,58^) and leads to 2.97 kg CO_2_e emissions per kg iron used as determined here. Although the use of ENPs (NZVI, SiO_2_ and Al_2_O_3_) may increase costs by ~2–5 times (US$0.16–1.6 with green synthesis methods or US$0.07–0.65 with the ball-milling method for NZVI and SiO_2_) compared with iron sulfate, we have shown here that ENPs may enhance AOF efficiencies much above those of iron sulfate^2^. Further, our LCC analysis also considers labour, capital and environmental costs in addition to monitoring costs which were rarely included previously^58^. Our approach is relatively conservative because the labour, capital and environmental costs are mostly embedded in the prices of materials and processes considered in life-cycle inventories while we have also specified these costs separately (Fig. 5a,b). If we drop these additional costs from our analysis, the use of the aforementioned ENPs increases costs over conventional AOF only by around 1.4–2.2 times (US$0.036–0.37 with all green synthesis methods or US$0.024–0.24 with the ball-milling method for NZVI and SiO_2_; Fig. 5b).

Other potential environmental impacts resulting from our LCA analysis are summarized in Supplementary Table 4, showing that in most cases CeO_2_ synthesis (using the ball-milling method) causes the largest environmental impacts among different ENP types/synthesis methods. Although green methods in all impact categories, except urban land occupation, are the most environmentally friendly synthesis methods, in a few categories (agricultural land occupation, marine eutrophication and urban land occupation) they can also cause large impacts (Supplementary Table 4). The potential economic costs resulting from four important environmental impact categories, that is, climate change, freshwater eutrophication, ozone depletion and terrestrial acidification, are presented in Supplementary Table 3. These costs range from negligible to US$21 (US$1.14 on average) per kg ENP used and are generally highest for climate change (CO_2_e emissions) among different categories, except for green synthesis of NZVI and AOF processes (ship only) which show the highest environmental impact in the category of terrestrial acidification (US$2.4 and US$0.37, respectively; Supplementary Table 3). Further discussions on the feasibility of the ENP use in AOF are presented in Supplementary Information, section 3–5.

## Toxicity of ENPs in oceans

While natural nanoparticles exist in most ocean settings^14,18^, the potential adverse environmental risks of adding ENPs to the ocean require rigorous assessment. We collated toxicity data for ENPs from 98 studies (Supplementary Table 6 and Fig. 2c,d). The ranges of ENP concentrations found to be toxic to benthic and planktonic marine species are compared with concentrations found to stimulate algae growth. These data show that ENP concentrations inducing growth stimulation are mostly below the toxic ranges, especially for NZVI and quantum dots with almost no overlap in the enhanced growth versus toxic ranges, and for SiO_2_, Fe_2_O_3_ and CeO_2_ with slight overlaps (Fig. 2c,d). The concentration range that gives rise to growth stimulation averaged for NZVI, iron oxides, SiO_2_ and CeO_2_ is 2.07–44.3 mg l^−1^ whereas the toxic concentration range averaged for these ENPs is 132–1,090 mg l^−1^. If the upper threshold of the stimulation concentration range is selected for ENP application, this concentration—which is already well below the lower toxic limit—will drop substantially in the ocean by the time ENPs reach deeper habitats due to dilution, and uptake by phytoplankton bloom and other processes. This may partly alleviate concerns about the potential risks of ENPs to marine ecosystems, although potential accumulations from repeated AOF should also be considered. Further, a concentration of 75 mg l^−1^ SiO_2_ ENPs which may induce a ballast effect^46^ is near the lower limit of the SiO_2_ ENP toxic concentration range, 73–1,300 mg l^−1^, suggesting that achieving some levels of ballast effect using ENPs is possible from a toxicology viewpoint.

While the presence of ENPs might induce reactive oxygen species (ROS) production, the promoting effect of several ENPs on algae growth (Fig. 2a,b) suggests that ROS do not induce toxicity within the ENP concentration ranges given above. This is in line with the recent paradigm shift from regarding ROS as creating toxicity to them being essential for biological and physiochemical functions of organisms and impacting nutrient cycling in marine environments^59,60^. Additionally, some ENPs such as CeO_2_ may protect against ROS by scavenging them^32,33^.

## Fate and transport of ENPs in oceans

Although the potentially low concentrations of ENPs required for AOF are favourable for minimizing ENP aggregation, ENP surface modifications are still required to reduce aggregation and settling and thereby lengthen residence time in surface waters^14,20,52,61^. Typically this is achieved using polymers that are inexpensive, efficient, biocompatible, and can be adsorbed onto ENP surfaces via simple physisorption processes and/or using metals that are also aimed to improve ENP performance in a core–shell structure^20,21,30,52^. Polymers generally provide steric repulsion at the surface of ENPs while a metallic shell may reduce the Hamaker constant or enhance electrostatic repulsion to overcome forces such as van der Waals and magnetic attractions^20,21,30,52^. Ligands with low molecular weight (for example, diol ether), which are well solvated in a concentrated brine, have shown a considerable steric stabilization effect in seawater conditions for 10 nm SiO_2_ ENPs^61^. Some polymers (for example, sulfonated copolymers), which do not bind with divalent cations, provide a steric stabilization effect for iron oxide ENPs against bridging flocculation^62^. Such polymers might also reduce the heteroaggregation of ENPs with ubiquitous colloids in seawater, although further research is needed to investigate these effects. Heteroaggregation between ENPs and phytoplankton cells due to polymer bridging^63^ could be one possible pathway for ENP removal from the ocean surface. However, continuous excretion of soluble EPS fraction from phytoplankton^64^, especially due to the presence of ENPs^47^, might cause detachment of ENPs from the cell wall (Fig. 3), and thus reduce ENP removal through heteroaggregation with phytoplankton. Such complex mechanisms are currently poorly understood and warrant further research.

Overall, current studies on the fate and transport of ENPs suggest that nanoparticles are generally unlikely to remain in their original form for long in aquatic environments because various mechanisms of aggregation, dissolution and transformation result in their removal from the water column or alter their characteristics such that they become similar to their natural colloidal counterparts ubiquitous in the environment^17,18^.

## Regulatory and public acceptance challenges

There are already concerns about potential adverse effects of AOF, such as enhancement of methane and nitrous oxide production, impacts on the ocean ecosystem or far-field effects on productivity^4,6,65^. Although operational AOF activities are currently banned under relevant regulatory bodies, for example, the London Convention/London Protocol, the road to conducting small-scale legitimate scientific explorations that meet an environmental assessment framework is still open^4,6,66,67^. There is growing acceptance that more research is needed to evaluate AOF side effects so that the demands of policy-makers and the public for a clearer understanding of such effects can be met^4,6,65^. Further, terrestrial applications of ENPs have already gained favour in several cases such as drinking water decontamination and purification^68^, groundwater remediation^20–22^ and sustainable agriculture/aquaculture^23–25^. Nevertheless, ENP use in AOF is a new realm that also necessitates substantial consideration of potential impacts.

## Conclusions and a roadmap for future research

Although ENPs show promise in addressing many of the current AOF limitations such as bioavailability, nutrient/light co-limitation, phytoplankton bloom longevity and carbon export efficiency, our present information and estimations are based on diverse contexts rather than focused studies on the use of ENPs in realistic AOF conditions. Some potential challenges to overcome include public and regulatory concerns about the potential toxicity of ENPs to marine ecosystems under realistic conditions, unknown long-term impacts of ENP additions on the biogeochemistry of the oceans and the tendency of ENPs to aggregate over time within the marine environment. None of these challenges are necessarily insurmountable.

To begin to address such limitations, research is required in the following key areas:

- Selecting the optimum characteristics of an ideal hybrid ENP or a mixture of several individual ENPs to harvest their combined benefits for the most effective AOF (Fig. 3).
- Designing and manufacturing novel multicomponent/hybrid ENPs to meet the requirements of AOF including delivery of the optimum limiting factors for phytoplankton growth, physicochemical stability in seawater, limited interactions with natural colloids and selective interaction with phytoplankton. Despite the countervailing effects of some of these requirements, nanotechnology may provide a unique opportunity to address them.
- Assessing the ecological impact of using ENPs at all scales, ranging from simple bench-scale laboratory tests to mesocosm and field-scale experiments.
- Further development of ocean biogeochemical models is required to consider a more robust description of AOF-related mechanisms such as ballasting and the burial of biomass in marine sediments^53^. Such models should also consider the fate and transport of ENPs in the ocean and allow for the optimization of ENP performance in AOF. These models may then be used for testing various AOF scenarios on local and global scales, investigating long-term and far-field impacts, and providing tools for decision making.
- Understanding the mechanisms of phytoplankton growth enhancement by ENPs and the fate of ENPs and generated biomass in marine environments that are relevant to realistic AOF conditions.

To achieve substantial CO_2_ removal from the atmosphere via ENP-enabled AOF, long-term repeated additions of ENPs will be needed. Estimating expenses and risks for such an implementation requires determining effective and safe concentration ranges in realistic conditions. This should include considering the fate of added ENPs and exported biomass in deep waters and sediments for understanding the long-term implications of AOF.

Further advancement of the technology may be continued by designing smart ENPs that can target phytoplankton efficiently and enhance phytoplankton aggregation and sinking speed while potentially repelling grazers to protect the biomass export. If part of the ENP mixture or hybrid ENP provides such beneficial activity, this will, in turn, prevent or retard environmental problems such as methane and nitrous oxide production and oxygen consumption.

Overall, ENPs, due to their great number density, specific surface area, diffusion, bioavailability and potential for designing their effective targetability and other functionalities, may provide multiple benefits for application in AOF. These benefits address the challenges of using conventional approaches for AOF (use of dissolved nutrients), which include enhancing bioavailability, nutrient co-delivery, photosynthetic efficiency, export efficiency and phytoplankton bloom longevity. Although our analyses show that CO_2_e emissions and implementation costs for AOF using selected ENPs (NZVI, SiO_2_ and Al_2_O_3_) are higher by 4–10 and 2–5 times, respectively, than using dissolved iron, these results are based on the worst-case scenario where the use of ENPs leads to similar CO_2_ removal as achieved by the conventional approach. The potential for ENPs to address the aforementioned challenges with conventional AOF will likely improve the efficiency of AOF and may alleviate the concerns about its implementation. In conclusion, ENP-based AOF may be a remarkable carbon dioxide removal approach to fight climate change.

## Online content

Any methods, additional references, Nature Portfolio reporting summaries, source data, extended data, supplementary information, acknowledgements, peer review information; details of author contributions and competing interests; and statements of data and code availability are available at 10.1038/s41565-022-01226-w.

## Supplementary information

Supplementary Information Supplementary sections 1–5, Figs. 1–3 and Tables 1–6.

## Data Availability

All data used, including toxicity, growth enhancement, LCA and LCC analysis are presented in the Supplementary Information.
