# Supplementary material for: Potential use of engineered nanoparticles in ocean fertilization for large-scale atmospheric carbon dioxide removal
Source: Nat Nanotechnol. 2022 Nov 28;17(12):1342–51. doi: 10.1038/s41565-022-01226-w (PMC9747614; doi:10.1038/s41565-022-01226-w)
Supplement: Supplementary file 1 — Supplementary sections 1–5, Figs. 1–3 and Tables 1–6. [file 41565_2022_1226_MOESM1_ESM.pdf]

nature nanotechnology

Analysis

https://doi.org/10.1038/s41565-022-01226-w

Potential use of engineered nanoparticles in ocean fertilization for large-scale atmospheric carbon dioxide removal

In the format provided by the authors and unedited

Supplementary Information for
"Potential use of engineered nanoparticles in ocean fertilization for large-scale atmospheric carbon dioxide removal"

Peyman Babakhani[1], *Tanapon Phenrat*[2,3], *Mohammed Baalousha*[4], *Kullapa Soratana*[5], *Caroline L. Peacock*[1], *Benjamin S. Twining*[6], *Michael F. Hochella Jr.*[7,8*]

[1]Earth Surface Science Institute, School of Earth and Environment, University of Leeds, Leeds LS2 9JT, UK

[2]Research Unit for Integrated Natural Resources Remediation and Reclamation (IN3R), Department of Civil Engineering, Faculty of Engineering, Naresuan University, Phitsanulok, Thailand, 65000

[3]Center of Excellence for Sustainability of Health, Environment and Industry (SHE&I), Faculty of Engineering, Naresuan University, Phitsanulok, Thailand, 65000

[4]Center for Environmental Nanoscience and Risk, Department of Environmental Health Sciences, Arnold School of Public Health, University of South Carolina, Columbia 29208, United States

[5]Faculty of Logistics and Digital Supply Chain, Naresuan University, Phitsanulok, Thailand, 65000

[6] Bigelow Laboratory for Ocean Sciences, East Boothbay, ME, USA

[7]Earth Systems Science Division, Energy and Environment Directorate, Pacific Northwest National Laboratory, Richland, WA 99352, USA.

[8]Department of Geosciences, Virginia Tech, Blacksburg, VA 24061, USA.

*Corresponding author. Email: hochella@vt.edu

Text S1. Life cycle assessment

Life cycle assessment (LCA) was conducted for the production process of NZVI, SiO_2, Al_2O_3, ZnO, and CeO_2 ENPs synthesized with different methods, their typical polymer coatings, iron sulfate, and the process of transportation, delivery to the ocean via ship or aircraft (in form of spraying water droplets containing ENPs), and monitoring the subsequent impacts of AOF using ships. We considered NZVI among different iron-based ENPs (e.g., hematite, goethite, and ferrihydrite) because information about various synthesis methods is available for this type of ENP and also because the costs and environmental impacts of synthesizing NZVI may be conservatively generalized to other iron-based ENPs. The analysis was conducted using the SimaPro software (version 9.1), and using the analysis method of ReCiPe 2016 v1.1 Midpoint Method, and Hierarchist version with world normalization (World ReCiPe H) in all assessments after Pati et al.[1] Inventory data were selected mostly based upon Cut-Off, System Process, global market, and allocation at the point of substitution (APOS) unless these were unavailable for a specific material or process in the software databases as shown in Table S2. These inventories were obtained using the EcoInvent database[2] within the SimaPro software. Different ENP synthesis methods include: sodium borohydride or dithionite reduction[3-5], green syntheses[6], ball-milling[7], and electrical wire explosion[8] for NZVI, green synthesis using rice husk[9], wheat husk[10], and ball milling[11] for SiO_2, green synthesis using algae[12] and ball milling[13] for Al_2O_3, and ball milling for ZnO[14] and CeO_2[15]. The functional unit was considered to be 1 kg of ENPs delivered to the ocean. The general system boundary is illustrated in Fig. S2 and includes two major modules: (1) ENP production stage including raw material acquisition, manufacturing processes, and polymer coating of ENPs (1 kg), and (2) AOF related stage consisting of transportation and delivery of ENPs (1 kg) and monitoring the impacts of AOF for 1 kg ENPs added. Potential CO_2 emissions after the addition of ENPs to seawater, such as those from polymer degradation, were not considered in our analyses as while they are currently uninvestigated, they are deemed to be minor. Also, we did not consider the long-term CO_2 emissions resulting from biomass degradation/grazing because that requires substantial in-depth knowledge of the performance of ENPs in real-condition AOF and considering all processes related to ENP-AOF in an ocean circulation model which will be the subject of several future studies. The full list of inventories considered in the present study is provided in Table S2. Uncertainties were calculated based on 99% confidence intervals of estimations after running the analysis 1000 times by random sampling of each input from a normal probability distribution with its mean being equal to the input value. This approach is called Monte Carlo which requires running the LCA for typically 1000 iterations and calculating the average of the final results[16]. The standard deviations that are used in determining the shape of the normal distributions for the input parameters were assumed to be either equal (E) to or half (H) of each input parameter mean value depending on the potential uncertainties that might exist in the estimation of that parameter value. Details of estimating the travel distances for transportation and distribution of the materials in the ocean and subsequent monitoring are presented in the next section.

Text S2. Life cycle costing analysis

Previous cost analyses of AOF[17] have generally relied on CO_2 sequestration outcomes from previous mesoscale experiments which have not generally shown plausible outcomes. Using this approach for new strategies such as ENP application proposed in the present study might underestimate the viability of the approach because an optimized performance of the new strategies for AOF applications is unknown currently. Therefore, here we conduct a life cycle costing (LCC) analysis in which instead of considering CO_2 sequestration level as the functional unit (FU), we consider 1 kg of ENPs delivered to the ocean as the functional unit. In other words, we estimate all costs that may incur during production, transport, delivery, and monitoring the performance of 1 kg of ENPs in the AOF application. Then in a scenario-based approach, we assume the worst-case scenario where the use of ENPs leads at least to efficiencies obtained for iron sulfate conventionally used in AOF. It should be noted that herein the efficiency of AOF is defined as the ratio of the mass of material added to the ocean to the mass of atmospheric CO_2 sequestered upon AOF. In this way, we can estimate the costs for the amount of CO_2 sequestered in this scenario or any other scenario without being dependent on the actual amount of CO_2 sequestration. The system boundary (SB) in our LCC is the same as that considered for LCA (shown in Fig. S2). Costs were estimated assuming the production site of ENPs is located in China and requires delivery to the Southern Ocean as listed in Table S3. Although this might increase the costs of transportation compared to Australia, it may reduce the overall costs due to relatively inexpensive labor/services in China. Unless otherwise specified (in Table S3), we generally get the range of prices/costs for each material or process based on at least five vendors in China.

Text S2.1. Costs for nanoparticle manufacturing and polymer coating

The costs for all processes and materials are mostly estimated based on LCI used in LCA as listed in Table S3. Additional costs (also listed in Table S3) incur due to labor force, capital expenses[18,19], and external (environmental) costs[5] which can be complex to estimate for every industrial-scale ENP manufacturing method based on laboratory synthesis information that are currently available. Thus, we take a simplifying approach based on information available for general manufacturing industries such as microalgae-based biorefineries[18,19].

We include labor costs based on the percentage of labor cost share of the total cost (assumed as the sum of total direct cost, TDC, and labor cost) for the manufacturing industry in general (known as payroll percentages), φ. Then the total cost is estimated by multiplying $100/(100-\varphi)$ by the cost calculated for all other processes and materials considered in LCI (TDC). We assume φ based on production/manufacturing between 12% [20,21] and 66.8% for the UK (conservative value as this can be much less in China, where we base our analysis, due to relatively inexpensive labor costs.[22]

The capital costs are calculated based on National Renewable Energy Laboratory's (NREL's) standard ratios of total direct cost (TDC)[18,19]. These include: warehouse (4%), site development (9%), pro-ratable costs (10%), field construction expenses (10%), home office and construction

(20%), project contingency (10%), maintenance (3%), and other costs (e.g., surveys, piling, soil compaction/dewatering, etc.) (10%). These sum up to 76% of TDC which is added to the production cost of each ENP type. We should mention that in estimating capital costs, TDC does not include the labor cost.

To estimate the external or environmental costs[5], we use the results of our LCA analysis for each ENP synthesis method. The cost related to each factor is calculated based on unit costs given previously[5] (see Table S3) and is then added to the total costs. Here we include four main factors: climate change potential (kg CO_2 equivalent), freshwater eutrophication (kg P equivalent), ozone depletion (kg CFC^{-11} equivalent), and terrestrial acidification (kg SO_2 equivalent), with the unit costs of \$0.14, \$4.6, \$388, and \$9.6, respectively[5]. We should note that wherever a mean value is estimated instead of a range in the above description, the minimum and maximum of ranges in Table S3 have been considered as 10% lower and higher than the mean value.

Text S2.2. Cost for transportation, delivery and monitoring

While all costs are summarized in Table S3, here we provide more details on the costing of AOF-related procedures. The shipment distance from the production site of the materials (assumed China) to the delivery site (assumed the Southern Ocean) is around 5815+6427=12242 km. To estimate the ship travel distance for distributing 1 kg of ENPs over the surface ocean we assume a delivery rate (ship travel distance per kg of ENP to be delivered) similar to conventional AOF using iron sulfate[23] first, and then we assume a scenario in which the required ENP mass concentrations for efficient AOF is lower than those of iron sulfate in conventional AOF. In the IronEx-I experiment[23], 443 kg of iron sulfate was delivered with a ship travelling at the speed of ~8.5 $km.h^{-1}$ over 23.5 h which gives an approximate travel distance of ~200 km, thereby the delivery rate of 0.451 km/kg. Assuming the same ship travel distance is required for monitoring the performance of AOF, and assuming that the delivery is conducted by multiple smaller ships/ferries (compared to a larger ship that carries the materials from the production site to delivery area) at a tonnage of 1000 kg to 5,000 kg ENPs, the travel distance required for delivery of each kg ENPs is around 451 to 2255 km (on average 1353 km) and considering the same distance for monitoring. As a scenario-based investigation, if we expect that the use of ENPs is 5 times more efficient than the use of iron sulfate (since three times higher microalgae growth in presence of ENPs than commonly-used EDTA-Fe at equimolar concentrations has been observed previously[24], and this may be enhanced even further, e.g., to 5 times, by optimizing the system in the future), the delivered concentration should be 5 times lower than that assumed based on conventional AOF. This means that the estimated travel distance by ships distributing each kg of ENPs will be five times higher. Note that this only affects the delivery distance, not the transport distance from the production site to the delivery area. Therefore, the total ship travel distance for transport and distribution of each kg of ENPs is 5×2706+12242=25772 km.

In an alternative scenario, we consider the delivery is performed using aerial spray (agricultural aircraft). Considering the IronEx-I experiment[23] like above, we estimate that the area

covered by each kg of ENPs added to the ocean is ~0.7 km^2. The current costs we have found are £1300 to $3880 per km^2 [25,26], leading to a range of $939 to $2802 per kg ENPs delivered which is much higher than ship distribution costs, suggesting that the use of airplanes is not an economical option, and thus we have not included this in the results shown in Fig. 4.

The freight cost for transporting 1000 kg (e.g., 10 boxes of 100 kg each) of materials from Guangdong, China, to Australia (5815 km) is $269 – $352 (from Ref.[27] enquired in March 2022) which is equivalent to $0.046–$0.061 t.km or 0.053 t.km on average. A more precise quote was obtained from SeaRates European Team[28], for transporting dry goods from Nanjing, China (through Shanghai Port) to Melbourne Port, Australia with a total travel distance of 6,566 km. They gave a price of $5420 including all administration and handling costs for a 20′ container which has a standard payload capacity of 25,000 kg[29]. This leads to a transport cost of 0.033 t.km which is less than the above mean estimation. Considering the total travel distance of 26,000 km, the cost of transport and delivery with the more conservative unit price of 0.053 t.km is $1.4 for 1 kg ENPs.

Other miscellaneous transportation costs include handling in and out ($26.6 per ton), storage ($57.1 per ton), vanning/devanning ($16.6 per ton), and other administrative charges such as CFS charges, Original Documentation fee, re-weighting, ENS Fee Exempt, and Export Customs Clearance Fee ($6.77 per ton)[30], lead to a sum of $107.0 per ton. These estimates are based on container transport prices, i.e., 20′ containers which have a volume of 33.2 m^3 and a payload of 25,000 kg. We assume that 20′ containers are sufficient for ENP powder shipment (compared to 40′ containers) since given the density of ENPs in the range of 1500 – 7870 $kg.m^{-3}$, the total cargo volume (<25000 L) should be smaller than the capacity of 20′ containers. In total, such miscellaneous transportation costs add an approximate sum of $0.107 per kg ENPs to the aforementioned estimates, equivalent to 0.0038 $/(t.km) after dividing by the total travel distance (26000 km). This is considered as the upper limit of the range in Table S3 while the lower limit of the range is obtained from the quote described above[28].

Costs for labor, fuel consumption, and CO_2 emission related to transportation and delivery are already covered in the above estimations (i.e., the prices of materials and services). However, additional labor costs might incur for processing and delivery of ENP and for monitoring of AOF. Processing (e.g., handling, dispersion of powder in water, etc.) and delivery are assumed to be proportional to the rate of ENP release into the ocean from the ship which in the case of the IronEx-I experiment[23] is 443 kg of iron sulfate released over 23.5 h. Considering the five times higher efficiency expectation for ENPs than iron sulfate, this amounts to a five times lower ENP concentration delivered and thus five times lower release rate, 3.77 kg/h, or in other words, each kg of ENP is delivered in 0.265 h. Assuming two persons (one professional and one non-profession) are required for this operation, and considering the hourly labor cost of $2.1 to $3.7 for normal workers and $3.7 to $6.6 for professionals and assuming labors are from China[31], the total average labor cost is $2.73 per kg ENPs delivered.

For monitoring, we assume that an additional professional person is required for sampling and measurement, yielding a total cost of $1.75 per kg ENPs. We should note that this cost may be ignored or reduced for large-scale implementation of AOF since monitoring may be conducted through satellite remote sensing or glider technologies which are more economical[17]. Overall, the total cost for transportation from the manufacturing location, delivery to the ocean and post-processing (monitoring) is $4.797 for each kg of ENPs.

Text S3. Further discussion on life cycle analysis and life cycle costing and comparison with the literature

Some of our current LCA assessments are based on the laboratory-scale production of ENPs, which can be less environmentally friendly than industrial-scale production, e.g., the instrument for electrical wire explosion synthesis of NZVI or other ENPs has so far been built only at a laboratory scale and there is limited information about its energy consumption and efficiency. Also, several green synthesis approaches use filter papers in the laboratory which contributes to a significant portion of CO_2 emissions (Fig. S1), whereas other more-environmentally friendly methods such as centrifugation may be used at an industrial-scale production[32]. Furthermore, energy consumption for processes such as grinding, filtration, and centrifugation at an industrial scale is within ranges from 0.001 to 0.016 $kwh.kg^{-1}$ [32], whilst the ranges we have used in the LCA are at least an order of magnitude larger, e.g., 0.070 to 0.697 $kwh.kg^{-1}$ for different methods of NZVI synthesis. Likewise, estimations based on laboratory ball milling instruments do not show that this approach is the most environmental-friendly for any of the ENPs investigated although it could become more environmental-friendly[33]. The ball milling method for NZVI and SiO_2 ENPs has the lowest cost among different synthesis methods (Fig. 4c). In conducting LCA for ZnO and CeO_2 production, suitable information was only available for the ball milling method which showed high CO_2-equivalent emissions of 50.0 and 154.3 kg CO_2-equivalent, respectively which could have potentially been less if other production methods were considered.

Although according to the previous studies[1,34], green methods for the synthesis of ENPs might not necessarily lead to a reduction in CO_2 emission impacts over conventional chemical approaches, in our study, it turns out that recent advances in green synthesis methodologies[6,10,12] may warrant a substantial reduction in environmental impacts of ENP production (Fig. 4a).

CO_2 emission estimations from previous studies[35-38] are summarized in Fig. S3, for several relevant ENPs (magnetite, ZnO/zinc, Al_2O_3, TiO_2, and copper). Excluding copper, CO_2-equivalent emissions associated with other ENP syntheses range from 0.5 to 52 kg (16.6 kg on average) per kg ENPs which is similar to or lower than the range we have estimated for the synthesis of relevant ENPs (NZVI, SiO_2, Al_2O_3, ZnO, and CeO_2) that is from 6 to 150 kg (33 kg on average) CO_2-equivalent per kg ENPs. This demonstrates that our LCA approach has been relatively conservative and thus environmental impact assessment at more practical scales may be less than what we have presented here.

Furthermore, to compare our LCC results with the current market prices, we searched for the prices of ENPs from various current manufacturers of ENPs even though almost all of these manufacturers work at small-scale production of ENPs (i.e., per kg) rather than large-scale (per ton). The results compared with the summary of our LCC analyses are presented in Table S5. Although we have conducted our LCC at an industrial scale, we have generally taken a conservative approach in our estimations including assumptions about the labor and capital cost which form a substantial part of total costs (Table S3)[18,19]. Yet, in general, our estimations of costs are 2 to 7 folds lower than the current market prices (Table S5). In a more detailed investigation that may be conducted in the future, we expect these costs to drop even further[32].

Manufacturing nanocomposites might cause significantly higher CO_2 emissions than single ENPs, although it still highly depends on the synthesis method and the type of coating[35,39]. For instance, CO_2 emissions for magnetite ENP production were reported to rise from 0.1 kg CO_2-equivalent per kg of polymer-coated iron oxide ENPs to 3.7 kg CO_2-equivalent per kg of silica-coated iron oxide ENPs[39]. These require further investigations in the future.

Text S4. Further discussion on the feasibility of creating ballast effect using ENPs in AOF

In order to gain enhancement in export efficiency using SiO_2 ENPs via the ballast effect, we suggested in Section 1.4 that a mass ratio of added SiO_2 ENPs to phytoplankton biomass of 1:100 is required to double the biomass export rate. Conservatively assuming that each kg of biomass contains 1 kg CO_2, to double its export rate, there is a need for ~0.01 kg SiO_2 ENPs (based on an uncertain assumption that all added SiO_2 ENPs sorb onto phytoplankton). The use of such an amount of SiO_2 can countervail ~10% of the CO_2 sequestered as a result of SiO_2 ENP production/delivery (Fig. 4a), suggesting that inducing the ballast effect based on adding SiO_2 ENPs produced using current methods might result in a substantial amount of CO_2-equivalent emissions and thus more environmentally friendly synthesis methods of SiO_2 are needed in the future.

Text S5. Estimation of the number of engineered nanoparticles versus the number of phytoplankton cells and ubiquitous colloids in seawater

Based on the conservative range of AOF efficiencies described in Section 2, i.e., 40 to 400 kg of CO_2 removal per kg of materials added in AOF[40], and expecting that the use of ENPs should at least enhance the upper range of these efficiencies by five times, the mass ratio of CO_2 sequestered to ENPs added could range from 40 and 2000.

Assuming the mass concentrations of ENPs added (here we only consider iron as a representative of all ENP types) are equal to those of soluble iron used in previous AOF experiments as summarized by Strong et al.[41], the implementation concentrations were determined by dividing the mass of iron added in each experiment by the volume of the mixed layer (i.e., the surface area of experiment multiplied by the depth of the mixed layer assumed 62 m [17]). The resulting mass concentrations range from 7×10^{-5} to 1×10^{-3} $mg.L^{-1}$ (3×10^{-4} $mg.L^{-1}$ on average).

Considering the ENP size range, 10–100 nm, and phytoplankton (e.g., microplankton such as diatoms) size range, 20–200 µm [42], and considering the density of iron, 7.87 g.cm^{-3}, and phytoplankton (organic carbon), 1.5 g/cm^3, we estimate number ratio of ENPs added to the ocean and phytoplankton based on the calculating particle volumes with a spherical shape assumption. These estimations with considering different combinations of input parameter ranges results in ENP numbers ranging from 2×10^{10} to 3×10^{14} particles/L (~10^{14} particles/L on average) and phytoplankton numbers ranging from 4×10^{2} to 4×10^{8} (~10^{8} on average) and lead to a particle number ratio (ENPs number to phytoplankton number) of ~800 to 4×10^{10} particles/L (~10^{9} particles/L on average). These suggest that ENPs outnumber phytoplankton cells. If similar efficiencies with conventional AOF are assumed, this number ratio range will be even 5 times larger. Based on the aforementioned range of efficiencies, for removing anthropogenic CO_2 emissions in one year (e.g., 43×10^{9} tons of CO_2 emitted in 2019)[43], ~20–1000 million tons of ENPs are needed that may be possible from an industrial viewpoint, e.g., annual iron ore mine production in Australia alone is 900 million tons based on 2021 data[44]. Obviously, AOF is not the only CDR strategy available[45], and thus assuming the removal of entire anthropogenic CO_2 emissions by AOF is an extreme case scenario.

In comparison to natural colloids in seawater (mentioned in Section 1.1), particle number concentrations of ENPs (~10^{10}–10^{14}) are similar to or greater than reported background colloid/nanoparticle concentrations in seawater, 4×10^{7} to 1.5×10^{12} particles/L [46,47], suggesting that ENPs would not be overshadowed by indigenous colloids.

Figures and Tables

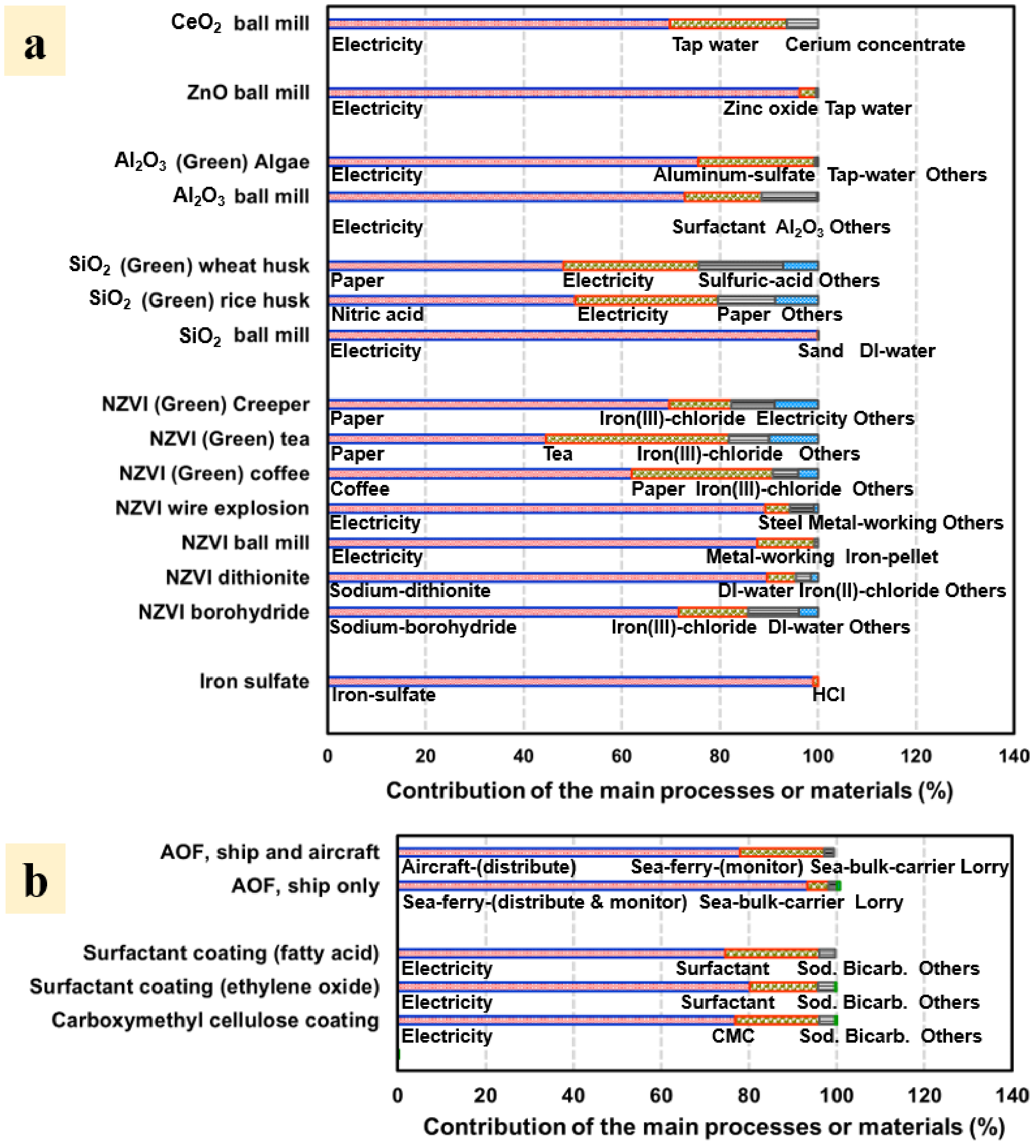

Figure S1. Normalized results for different processes or materials contributing to CO_2-equivalent emissions for the production of each ENP type and iron sulfate (a) and polymer coating for ENPs and AOF processes (transportation/delivery/monitoring) (b). Here, only three

main contributing factors are shown and CO_2-equivalent emissions from the rest of the processes have been summed up and shown as "Others".

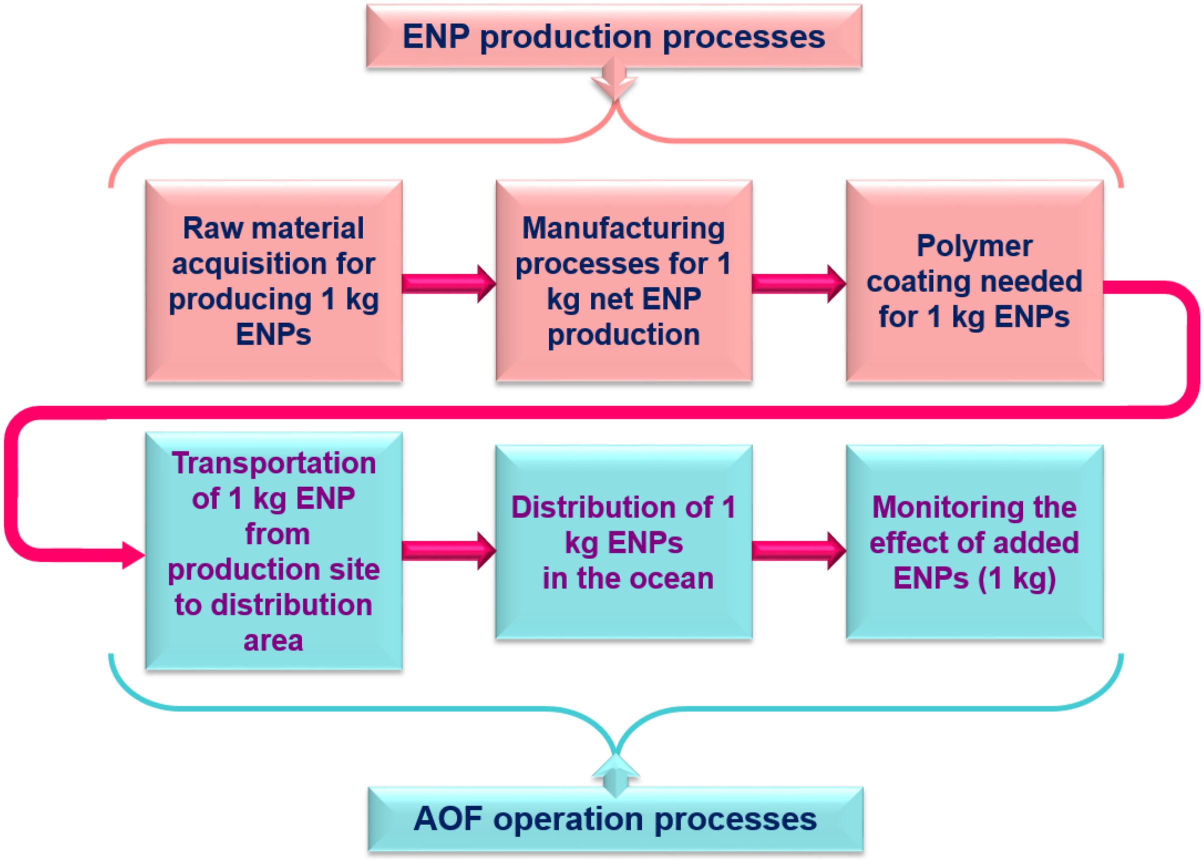

Figure S2. The general system boundary used in the LCA and LCC.

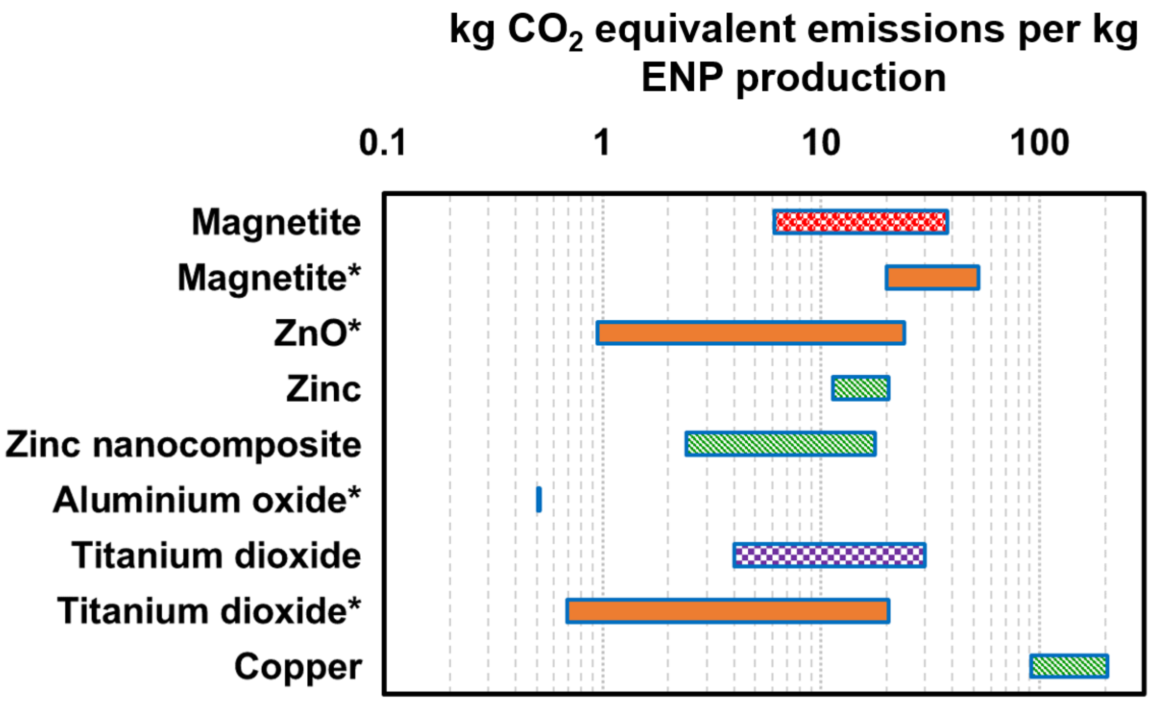

Figure S3. CO_2 equivalent (kg) emitted during ENP production processes for each kg of ENPs from literature. Relevant data were found for magnetite (Fe_3O_4), zinc or zinc oxide (ZnO), zinc nanocomposite with poly propylene (PP) plastic, aluminum oxide (Al_2O_3), titanium dioxide (TiO_2), and copper nanoparticles. Solid, orange-colored bars (titles marked with a star) including the single data-point of aluminum oxide are from Falinski et al.[35], assuming cumulative energy demand (CED) (kWh/kg) times 0.707 is equal to kg CO_2 equivalent emissions following US EPA[48]. Red sphere-filled bar is from Ref.[36], green, hashed bars are from Ref.[37], and violet, the checkered bar is from Ref.[38].

Table S1. Summary of selected studies on phytoplankton/alga growth stimulated by different engineered nanoparticles.

ENP	Coating/media	Organism	Species environment: freshwater (FW) or seawater (SW)	Dose range investigated ($\mu g.L^{-1}$)	Dose creating growth stimulation ($\mu g.L^{-1}$)	The amount of stimulation (cell abundance or biomass increase) compared to control (%)*	The amount of boosting in lipid, chlorophyll a content, or fatty acid, compared to control (%)*	Ref.
NZVI	Na-acrylic copolyme	green algae Desmodesmus subspicatus, Dunaliella salina, Parachlorella kessleri and Raphidocelis subcapitata; the eustigmatophycean algae Nannochloropsis limnetica and Trachydiscus minutus ; cyanobacterium Arthrospira	FW and SW	51 – 510000	500 – 5100	-93 – 73.4	22.8 – 180	[49]
NZVI	Na-acrylic copolymer or inorganic coating	Pavlova lutheri, Isochrysis galbana and Tetraselmis suecica	SW and FW	65.3 – 6533.9	65.3 -653	114 – 343	-13.4 – 41.9	[50]
NZVI	Sulfide-modified seeded with silica	Chlamydomonas reinhardtii	FW	1800 – 180000	1800 – 18000			[51]
α-Fe_2O_3		Scenedesmus obliquus	FW	800 – 100000	2000 – 20000	-18.7 – 8.1	18.1 – 39.6	[52]
Fe_3O_4**	cationic surfactant-decorated	Chlorella sp. KR-1	FW				127	[53]
Fe_3O_4**		Nannochloropsis maritima	SW	20000 – 120000	120000			[54]
TiO_2		Chlorella vulgaris	FW and SW	100000 – 5000000	100000	-26.7 – 16.3	11.3	[55]

ENP	Coating/media	Organism	Species environment: freshwater (FW) or seawater (SW)	Dose range investigated (µg.L^{-1})	Dose creating growth stimulation (µg.L^{-1})	The amount of stimulation (cell abundance or biomass increase) compared to control (%)*	The amount of boosting in lipid, chlorophyll a content, or fatty acid, compared to control (%)*	Ref.
TiO_2		diatom Phaeodactylum tricornutum	SW	2500 – 40000			17.5 – 57.6	[56]
TiO_2		Chlorophyta Trebouxiophyceae	FW	10000		-71 – 277	-19 – 27	[57]
TiO_2		Synechocystis sp.	FW and SW	25		0	11.5 – 62.2	[58]
TiO_2	humic acid	microalgae Pseudokirchneriella subcapitata	FW	100 – 10000		-6.5 – 41		[59]
SiO_2	CH3	Chlorella vulgaris	FW and SW	2000000		177 – 210	340 – 610	[60]
SiO_2		C. vulgaris	FW and SW			6.9		[61]
SiC			FW and SW	50000 – 250000	150000	2 – 16	1.7 – 18.9	[62]
SiO_2		Diatoms Chaetoceros sp., Skeletonema sp. and Thalassiosira sp.	SW	3000000 – 6000000		126.7 – 5822	7992 – 85227	[63]
SiO_2		green algae Porphyridium aerugineum Geitler	FW	1000 – 1000000	100000 – 500000	100.4 – 138.5	103.9 – 160.4	[64]
cobalt ferrite/silica	methyl functionalized	Chlorella vulgaris	FW and SW	10000000 – 50000000	50000000		-68.2 – 198	[65]
Ag		Cyanobacterium Calothrix elenkinii	FW and SW	100 – 500	200		-40.3 – 104	[66]
Ag		diatom alassiosira sp.	FW and SW	5 – 200	100		11.6 – 17.4	[67]
Ag		D. salina and Chlorella Thalassiosira	FW and SW	10 – 5000	10 – 100	-88.1 – 37.5		[68]
CeO_2		diatom Phaeodactylum tricornutum	SW	2500 – 40000	5000	6 – 14	17.6 – 37.9	[56]

ENP	Coating/media	Organism	Species environment: freshwater (FW) or seawater (SW)	Dose range investigated (μg.L^{-1})	Dose creating growth stimulation (μg.L^{-1})	The amount of stimulation (cell abundance or biomass increase) compared to control (%)*	The amount of boosting in lipid, chlorophyll a content, or fatty acid, compared to control (%)*	Ref.
CeO_2		marine diatom (Phaeodactylum tricornutum), marine chlorophyte (Nannochloris atomus) and freshwater chlorophyte (Chlamydomonas reinhardtii)	FW and SW	100 – 200000	10000 – 200000	-96.3 – 900	-38.9 – 264	[69]
CeO_2		D. salina and Chlorella autotrophica	FW and SW	10 – 5000	10 – 50	-20.8 – 37.7		[70]
Cu	carboxylated with citric acid	Chlorella vulgaris	FW and SW	670 – 40000	2000 – 4000	-60 – 20		[71]
CuO		Cyanobacterium Calothrix elenkinii	FW	100 – 1000			-70.1 – 181	[66]
ZnO		Alexandrium minutum	SW	100 – 100000				[72]
ZnO		Chlorophyta Trebouxiophyceae	FW	10000 – 10000		-86 – 493	-13 – 42	[57]
ZnO		Cyanobacterium Calothrix elenkinii	FW	1000 – 5000			-70.4 – 241	[66]
Al2O3		Chlorella sp.	FW	1000000		-3 – 18.9		[73]
CdSe QD		diatom Phaeodactylum tricornutum	SW	2.25 – 54.0	2.25 – 8.99	-24.4 – 9.1		[74]
CdSe/ZnS QD		green alga Dunaliella tertiolecta	SW	0.10 – 0.48	0.10 – 0.38	8 – 20		[75]
CdSe QD	thioglycerol coating	red Algae Porphyridium cruentum	FW and SW	1000 – 10000	4 – 6	2 – 47		[76]

ENP	Coating/media	Organism	Species environment: freshwater (FW) or seawater (SW)	Dose range investigated ($\mu g.L^{-1}$)	Dose creating growth stimulation ($\mu g.L^{-1}$)	The amount of stimulation (cell abundance or biomass increase) compared to control (%)*	The amount of boosting in lipid, chlorophyll a content, or fatty acid, compared to control (%)*	Ref.
NZVI	Na-acrylic copolyme	green algae Desmodesmus subspicatus, Dunaliella salina, Parachlorella kessleri and Raphidocelis subcapitata; the eustigmatophycean algae Nannochloropsis limnetica and Trachydiscus minutus ; cyanobacterium Arthrospira	FW and SW	51 – 510000	500 – 5100	-93 – 73.4	22.8 – 180	[49]
NZVI	Na-acrylic copolymer or inorganic coating	Pavlova lutheri, Isochrysis galbana and Tetraselmis suecica	SW and FW	65.3 – 6533.9	65.3 -653	114 – 343	-13.4 – 41.9	[50]
NZVI	Sulfide-modified seeded with silica	Chlamydomonas reinhardtii	FW	1800 – 180000	1800 – 18000			[51]
α-Fe_2O_3		Scenedesmus obliquus	FW	800 – 100000	2000 – 20000	-18.7 – 8.1	18.1 – 39.6	[52]
Fe_3O_4**	cationic surfactant-decorated	Chlorella sp. KR-1	FW				127	[53]
Fe_3O_4**		Nannochloropsis maritima	SW	20000 – 120000	120000			[54]
TiO_2		Chlorella vulgaris	FW and SW	100000 – 5000000	100000	-26.7 – 16.3	11.3	[55]
TiO_2		diatom Phaeodactylum tricornutum	SW	2500 – 40000			17.5 – 57.6	[56]

ENP	Coating/media	Organism	Species environment: freshwater (FW) or seawater (SW)	Dose range investigated (μg.L^{-1})	Dose creating growth stimulation (μg.L^{-1})	The amount of stimulation (cell abundance or biomass increase) compared to control (%)*	The amount of boosting in lipid, chlorophyll a content, or fatty acid, compared to control (%)*	Ref.
TiO_2		Chlorophyta Trebouxiophyceae	FW	10000		-71 – 277	-19 – 27	57
TiO_2		Synechocystis sp.	FW and SW	25		0	11.5 – 62.2	58
TiO_2	humic acid	microalgae Pseudokirchneriella subcapitata	FW	100 – 10000		-6.5 – 41		59
SiO_2	CH3	Chlorella vulgaris	FW and SW	2000000		177 – 210	340 – 610	60
SiO_2		C. vulgaris	FW and SW			6.9		61
SiC			FW and SW	50000 – 250000	150000	2 – 16	1.7 – 18.9	62
SiO_2		Diatoms Chaetoceros sp., Skeletonema sp. and Thalassiosira sp.	SW	3000000 – 6000000		126.7 – 5822	7992 – 85227	63
SiO_2		green algae Porphyridium aerugineum Geitler	FW	1000 – 1000000	100000 – 500000	100.4 – 138.5	103.9 – 160.4	64
cobalt ferrite/silica	methyl functionalized	Chlorella vulgaris	FW and SW	10000000 – 50000000	50000000		-68.2 – 198	65
Ag		Cyanobacterium Calothrix elenkinii	FW and SW	100 – 500	200		-40.3 – 104	66
Ag		diatom alassiosira sp.	FW and SW	5 – 200	100		11.6 – 17.4	67
Ag		D. salina and Chlorella Thalassiosira	FW and SW	10 – 5000	10 – 100	-88.1 – 37.5		68
CeO_2		diatom Phaeodactylum tricornutum	SW	2500 – 40000	5000	6 – 14	17.6 – 37.9	56
CeO_2		marine diatom (Phaeodactylum	FW and SW	100 – 200000	10000 – 200000	-96.3 – 900	-38.9 – 264	69

ENP	Coating/media	Organism	Species environment: freshwater (FW) or seawater (SW)	Dose range investigated (µg.L[-1])	Dose creating growth stimulation (µg.L[-1])	The amount of stimulation (cell abundance or biomass increase) compared to control (%)*	The amount of boosting in lipid, chlorophyll a content, or fatty acid, compared to control (%)*	Ref.
		tricornutum), marine chlorophyte (Nannochloris atomus) and freshwater chlorophyte (Chlamydomonas reinhardtii)						
CeO_2		D. salina and Chlorella autotrophica	FW and SW	10 – 5000	10 – 50	-20.8 – 37.7		70
Cu	carboxylated with citric acid	Chlorella vulgaris	FW and SW	670 – 40000	2000 – 4000	-60 – 20		71
CuO		Cyanobacterium Calothrix elenkinii	FW	100 – 1000			-70.1 – 181	66
ZnO		Alexandrium minutum	SW	100 – 100000				72
ZnO		Chlorophyta Trebouxiophyceae	FW	10000 – 10000		-86 – 493	-13 – 42	57
ZnO		Cyanobacterium Calothrix elenkinii	FW	1000 – 5000			-70.4 – 241	66
Al_2O_3		Chlorella sp.	FW	1000000		-3 – 18.9		73
CdSe QD		diatom Phaeodactylum tricornutum	SW	2.25 – 54.0	2.25 – 8.99	-24.4 – 9.1		74
CdSe/ZnS QD		green alga Dunaliella tertiolecta	SW	0.10 – 0.48	0.10 – 0.38	8 – 20		75
CdSe QD	thioglycerol coating	red Algae Porphyridium cruentum	FW and SW	1000 – 10000	4 – 6	2 – 47		76

* In these columns the lowest and highest ENP stimulation impacts observed in each study are listed. The lower limits which are negative indicate that adverse effects of ENPs have been observed in those studies along with stimulation effect, noting the maximum values of ranges are all positive.

**In the analysis, this study's result is excluded as outliers.

Table S2. Detailed consideration of inventories in the life cycle assessment (LCA) for different ENPs synthesized with different methods, polymer coating conventional iron sulfate, and transportation/delivery/monitoring process of artificial ocean fertilization. The functional unit is considered as 1 kg of ENP produced, transported, delivered, and monitored.

ENP/ synthesis methods/ AOF process	Materials/ processes	Estimation/ assumption description	Amount	Unit	Uncertainty range*	Consideration in SimaPro	Ref.
**NZVI Electrical wire explosion**							[8]
	Steel	Assumed the same volume as ball milling method	1.80	kg	H	Steel, unalloyed [GLO]| market for | APOS, S	[5]
	Wire explosion electricity	Estimated using $W = 0.5\ C.V^2$ (where W is energy (J), C is the capacitance (F) and V is the voltage).	91.8	kwh	E	Electricity, medium voltage [GLO]| market group for | APOS, S	
	Wire drawing	Converting steel to wire	1.8	kg	E	Wire drawing, steel [RoW]| processing | APOS, S	
	Metalworking	Further potential work on the steel	1.8	kg	E	Metalworking, average for steel product manufacturing [GLO]| market for | APOS, S	
**Ball milling**							[5]

ENP/ synthesis methods/ AOF process	Materials/ processes	Estimation/ assumption description	Amount	Unit	Uncertainty range*	Consideration in SimaPro	Ref.
	Iron pellet	Conservatively assumed the same yield of 55% used in the ball milling method following the Refs.	1.80	kg	H	Iron pellet [GLO]| market for | APOS, S	1,5
	Ball milling electricity	20.8 kWh for 1 kg steel following the Ref.	37.4	kWh	E	Electricity, medium voltage [GLO]| market group for | APOS, S	7
	Metalworking	Further potential work on iron pallet	1.80	kg	H	Metalworking, average for steel product manufacturing [GLO]| market for | APOS, S	
**Dithionite reduction**							3
	$FeCl_2$ $4H_2O$	Calculated from stoichiometry reaction assuming a theoretical yield of reaction, 100%	3.56	kg	H	Iron(II) chloride [GLO]| market for | Cut-off, S	
	$Na_2S_2O_4$	Calculated from stoichiometry reaction assuming a theoretical yield of reaction, 100%	6.69	kg	H	Sodium dithionite, anhydrous [RoW]| market for sodium dithionite, anhydrous | Cut-off, S	
	NaOH	Added to adjust the pH at 10	0.0016	kg	H	Sodium hydroxide, without water, in 50% solution state [GLO]| market for | Cut-off, S	
	HCl	For washing	0.0026	kg	H	Hydrochloric acid, without water, in 30% solution state [RoW]| market for | Conseq, S	
	Milli-Q water	For washing; Assumed following the ref.	200	kg	E	De-ionized water, reverse osmosis, production mix, at the plant, from surface water RER S System - Copied from ELCD	5
	N_2 purging	Assuming for 5 min at a rate of 25 mL/s	0.77	kg	H	Nitrogen, via cryogenic air separation, production mix, at plant,	

ENP/ synthesis methods/ AOF process	Materials/ processes	Estimation/ assumption description	Amount	Unit	Uncertainty range*	Consideration in SimaPro	Ref.
						gaseous EU-27 S System - Copied from ELCD	
	Magnetic stirring	Assumed following the ref.	0.07	kWh	E	Electricity, medium voltage [GLO]| market group for | APOS, S	[5]
	Storing under N_2 environment	Assuming 1.5 times the amount of N_2 needed as above in case of wet storage	1.15	kg	E	Venting of nitrogen, liquid [GLO]| market for | Cut-off, S	
**Borohydride reduction**							[3]
	$FeCl_3\ 6H_2O$	Calculated from stoichiometry reaction assuming a theoretical yield of reaction, 100%	2.90	kg	H	Iron (III) chloride, without water, in 40% solution state [GLO]| market for | Cut-off, S	
	NaOH	Calculated from stoichiometry reaction assuming a theoretical yield of reaction, 100%	0.44	kg	H	Sodium hydroxide, without water, in 50% solution state [GLO]| market for | Cut-off, S	
	HCl	For washing and storage	0.0032	kg	H	Hydrochloric acid, without water, in 30% solution state [RoW]| market for | Cut-off, S	
	Milli-Q water	For washing	200	kg	E	De-ionized water, reverse osmosis, production mix, at the plant, from surface water RER S System - Copied from ELCD	
	$NaBH_4$	No available in the databases; A separate inventory was made for this as described below	0.65	kg	H	Sodium Borohydride	
	Magnetic stirring	Assumed following the ref.	0.07	kWh	E	Electricity, medium voltage [GLO]| market group for | APOS, S	[5]

ENP/ synthesis methods/ AOF process	Materials/ processes	Estimation/ assumption description	Amount	Unit	Uncertainty range*	Consideration in SimaPro	Ref.
**Borohydride production process (for 1 kg)**							[4]
	Borax ($Na_2B_4O_7$)		1.37	kg	H	Borax, anhydrous, powder [GLO]| market for | Cut-off, S	
	Sodium (metallic)		2.51	kg	H	Sodium [GLO]| market for | Cut-off, S	
	H_2		0.11	kg	E	Hydrogen, gaseous [GLO]| market for hydrogen, gaseous | Cut-off, S	
	SiO_2 (quartz)		2.87	kg	H	Silica sand [GLO]| market for | Conseq, S	
	Grinding in an agate mill		0.07	kWh			[5]
	Melting in furnace		7.6	kWh	E	Electricity, medium voltage [GLO]| market group for | APOS, S	[77]
	Pouring on the water	Around 1000 kJ (Energy of heating silica to 1200 °C) /4186 J/kg (Specific Heat of water)	239	kg	E	Process water, ion exchange, production mix, at the plant, from surface water RER S	
	Drying	Assuming an oven with a capacity of 64 L and wattage of 3000 (Temp. range of up to 500 °C) and that it can be used with full volumetric capacity for input materials with equivalent density to water (50%) and an equal amount of water (50%).	1.19	kWh	E	Electricity, medium voltage [GLO]| market group for | APOS, S	[78]
	Grinding	Assuming an agate mill with a capacity of 4.5 L and wattage of 1100 W and that it can be used	0.52	kWh	E	Electricity, medium voltage [GLO]| market group for | APOS, S	[79]

**ENP/ synthesis methods/ AOF process**	**Materials/ processes**	**Estimation/ assumption description**	**Amount**	**Unit**	**Uncertainty range***	**Consideration in SimaPro**	**Ref.**
		with full volumetric capacity for input materials with a density equivalent to 1 g/cm^3, and the grinding period is for 0.5 h.					
	Sieving	Assuming the same energy as mixing, a power of 1/10 hp and a capacity of 50 gal	0.01	kWh	E	Electricity, medium voltage [GLO]| market group for | APOS, S	
	H_2 addition at 22 atm	volume assumed as 1 m^3	1.00	m^3	E	Compressed air, 1200 kPa gauge [RoW]| market for compressed air, 1200 kPa gauge | APOS, S	
	Autoclave		1.60	kWh	E	Electricity, medium voltage [GLO]| market group for | APOS, S	[80]
	Heating	At 450 °C for 70 min	0.74	kWh	E	Heat, district or industrial, natural gas [GLO]| market group for | APOS, S	
	Evaporation under vacuum	1.34	1.34	kWh	E	Electricity, medium voltage [GLO]| market group for | APOS, S	

ENP/ synthesis methods/ AOF process	Materials/ processes	Estimation/ assumption description	Amount	Unit	Uncertainty range*	Consideration in SimaPro	Ref.
**Green synthesis methods**							[6]
**either**	Virginia creeper	Assumed analogous to organic grass which exists in the databases; Conservatively assumed a yield of 55%	1.79	kg	H	Grass, organic [RoW]| grass production, permanent grassland, organic, extensive | APOS, S	[1]
**or**	Coffee		1.79	kg	H	Coffee, green bean [RoW]| coffee green bean production, robusta | APOS, S	
**or**	Green tea		1.79	kg	H	Tea, dried [GLO]| market for tea, dried | APOS, S	
	Pretreatment	Washing with water assuming water consumption 10 times of the washed materials	17.9	kg	E	Tap water [GLO]| market group for | APOS, S	
	Pretreatment	Drying at room temperature considering the process for drying agricultural products in air assuming twice the amount of biomass mass with equivalent volume with water density	3.59	L	E	Dried roughage store, air dried, solar [GLO]| market for | APOS, S	
	100 mL DI water	Assume using tap water at an industrial scale will be appropriate	35.9	kg	E	Tap water [GLO]| market group for | APOS, S	
	Boiling	In 100 mL DI water at 80℃ for 80 min	0.05	kWh	H	Electricity, medium voltage [GLO]| market group for | APOS, S	[78]
	Filter paper	Assuming paper used similar to the Ref.	3.84	kg	E	Kraft paper, unbleached [GLO]| market for | APOS, S	[5]
	Filtration energy	Assuming energy used similar to the Ref.	0.02	kWh	H	Electricity, medium voltage [GLO]| market group for | APOS, S	[5]
	$FeCl_3.6H_2O$	0.1 M in tap water	1.00	kg	H	Iron (III) chloride, without water, in 40% solution state [RoW]| iron (III)	

ENP/ synthesis methods/ AOF process	Materials/ processes	Estimation/ assumption description	Amount	Unit	Uncertainty range*	Consideration in SimaPro	Ref.
						chloride production, product in 40% solution state | Cut-off, S	
	Water		35.9	kg	E	Tap water [GLO]| market group for | APOS, S	
	Stirring for 24 h	Assuming the same energy as mixing	0.68	kWh	H	Electricity, medium voltage [GLO]| market group for | APOS, S	[81]
**SiO_2 Ball milling**							[11]
	Natural sand without any pretreatment	Conservatively assumed a yield of 50%	2.00	kg	H	Sand [RoW]| market for sand | APOS, S	[1]
	DI water		4.00	kg	E	Water, deionized [RoW]| market for water, deionized | APOS, S	
	Vibratory sieving	10 min at an amplitude of 100 mm	0.0037	kWh	E	Electricity, medium voltage [GLO]| market group for | APOS, S	[81]
	Ball milling	8 h, based on a laboratory ball-mill (P4 Fristch) with 450 mL capacity and a power of 2.2 kW	46.6	kWh	E	Electricity, medium voltage [GLO]| market group for | APOS, S	[82]
**Green - rice husk**							[9]
	Rice husk		1.04	kg	H	Rice husk (raw), at processing/CN Economic	
	Water	Assuming tap water for washing	103	kg	H	Tap water [GLO]| market group for | APOS, S	
	Drying agricultural	Assuming twice the amount of biomass mass with equivalent volume with water density;	2.07	kg	E	Operation, dried roughage store, air dried, solar [GLO]| market for | APOS, S	

ENP/ synthesis methods/ AOF process	Materials/ processes	Estimation/ assumption description	Amount	Unit	Uncertainty range*	Consideration in SimaPro	Ref.
	products in the air	Assuming an oven with a capacity of 100 L and wattage of 1000 (Temp. range of 40 to 250 °C) and that it can be used with full volumetric capacity for input materials with equivalent density to water (50%) and an equal amount of water (50%).					
	Drying	At 80 °C for 12 h	0.25	kWh	E	Electricity, medium voltage [GLO]| market group for | APOS, S	
	Burning inside muffle furnace	At 400 °C for 1 h; Assuming a laboratory oven with a capacity of 64 L and wattage of 3000 (Temp. range of up to 500 °C) and that it can be used with full volumetric capacity for input materials with equivalent density to water.	0.10	kWh	E	Electricity, medium voltage [GLO]| market group for | APOS, S	
	Burning inside muffle furnace	At 700 °C for 1 h; Assuming an oven with a capacity of 64 L and wattage of 3000 (Temp. range of up to 500 °C) and that it can be used with full volumetric capacity for input materials with equivalent density to water (50%) and an equal amount of water (50%).	0.10	kWh	E	Electricity, medium voltage [GLO]| market group for | APOS, S	
	Water	To produce 1 M HNO_3 solution	51.8	kg	H	Tap water [GLO]| market group for | APOS, S	
	HNO_3	To produce ash in HNO_3 (1 M)	3.26	kg	E	Nitric acid, without water, in 50% solution state [RoW]| market for nitric acid, without water, in 50% solution state | Cut-off, S	

ENP/ synthesis methods/ AOF process	Materials/ processes	Estimation/ assumption description	Amount	Unit	Uncertainty range*	Consideration in SimaPro	Ref.
	Stirring	For 10 h; Assuming the same energy as mixing	0.41	kWh	E	Electricity, medium voltage [GLO]| market group for | APOS, S	81
	Water	Assuming tap water for washing	103	kg	H	Tap water [GLO]| market group for | APOS, S	
	Ethanol	Assuming the same as the amount of materials being washed	1.04	kg	E	Ethanol, without water, in 99.7% solution state, from ethylene [RoW]| ethylene hydration | APOS, U	
	Filter paper	Assuming paper used similar to the Ref.	2.22	kg	E	Kraft paper, unbleached [GLO]| market for | APOS, S	5
	Filtration energy	Assuming energy used similar to the Ref.	0.01	kWh	E	Electricity, medium voltage [GLO]| market group for | APOS, S	5
	Drying	At 80 °C for 3 h under vacuum; Assuming an oven with a capacity of 100 L and wattage of 1000 (Temp. range of 40 to 250 C) and that it can be used with full volumetric capacity for input materials with equivalent density to water (50%) and an equal amount of water (50%).	0.06	kWh	E	Electricity, medium voltage [GLO]| market group for | APOS, S	
	Ball milling	2 h (averagely), based on a laboratory ball-mill (P4 Fristch) with 450 mL capacity and a power of 2.2 kW	6.02	kWh	E	Electricity, medium voltage [GLO]| market group for | APOS, S	82
**Green - wheat husk**							10
	Wheat husk	Conservatively assumed a yield of 75%	1.33	kg	H	Flax husks [GLO]| market for flax husks | APOS, S	

ENP/ synthesis methods/ AOF process	Materials/ processes	Estimation/ assumption description	Amount	Unit	Uncertainty range*	Consideration in SimaPro	Ref.
	Water for washing	Water (assuming tap water) for washing	133	kg	H	Tap water [GLO]| market group for | APOS, S	
	Drying	Drying agricultural products in air assuming twice the amount of biomass mass with equivalent volume with water density	2.67	kg	H	Operation, dried roughage store, air dried, solar [GLO]| market for | APOS, S	
	Drying at 60 °C for 24 h	Assuming an oven with a capacity of 100 L and wattage of 1000 (Temp. range of 40 to 250 °C) and that it can be used with full volumetric capacity for input materials with equivalent density to water (50%) and an equal amount of water (50%).	0.64	kWh	H	Electricity, medium voltage [GLO]| market group for | APOS, S	
	Water	To produce H_2SO_4 (10%)	40.0	kg	H	Water, deionized [RoW]| market for water, deionized | APOS, S	
	H_2SO_4	at 10%	8.02	kg	H	Sulfuric acid (98% H2SO4), at plant/RER Economic	
	Heating and vigorous stirring at 90 °C for 3 h	Assuming an average hot plate stirrer with 0.55 kW power and capability of 15 L	1.63	kWh	H	Electricity, medium voltage [GLO]| market group for | APOS, S	83
	Filter paper	Assuming paper used similar to the Ref.	2.85	kg	E	Kraft paper, unbleached [GLO]| market for | APOS, S	5
	Filtration energy	Assuming energy used similar to the Ref.	0.01	kWh	H	Electricity, medium voltage [GLO]| market group for | APOS, S	5
	Calcination	Using a muffle furnace at 600 °C for 2 h; Assuming an oven with a capacity of 94 L and wattage of 4500 (Temp. range of up to 600 °C) and that it can be used with	0.26	kWh	H	Electricity, medium voltage [GLO]| market group for | APOS, S	

ENP/ synthesis methods/ AOF process	Materials/ processes	Estimation/ assumption description	Amount	Unit	Uncertainty range*	Consideration in SimaPro	Ref.
		full volumetric capacity for input materials with equivalent density to water (50%) and an equal amount of water (50%).					
	Washing the resulted powder with distilled water		13.3	H	Water, deionized [RoW]| market for water, deionized | APOS, S		
**Al2O3 Ball milling**							[13]
	Aluminum powder	Conservatively assumed a yield of 55%	1.80	kg	H	Aluminum oxide, metallurgical [UN-OCEANIA]| aluminum oxide production | APOS, S	[1]
	Initial grinding	Assumed for transforming metallurgical aluminum oxide in the database to powder form; Assuming an agate mill with a capacity of 4.5 L and wattage of 1100 W and that it can be used with full volumetric capacity for input materials with a density	0.44	kWh	E	Electricity, medium voltage [GLO]| market group for | APOS, S	[84]

ENP/ synthesis methods/ AOF process	Materials/ processes	Estimation/ assumption description	Amount	Unit	Uncertainty range*	Consideration in SimaPro	Ref.
		equivalent to 1 g/cm3, and the grinding period of 0.5 h.					
	Paraffin oil	This can be substituted by surfactant according to the Ref.; Assuming the density of surfactant equivalent to water	0.83	kg	E	Non-ionic surfactant [GLO]| non-ionic surfactant production, ethylene oxide derivate | APOS, S	[85]
	Air addition	Assuming 16 times	0.20	m^3	E	Compressed air, 800 kPa gauge [RoW]| market for compressed air, 800 kPa gauge | APOS, S	
	Ball milling	4 h, based on a laboratory ball-mill (P4 Fristch) with 450 mL capacity and a power of 2.2 kW	16.5	kWh	E	Electricity, medium voltage [GLO]| market group for | APOS, S	[82]
**Green - Algae**							[12]
	Algae		0.07	kg	E	Lime [FR]| production, algae | APOS, S	
	Tap water for washing	1.5 L for 15 g	66.7	kg	E	Tap water [GLO]| market group for | APOS, S	
	DI water for washing	0.15 L for 15 g	6.67	kg	E	Water, deionized [RoW]| market for water, deionized | APOS, S	
	Drying in shade for 1 week	Assuming the analogous to the process of drying agricultural products in the air that is available in the database	0.07	kg	E	Operation, dried roughage store, air dried, solar [GLO]| market for | APOS, S	
	Grinding	Assuming an agate mill with a capacity of 4.5 L and wattage of 1100 W and that it can be used with full volumetric capacity for input materials with an equivalent	0.02	kWh	H	Electricity, medium voltage [GLO]| market group for | APOS, S	[84]

ENP/ synthesis methods/ AOF process	Materials/ processes	Estimation/ assumption description	Amount	Unit	Uncertainty range*	Consideration in SimaPro	Ref.
		density equivalent to 1 g/cm^3, and the grinding period is for 0.5 h.					
	Heating	At 70 °C for 30 min; Assuming an average hot plate stirrer with 0.55 kW power and capability of 15 L	3.67	kWh	H	Electricity, medium voltage [GLO]| market group for | APOS, S	[83]
	Aluminum Sulfate	0.05 M; Assuming 100% theoretical yield of the reaction	3.36	kg	H	Aluminum sulfate, powder [RoW]| production | APOS, U	
	stirring for 1 h	Assuming a power of 1/10 hp and capacity of 50 gal	0.09	kWh	H	Electricity, medium voltage [GLO]| market group for | APOS, S	[81]
	Heating at 70 C for 25 min	Assuming an average hot plate stirrer with 0.55 kW power and capability of 15 L	3.33	kWh	H	Electricity, medium voltage [GLO]| market group for | APOS, S	[83]
	Oven drying at 180 C for 1 h	Assuming an oven with a capacity of 64 L and wattage of 3000 (Temp. range of up to 500 °C) and that it can be used with full volumetric capacity for input materials with equivalent density to water (50%) and an equal amount of water (50%).	0.32	kWh	H	Electricity, medium voltage [GLO]| market group for | APOS, S	[78]
	Calcination at 1200 for 2 h	Assuming an oven with a capacity of 23 L and wattage of 7000 (Temp. range of up to 1200 °C) and that it can be used with full volumetric capacity for input materials with equivalent density to water (50%) and an equal amount of water (50%).	4.09	kWh	H	Electricity, medium voltage [GLO]| market group for | APOS, S	
**ZnO**							

ENP/ synthesis methods/ AOF process	Materials/ processes	Estimation/ assumption description	Amount	Unit	Uncertainty range*	Consideration in SimaPro	Ref.
**Ball milling**							14
	Zinc oxide (powder)	Conservatively assumed a yield of 55%	1.80	kg	H	Zinc oxide [GLO]| market for | APOS, S	1
	Initial grinding	Assumed for transforming metallurgical material available in the database to powder form; Assuming an agate mill with a capacity of 4.5 L and wattage of 1100 W and that it can be used with full volumetric capacity for input materials with a density equivalent to 1 g/cm^3, and the grinding period of 0.5 h.	0.44	kWh	E	Electricity, medium voltage [GLO]| market group for | APOS, S	
	Cooling with chilled water	Assuming the use of a typical water cooler with 8 liters per h production and 0.085 kW input power and water Temp. of 1-10℃ following Ref.[86]	3.06	kWh	E	Electricity, medium voltage [GLO]| market group for | APOS, S	
	Water needed for cooling	Assuming a similar mass of water with the zinc oxide mass is consumed in every hour of milling	288	L	E	Tap water [GLO]| market group for | APOS, S	
	Ball milling	20 h; Based on a laboratory ball-mill (P4 Fristch) with 450 mL capacity and a power of 2.2 kW	58.5	kWh	E	Electricity, medium voltage [GLO]| market group for | APOS, S	82
**CeO_2 Ball milling**							15
	Cerium	Conservatively assumed a yield of 55%	1.80	kg	H	Cerium concentrate, 60% cerium oxide [GLO]| market for | APOS, S	1

ENP/ synthesis methods/ AOF process	Materials/ processes	Estimation/ assumption description	Amount	Unit	Uncertainty range*	Consideration in SimaPro	Ref.
	Grinding	Assumed for transforming metallurgical material available in the database to powder form; Assuming an agate mill with a capacity of 4.5 L and wattage of 1100 W and that it can be used with full volumetric capacity for input materials with a density equivalent to 1 g/cm^3, and the grinding period of 0.5 h.	0.44	kWh	E	Electricity, medium voltage [GLO]| market group for | APOS, S	
	Cooling with chilled water	Assuming the use of a typical water cooler with 8 liters per h production and 0.085 kW input power and water Temp. of 1-10℃ following Ref.[86]	4.59	kWh	E	Electricity, medium voltage [GLO]| market group for | APOS, S	
	Water needed for cooling	Assuming a similar mass of water with the zinc oxide mass is consumed in every hour of milling	432	kg	E	Tap water [GLO]| market group for | APOS, S	
	Ball milling	30 h optimum; Based on a laboratory ball-mill (P4 Fristch) with 450 mL capacity and a power of 2.2 kW	86.6	kWh	E	Electricity, medium voltage [GLO]| market group for | APOS, S	82
	CeO_2 extraction as a Rare Earth Element						87
	Electricity		24.6	kWh	E	Electricity, medium voltage [GLO]| market group for | APOS, S	
	Heat		33.6	kWh	E	Electricity, medium voltage [GLO]| market group for | APOS, S	

ENP/ synthesis methods/ AOF process	Materials/ processes	Estimation/ assumption description	Amount	Unit	Uncertainty range*	Consideration in SimaPro	Ref.
	Water consumption		44910	kg	E	Tap water [GLO]| market group for | APOS, S	
**Polymer (for coating the surface of 1 kg ENP)**							88
**either**	Carboxymethyl cellulose (CMC)	Equilibrium adsorption concentration between 400 to 600 mg/L; ENP (e.g., NZVI) concentration assumed 100 mg/L	0.17	kg	H	Carboxymethyl cellulose, powder [RoW]| production | Cut-off, S	
**or**	surfactant (ethylene oxide derivate)	Equilibrium adsorption concentration between 400 to 600 mg/L; ENP (e.g., NZVI) concentration assumed 100 mg/L	0.17	kg	H	Non-ionic surfactant [GLO]| non-ionic surfactant production, ethylene oxide derivate | Cut-off, S	
**or**	surfactant (fatty acid derivate)	Equilibrium adsorption concentration between 400 to 600 mg/L; ENP (e.g., NZVI) concentration assumed 100 mg/L	0.17	kg	H	Non-ionic surfactant [GLO]| non-ionic surfactant production, fatty acid derivate | Cut-off, S	
	$NaHCO_3$	At a concentration 0.125 mM	0.11	kg	E	Sodium bicarbonate [GLO]| market for sodium bicarbonate | Cut-off, S	
	mixing for 48 h at 30 rpm	Assuming a power of 1/10 hp and capacity of 50 gal	0.02	kWh	E	Electricity, medium voltage [GLO]| market group for | APOS, S	81
	Centrifugation for 80 min at 27500 rpm	Assuming a power of 1.2 kW and a capacity of 400 mL	4.00	kWh	E	Electricity, medium voltage [GLO]| market group for | APOS, S	89
**Transportation,**							

ENP/ synthesis methods/ AOF process	Materials/ processes	Estimation/ assumption description	Amount	Unit	Uncertainty range*	Consideration in SimaPro	Ref.
**delivery, and monitoring**							
	Ship for transport	Distance from nearest port to the production site of the materials (assumed China) to the nearest port in the delivery area (assumed Port of Melbourne). Further description can be found in the Supplementary text of Life cycle costing Section.	12.2	t.km	H	Transport, freight, sea, bulk carrier for dry goods [GLO]| market for transport, freight, sea, bulk carrier for dry goods | APOS, S	
**either**	Smaller ships for distribution	Delivery via smaller ships at a rate of 0.451 km/kg, leading to a travel distance of 902 to 4510 km multiplied by five for expected higher efficiency of using ENPs. Further description can be found in the Supplementary text of Life cycle costing Section.	6.8	t.km	E	Transport, freight, sea, ferry [GLO]| market for transport, freight, sea, ferry | APOS, S	
**or**	Aircraft	Delivery via smaller aircraft at a rate of 0.451 km/kg, leading to a travel distance of 902 to 4510 km multiplied by five for expected higher efficiency of using ENPs. Further description can be found in the Supplementary text of Life cycle costing Section.	6.8	t.km	E	Transport, freight, aircraft, medium haul [GLO]| market for transport, freight, aircraft, medium haul | APOS, S	
	Small ships for monitoring	Assuming the same travel distance as the distribution.	6.8	t.km	E	Transport, freight, sea, ferry [GLO]| market for transport, freight, sea, ferry | APOS, S	

**ENP/ synthesis methods/ AOF process**	**Materials/ processes**	**Estimation/ assumption description**	**Amount**	**Unit**	**Uncertainty range***	**Consideration in SimaPro**	**Ref.**
	Lorry	Land transport over distance from factory to port assumed 300 km	0.30	t.km	E	Transport, freight, lorry >32 metric ton, euro5 [RoW]| market for transport, freight, lorry >32 metric ton, EURO5 | APOS, S	
**Soluble iron**							23
	$FeSO_4.7H_2O$	Iron sulfate	5.0	kg	H	Iron sulfate [RoW]| market for iron sulfate | Cut-off, S	
	HCl	Concentrated acid (assuming 28%) added to keep the iron dissolved upon release into the seawater (0.0011%)	0.014	kg	H	Hydrochloric acid, without water, in 30% solution state [RoW]| market for | Cut-off, S	

* In the Monte Carlo approach used for estimating the uncertainties, the standard deviations that are used in determining the shape of the normal distributions, which are used for sampling the input parameters, were assumed to be either equal (E) to or half (H) of each input parameter value depending on the potential uncertainties that might exist in the estimation of that parameter value.

Table S3. Detailed consideration of inventories in the life cycle costing (LCC) for different ENPs synthesized with different methods, polymer coating conventional iron sulfate, and transportation/delivery/monitoring process of artificial ocean fertilization. The functional unit is considered as 1 kg of ENP produced, transported, delivered, and monitored.

ENP/ synthesis methods/ AOF process	Materials /processes	amount	Unit	Min Unit Cost ($)	Max Unit Cost ($)	Mean Unit Cost ($)	Min Total Cost ($)	Max Total Cost ($)	Mean Total Cost ($)	Note on the unit cost
**NZVI**										
**Electrical wire explosion**										
	Steel	1.80	kg	0.40	1.00	0.70	0.72	1.80	1.3	The range is obtained for industrial grade iron wire from different manufacturers in China. This range is above the price in USA in 2019 for iron ore, $0.112, iron/steel slag, $0.0275, and iron/steel scrap, $0.226-$0.310 per kg as reported[90].
	Wire explosion electricity	91.78	kwh	0.06	0.09	0.08	5.51	8.26	6.9	The min is from Ref.[33] and the max is obtained for household electricity price in China[44].
	Wire drawing	1.8	kg	0	0	0	0	0	0	Included in the price of wire iron material above
	Metal working	1.8	kg	0	0	0	0	0	0	Included in the price of wire iron material above
	Labor cost	8.1	$	12.00	66.80	39.40	9.25	24.53	13.4	Described in the text
	Capital cost	8.1	$	0.68	0.84	0.76	5.57	6.81	6.2	Described in the text
	External - climate change	72.61	kg CO_2 eq	0.13	0.15	0.14	9.15	11.18	10.2	Described in the text
	External - freshwater eutrophication	3.65E-02	kg P eq	4.10	5.02	4.56	0.15	0.18	0.2	Described in the text

ENP/ synthesis methods/ AOF process	Materials /processes	amount	Unit	Min Unit Cost ($)	Max Unit Cost ($)	Mean Unit Cost ($)	Min Total Cost ($)	Max Total Cost ($)	Mean Total Cost ($)	Note on the unit cost
	External - ozone depletion	3.46E-06	kg CFC^{-11} eq	349	427	388	0.00	0.00	0.0	Described in the text
	External - terrestrial acidification	0.28	kg SO_2 eq	8.64	10.56	9.60	2.42	2.95	2.7	Described in the text
**Ball milling:**										
	Iron pellet	1.80	kg	0.20	0.80	0.50	0.36	1.44	0.9	The range is obtained for industrial grade iron powder from different manufacturers in China giving a price range of $0.2 to $0.8 per kg. This range is above the price in USA in 2019 for iron ore, $0.112, iron/steel slag, $0.0275, and iron/steel scrap, $0.226-$0.310 per kg as reported[90].
	Ball milling electricity	37.44	kWh	0.06	0.09	0.08	2.25	3.37	2.8	The min is from Ref.[33] and the max is obtained for household electricity price in China[44].
	Metal working	1.80	kg	0	0	0	0	0	0.0	Included in the price of powdered iron material above
	Labor cost	3.7	$	12.00	66.80	39.40	4.21	11.17	6.1	Described in the text
	Capital cost	3.7	$	0.68	0.84	0.76	2.54	3.10	2.8	Described in the text
	External - climate change	31.28	kg CO_2 eq	0.13	0.15	0.14	3.94	4.82	4.4	Described in the text
	External - freshwater eutrophication	1.58E-02	kg P eq	4.10	5.02	4.56	0.06	0.08	0.1	Described in the text
	External - ozone depletion	1.57E-06	kg CFC^{-11} eq	349	427	388	0.00	0.00	0.0	Described in the text

ENP/ synthesis methods/ AOF process	Materials /processes	amount	Unit	Min Unit Cost ($)	Max Unit Cost ($)	Mean Unit Cost ($)	Min Total Cost ($)	Max Total Cost ($)	Mean Total Cost ($)	Note on the unit cost
**Dithionite reduction:**	External - terrestrial acidification	0.12	kg SO_2 eq	8.64	10.56	9.60	1.05	1.29	1.2	Described in the text
	FeCl2 4H2O	3.56	kg	0.60	1.80	1.20	2.14	6.41	4.3	The range is obtained for industrial grade ferrous chloride from different manufacturers in China giving a price range of $0.6 to $1.8 per kg. This range is above the price in USA in 2019 for iron ore, $0.112, iron/steel slag, $0.0275, and iron/steel scrap, $0.226-$0.310 per kg as reported[90].
	Na2S2O4	6.69	kg	1.20	1.80	1.50	8.03	12.05	10.0	The range is obtained for industrial grade Sodium Dithionite Hydrate from different manufacturers in China.
	NaOH	0.0016	kg	0.20	0.60	0.40	0.00	0.00	0.0	The range is obtained for industrial grade 99% grade NaOH from different manufacturers in China.
	HCl	0.0026	kg	0.20	0.20	0.20	0.00	0.00	0.0	The range is obtained for industrial grade 30%-36% HCl from different manufacturers in China.
	Milli-Q water	200.0	kg	0.00	0.23	0.12	0.00	46.60	23.3	min, 0.00002 from Ref.[5] and max, 0.233, from industrial grade DI water supplied in 1000 kg tanks[91]
	N2 purging	0.77	kg	0.83	1.01	0.92	0.64	0.78	0.7	10% lower and higher than Ref.[5]
	Magnetic stirring	0.07	kWh	0.06	0.09	0.08	4.2E-03	0.01	0.0	The min is from Ref.[33] and the max is obtained for household electricity price in China[44]
	Storing under N2 environment	1.15	kg	0.92	0.92	0.92	1.06	1.06	1.1	10% lower and higher than Ref.[5]

ENP/ synthesis methods/ AOF process	Materials /processes	amount	Unit	Min Unit Cost ($)	Max Unit Cost ($)	Mean Unit Cost ($)	Min Total Cost ($)	Max Total Cost ($)	Mean Total Cost ($)	Note on the unit cost
	Labor cost	39.4	$	12.00	66.80	39.40	44.75	118.63	65.0	Described in the text
	Capital cost	39.4	$	0.68	0.84	0.76	26.94	32.93	29.9	Described in the text
	External - climate change	28.93	kg CO_2 eq	0.13	0.15	0.14	3.65	4.46	4.1	Described in the text
	External - freshwater eutrophication	1.69E-02	kg P eq	4.10	5.02	4.56	0.07	0.08	0.1	Described in the text
	External - ozone depletion	7.94E-06	kg CFC^{-11} eq	349	427	388	2.8E-03	3.4E-03	0.0	Described in the text
	External - terrestrial acidification	0.62	kg SO_2 eq	8.64	10.56	9.60	5.35	6.54	5.9	Described in the text
**Borohydride reduction**										
	FeCl3 6H2O	2.90	kg	0.30	0.90	0.60	0.87	2.61	1.7	The range is obtained for industrial grade ferrous chloride from different manufacturers in China giving a price range of $0.3 to $0.9 per kg. This range is above the price in USA in 2019 for iron ore, $0.112, iron/steel slag, $0.0275, and iron/steel scrap, $0.226-$0.310 per kg as reported[90].
	NaOH	0.44	kg	0.20	0.60	0.40	0.09	0.26	0.2	The range is obtained for industrial grade 99% grade NaOH from different manufacturers in China giving a price range of $0.2 to $0.6 per kg.
	HCl	0.0032	kg	0.15	0.25	0.20	4.8E-04	8.0E-04	0.0	The range is obtained for industrial grade 30%-36% HCl from different manufacturers in China giving a price range of $0.15 to $0.25 per kg.

ENP/ synthesis methods/ AOF process	Materials /processes	amount	Unit	Min Unit Cost ($)	Max Unit Cost ($)	Mean Unit Cost ($)	Min Total Cost ($)	Max Total Cost ($)	Mean Total Cost ($)	Note on the unit cost
	Milli-Q water	200.0	kg	2.0E-05	0.23	0.12	4.0E-03	46.6	23.3	min, 0.00002 from Ref.[5] and max, 0.233, from industrial grade DI water supplied in 1000 kg tanks following Ref.[91]
	NaBH4	0.65	kg	5.00	35.00	20.00	3.25	22.75	13.0	The range is obtained for industrial grade 98% Sodium Borohydride from different manufacturers in China and India giving a price range of $5 to $35 per kg.
	Magnetic stirring	0.07	kWh	0.06	0.09	0.08	0.00	0.01	0.0	The min is from Ref.[33] and the max is obtained for household electricity price in China[44]
	Labor cost	38.2	$	12.00	66.80	39.40	43.44	115.13	63.1	Described in the text
	Capital cost	38.2	$	0.68	0.84	0.76	26.14	31.95	29.0	Described in the text
	External - climate change	16.37	kg CO_2 eq	0.13	0.15	0.14	2.06	2.52	2.3	Described in the text
	External - freshwater eutrophication	6.88E-03	kg P eq	4.10	5.02	4.56	0.03	0.03	0.0	Described in the text
	External - ozone depletion	2.48E-06	kg CFC^{-11} eq	349	427	388	8.7E-04	1.1E-03	0.0	Described in the text
	External - terrestrial acidification	0.07	kg SO_2 eq	8.64	10.56	9.60	0.59	0.73	0.7	Described in the text
**Green synthesis methods either**	Virginia creeper	1.79	kg	0.15	0.25	0.20	0.27	0.45	0.4	The range is obtained for agricultural dried leaves and alfalfa hay.

**ENP/ synthesis methods/ AOF process**	**Materials /processes**	**amount**	**Unit**	**Min Unit Cost ($)**	**Max Unit Cost ($)**	**Mean Unit Cost ($)**	**Min Total Cost ($)**	**Max Total Cost ($)**	**Mean Total Cost ($)**	**Note on the unit cost**
**or**	Cofee	1.79	kg	1.00	8.00	4.50	1.79	14.35	8.1	The range is obtained for food-grade dried, raw or processed coffee beans.
**or**	Green tea	1.79	kg	2.80	16.00	9.40	5.02	28.71	16.9	The range is obtained for food-grade dried, raw or processed tea leaves.
	Pretreatment	17.9	kg	2.0E-05	8.1E-04	4.2E-04	3.6E-04	1.5E-02	0.0	min, 0.00002 from Ref.[5] and max, 0.00081, from average tap water price in China[92]
	Pretreatment	3.59	L	0.0E +00	0.0E +00	0.0E+00	0.0E +00	0.0E +00	0.0	Considered in energy cost and labor
	100 mL DI water	35.9	kg	2.0E-05	8.1E-04	4.2E-04	7.2E-04	2.9E-02	0.0	min, 0.00002 from Ref.[5] and max, 0.00081, from average tap water price in China[92]
	Boiling	0.05	kWh	6.0E-02	9.0E-02	7.5E-02	2.9E-03	4.3E-03	0.0	The min is from Ref.[33] and the max is obtained for household electricity price in China[44]
	Filter paper	3.84	kg	2.50	9.00	5.75	9.60	34.56	22.1	The range is obtained for food- and industrial-grade filter papers.
	Filtration energy	0.02	kWh	6.0E-02	9.0E-02	7.5E-02	1.1E-03	1.7E-03	0.0	The min is from Ref.[33] and the max is obtained for household electricity price in China[44]
	FeCl3.6H2O	1.00	kg	3.0E-01	9.0E-01	6.0E-01	3.0E-01	9.0E-01	0.6	The range is obtained for industrial grade ferrous chloride from different manufacturers in China giving a price range of $0.3 to $0.9 per kg. This range is above the price in USA in 2019 for iron ore, $0.112, iron/steel slag, $0.0275, and iron/steel scrap, $0.226-$0.310 per kg as reported.[90]
	Water	35.9	kg	2.0E-05	8.1E-04	4.2E-04	7.2E-04	2.9E-02	0.0	min, 0.00002 from Ref.[5] and max, 0.00081, from average tap water price in China[92]
	Stirring for 24 h	0.68	kWh	6.0E-02	9.0E-02	7.5E-02	4.1E-02	6.1E-02	0.1	The min is from Ref.[33] and the max is obtained for household electricity price in China[44]

ENP/ synthesis methods/ AOF process	Materials /processes	amount	Unit	Min Unit Cost ($)	Max Unit Cost ($)	Mean Unit Cost ($)	Min Total Cost ($)	Max Total Cost ($)	Mean Total Cost ($)	Note on the unit cost
	Labor cost	23.1	$	12.00	66.80	39.40	26.29	69.67	38.2	Described in the text
	Capital cost	23.1	$	0.68	0.84	0.76	15.82	19.34	17.6	Described in the text
	External - climate change	6.06	kg CO_2 eq	0.13	0.15	0.14	0.76	0.93	0.8	Described in the text
	External - freshwater eutrophication	4.02E-03	kg P eq	4.10	5.02	4.56	0.02	0.02	0.0	Described in the text
	External - ozone depletion	8.77E-07	kg CFC^{-11} eq	349.1	426.7	387.9	3.1E-04	3.7E-04	0.0	Described in the text
	External - terrestrial acidification	0.03	kg SO_2 eq	8.64	10.56	9.60	0.30	0.37	0.3	Described in the text
**SiO2**										
**Ball milling**										
	Natural sand without any pretreatment	2.00	kg	0.05	0.35	0.20	0.10	0.70	0.4	The range is obtained for industrial grade clean sand from different manufacturers in China.
	DI water	4.00	kg	2.0E-05	0.23	0.12	8.0E-05	0.93	0.5	min, 0.00002 from Ref.[5] and max, 0.233, from industrial grade DI water supplied in 1000 kg tanks following Ref.[91]
	Vibratory sieving	0.0037	kWh	0.06	0.09	0.08	2.2E-04	3.3E-04	0.0	The min is from Ref.[33] and the max is obtained for household electricity price in China[44]
	Ball milling	46.6	kWh	0.06	0.09	0.08	2.79	4.19	3.5	The min is from Ref.[33] and the max is obtained for household electricity price in China[44]
	Labor cost	4.4	$	12.00	66.80	39.40	4.95	13.13	7.2	Described in the text
	Capital cost	4.4	$	0.68	0.84	0.76	2.98	3.64	3.3	Described in the text

ENP/ synthesis methods/ AOF process	Materials /processes	amount	Unit	Min Unit Cost ($)	Max Unit Cost ($)	Mean Unit Cost ($)	Min Total Cost ($)	Max Total Cost ($)	Mean Total Cost ($)	Note on the unit cost
	External - climate change	32.75	kg CO_2 eq	0.13	0.15	0.14	4.13	5.04	4.6	Described in the text
	External - freshwater eutrophication	1.68E-02	kg P eq	4.10	5.02	4.56	0.07	0.08	0.1	Described in the text
	External - ozone depletion	1.53E-06	kg CFC^{-11} eq	349.1	426.7	387.9	5.3E-04	6.5E-04	0.0	Described in the text
	External - terrestrial acidification	0.13	kg SO_2 eq	8.64	10.56	9.60	1.11	1.36	1.2	Described in the text
**Green - rice husk**										
	Rice husk	1.04	kg	0.05	0.15	0.10	0.05	0.16	0.1	The range is obtained for agricultural rice husk from different producers in China.
	Water	103.5	kg	2.0E-05	8.1E-04	4.2E-04	2.1E-03	8.4E-02	0.0	min, 0.00002 from Ref.[5] and max, 0.00081, from average tap water price in China[92]
	Drying agricultural products in air	2.07	kg	0	0	0	0	0	0.0	Considered in the cost of rice husk, energy and labor
	Drying	0.25	kWh	6.0E-02	9.0E-02	7.5E-02	1.5E-02	2.2E-02	0.0	The min is from Ref.[33] and the max is obtained for household electricity price in China[44]
	Burning inside muffle furnace	0.10	kWh	6.0E-02	9.0E-02	7.5E-02	5.8E-03	8.7E-03	0.0	The min is from Ref.[33] and the max is obtained for household electricity price in China[44]
	Burning inside muffle furnace	0.10	kWh	6.0E-02	9.0E-02	7.5E-02	5.8E-03	8.7E-03	0.0	The min is from Ref.[33] and the max is obtained for household electricity price in China[44]
	Water	51.8	kg	2.0E-05	8.1E-04	4.2E-04	1.0E-03	4.2E-02	0.0	min, 0.00002 from Ref.[5] and max, 0.00081, from average tap water price in China[92]

ENP/ synthesis methods/ AOF process	Materials /processes	amount	Unit	Min Unit Cost ($)	Max Unit Cost ($)	Mean Unit Cost ($)	Min Total Cost ($)	Max Total Cost ($)	Mean Total Cost ($)	Note on the unit cost
	HNO3	3.26	kg	3.5E-01	4.5E-01	4.0E-01	1.1E +00	1.5E +00	1.3	The range is obtained for industrial grade 68% Nitric Acid from different manufacturers in China giving a price range of $0.3 to $0.45 per kg.
	Stirring	0.41	kWh	6.0E-02	9.0E-02	7.5E-02	2.4E-02	3.7E-02	0.0	The min is from Ref.[33] and the max is obtained for household electricity price in China[44]
	Water	103.5	kg	2.0E-05	8.1E-04	4.2E-04	2.1E-03	8.4E-02	0.0	min, 0.00002 from Ref.[5] and max, 0.00081, from average tap water price in China[92]
	Ethanol	1.04	kg	0.85	1.50	1.18	0.88	1.55	1.2	The range is obtained for industrial grade ethanol from different producers in China.
	Filter paper	2.22	kg	2.50	9.00	5.75	5.54	19.94	12.7	The range is obtained for food- and industrial-grade filter papers.
	Filtration energy	0.01	kWh	6.0E-02	9.0E-02	7.5E-02	6.5E-04	9.8E-04	0.0	The min is from Ref.[33] and the max is obtained for household electricity price in China[44]
	Drying	0.06	kWh	6.0E-02	9.0E-02	7.5E-02	3.7E-03	5.6E-03	0.0	The min is from Ref.[33] and the max is obtained for household electricity price in China[44]
	Ball milling	6.02	kWh	6.0E-02	9.0E-02	7.5E-02	3.6E-01	0.54	0.5	The min is from Ref.[33] and the max is obtained for household electricity price in China[44]
	Labor cost	16.0	$	12.00	66.80	39.40	18.17	48.17	26.4	Described in the text
	Capital cost	16.0	$	0.68	0.84	0.76	10.94	13.37	12.2	Described in the text
	External - climate change	20.51	kg CO_2 eq	0.13	0.15	0.14	2.58	3.16	2.9	Described in the text
	External - freshwater eutrophication	6.17E-03	kg P eq	4.10	5.02	4.56	2.5E-02	3.1E-02	0.0	Described in the text

ENP/ synthesis methods/ AOF process	Materials /processes	amount	Unit	Min Unit Cost ($)	Max Unit Cost ($)	Mean Unit Cost ($)	Min Total Cost ($)	Max Total Cost ($)	Mean Total Cost ($)	Note on the unit cost
	External - ozone depletion	9.12E-07	kg CFC^{-11} eq	349.1	426.6	387.9	3.2E-04	3.9E-04	0.0	Described in the text
	External - terrestrial acidification	0.09	kg SO_2 eq	8.64	10.56	9.60	0.80	0.98	0.9	Described in the text
**Green - wheat husk**										
	Wheat husk	1.33	kg	0.20	0.32	0.26	0.27	0.43	0.3	The range is obtained for food grade wheat bran from different producers in China.
	Water for washing	133.3	kg	2.0E-05	8.1E-04	4.2E-04	2.7E-03	0.11	0.1	min, 0.00002 from Ref.[5] and max, 0.00081, from average tap water price in China[92]
	Drying	2.67	kg	0	0	0	0	0	0.0	Considered in the cost of wheat bran, energy and labor
	Drying at 60 degree C for 24 h	0.64	kWh	6.0E-02	9.0E-02	7.5E-02	3.8E-02	5.8E-02	0.0	min, 0.06 from Ref.[33] and max, 0.09, for household electricity price in China[44]
	Water	40.0	kg	2.0E-05	8.1E-04	4.2E-04	8.0E-04	3.2E-02	0.0	min, 0.00002 from Ref.[5] and max, 0.00081, from average tap water price in China[92]
	H2SO4	8.02	kg	0.20	0.32	0.26	1.60	2.57	2.1	The range is obtained for industrial grade Sulfuric acid from different producers in China.
	Heating and vigorous stirring at 90 degree C for 3 h	1.63	kWh	6.0E-02	9.0E-02	7.5E-02	0.10	0.15	0.1	The min is from Ref.[33] and the max is obtained for household electricity price in China[44]
	Filter paper	2.85	kg	2.50	9.00	5.75	7.13	25.68	16.4	The range is obtained for food- and industrial-grade filter papers.

ENP/ synthesis methods/ AOF process	Materials /processes	amount	Unit	Min Unit Cost ($)	Max Unit Cost ($)	Mean Unit Cost ($)	Min Total Cost ($)	Max Total Cost ($)	Mean Total Cost ($)	Note on the unit cost
	Filtration energy	0.01	kWh	6.0E-02	9.0E-02	7.5E-02	8.4E-04	1.3E-03	0.0	The min is from Ref.[33] and the max is obtained for household electricity price in China[44]
	Calcination	0.26	kWh	6.0E-02	9.0E-02	7.5E-02	1.5E-02	2.3E-02	0.0	The min is from Ref.[33] and the max is obtained for household electricity price in China[44]
	Washing the resulted powder with distilled water	13.3	kg	2.0E-05	0.23	0.12	2.7E-04	3.11	1.6	min, 0.00002 from Ref.[5] and max, 0.233, from industrial grade DI water supplied in 1000 kg tanks following Ref.[91]
	Labor cost	20.7	$	12.00	66.80	39.40	23.47	62.21	34.1	Described in the text
	Capital cost	20.7	$	0.68	0.84	0.76	14.13	17.27	15.7	Described in the text
	External - climate change	6.47	kg CO_2 eq	0.13	0.15	0.14	0.82	1.00	0.9	Described in the text
	External - freshwater eutrophication	3.16E-03	kg P eq	4.10	5.02	4.56	1.3E-02	1.6E-02	0.0	Described in the text
	External - ozone depletion	4.93E-07	kg CFC^{-11} eq	349.1	426.7	387.9	1.7E-04	2.1E-04	0.0	Described in the text
	External - terrestrial acidification	0.03	kg SO_2 eq	8.64	10.56	9.60	0.29	0.35	0.3	Described in the text
**Al_2O_3**										
**Ball milling**										
	Aluminium powder	1.80	kg	0.5	3.4	1.95	0.9	6.1	3.5	The range is obtained for industrial grade Aluminum Oxide Powder from different manufacturers in China.

ENP/ synthesis methods/ AOF process	Materials /processes	amount	Unit	Min Unit Cost ($)	Max Unit Cost ($)	Mean Unit Cost ($)	Min Total Cost ($)	Max Total Cost ($)	Mean Total Cost ($)	Note on the unit cost
	Initial grinding	0.44	kWh	6.0E-02	9.0E-02	7.5E-02	2.6E-02	4.0E-02	0.0	The min is from Ref.[33] and the max is obtained for household electricity price in China[44]
	Parafin oil	0.83	kg	0.50	2.00	1.25	0.41	1.65	1.0	The range is obtained for industrial grade non-ionic surfactant from different manufacturers in China.
	Air addition	0.20	m^3	4.53	5.54	5.04	0.93	1.13	1.0	Based on price for Industrial grade 30-L cylinders with the weight of 6.73 kg from Ref.[93]
	Ball milling	16.5	kWh	0.06	0.09	0.08	0.99	1.48	1.2	The min is from Ref.[33] and the max is obtained for household electricity price in China[44]
	Labor cost	6.8	$	12.00	66.80	39.40	7.78	20.61	11.3	Described in the text
	Capital cost	6.8	$	0.68	0.84	0.76	4.68	5.72	5.2	Described in the text
	External - climate change	16.78	kg CO_2 eq	0.13	0.15	0.14	2.11	2.58	2.3	Described in the text
	External - freshwater eutrophication	8.76E-03	kg P eq	4.10	5.02	4.56	3.6E-02	4.4E-02	0.0	Described in the text
	External - ozone depletion	8.55E-07	kg CFC^{-11} eq	349.1	426.7	387.9	3.0E-04	3.6E-04	0.0	Described in the text
	External - terrestrial acidification	0.07	kg SO_2 eq	8.64	10.56	9.60	0.62	0.76	0.7	Described in the text
**Green - Algae**										
	Algae	0.07	kg	2.86	13.60	8.23	0.19	0.91	0.5	The range (2.86 to 13.6 $/kg) taken from Tredici et al. (2016)
	Tap water for washing	66.7	kg	2.0E-05	8.1E-04	4.2E-04	1.3E-03	5.4E-02	0.0	min, 0.00002 from Ref.[5] and max, 0.00081, from average tap water price in China[92]

ENP/ synthesis methods/ AOF process	Materials /processes	amount	Unit	Min Unit Cost ($)	Max Unit Cost ($)	Mean Unit Cost ($)	Min Total Cost ($)	Max Total Cost ($)	Mean Total Cost ($)	Note on the unit cost
	DI water for washing	6.67	kg	2.0E-05	2.3E-01	0.12	1.3E-04	1.55	0.8	min, 0.00002 from Ref.[5] and max, 0.233, from industrial grade DI water supplied in 1000 kg tanks following Ref.[91]
	Drying in shade for 1 week	0.07	kg	0.0E +00	0.0E +00	0.0E+ 00	0.0E +00	0.0E +00	0.0	Included in the cost of algae production and labor
	Grinding	0.02	kWh	6.0E-02	9.0E-02	7.5E-02	9.8E-04	1.5E-03	0.0	The min is from Ref.[33] and the max is obtained for household electricity price in China[44]
	Heating	3.67	kWh	0.06	9.0E-02	7.5E-02	0.22	0.33	0.3	The min is from Ref.[33] and the max is obtained for household electricity price in China[44]
	Aluminum Sulfate	3.36	kg	0.11	0.35	0.23	0.37	1.17	0.8	The range is obtained for industrial grade Aluminium sulfate (Alum powder) from different manufacturers in China.
	Stirring for 1 h	0.09	kWh	0.06	9.0E-02	7.5E-02	5.2E-03	7.7E-03	0.0	The min is from Ref.[33] and the max is obtained for household electricity price in China[44]
	Heating at 70 C for 25 min	3.33	kWh	0.06	9.0E-02	7.5E-02	0.20	0.30	0.2	The min is from Ref.[33] and the max is obtained for household electricity price in China[44]
	Oven driying at 180 C for 1 h	0.32	kWh	0.06	9.0E-02	7.5E-02	1.9E-02	2.8E-02	0.0	The min is from Ref.[33] and the max is obtained for household electricity price in China[44]
	Calcination at 1200 for 2 h	4.09	kWh	0.06	9.0E-02	7.5E-02	0.25	0.37	0.3	The min is from Ref.[33] and the max is obtained for household electricity price in China[44]
	Labor cost	3.0	$	12.00	66.80	39.40	3.40	9.00	4.9	Described in the text
	Capital cost	3.0	$	0.68	0.84	0.76	2.04	2.50	2.3	Described in the text
	External - climate change	10.76	kg CO_2 eq	0.13	0.15	0.14	1.36	1.66	1.5	Described in the text

ENP/ synthesis methods/ AOF process	Materials /processes	amount	Unit	Min Unit Cost ($)	Max Unit Cost ($)	Mean Unit Cost ($)	Min Total Cost ($)	Max Total Cost ($)	Mean Total Cost ($)	Note on the unit cost
	External - freshwater eutrophication	5.67E-03	kg P eq	4.10	5.02	4.56	2.3E-02	2.8E-02	0.0	Described in the text
	External - ozone depletion	5.60E-07	kg CFC^{-11} eq	349.1	426.7	387.9	2.0E-04	2.4E-04	0.0	Described in the text
	External - terrestrial acidification	0.06	kg SO_2 eq	8.64	10.56	9.60	0.49	0.60	0.5	Described in the text
**ZnO**										
**Ball milling**										
	Zinc oxide (powder)	1.80	kg	1.45	4.50	2.98	2.61	8.10	5.4	The range is obtained for industrial grade Zinc Oxide Powder from different manufacturers in China.
	Initial grinding	0.44	kWh	6.0E-02	9.0E-02	7.5E-02	2.6E-02	4.0E-02	0.0	The min is from Ref.[33] and the max is obtained for household electricity price in China[44]
	Cooling with chilled water	3.06	kWh	6.0E-02	9.0E-02	7.5E-02	0.18	0.28	0.2	The min is from Ref.[33] and the max is obtained for household electricity price in China[44]
	Water needed for cooling	288.0	L	2.0E-05	8.1E-04	4.2E-04	5.8E-03	2.3E-01	0.1	min, 0.00002 from Ref.[5] and max, 0.00081, from average tap water price in China[92]
	Ball milling	58.5	kWh	6.0E-02	9.0E-02	7.5E-02	3.51	5.26	4.4	The min is from Ref.[33] and the max is obtained for household electricity price in China[44]
	Labor cost	10.1	$	12.00	66.80	39.40	11.50	30.49	16.7	Described in the text
	Capital cost	10.1	$	0.68	0.84	0.76	6.92	8.46	7.7	Described in the text
	External - climate change	46.32	kg CO_2 eq	0.13	0.15	0.14	5.84	7.13	6.5	Described in the text

ENP/ synthesis methods/ AOF process	Materials /processes	amount	Unit	Min Unit Cost ($)	Max Unit Cost ($)	Mean Unit Cost ($)	Min Total Cost ($)	Max Total Cost ($)	Mean Total Cost ($)	Note on the unit cost
	External - freshwater eutrophication	2.35E-02	kg P eq	4.10	5.02	4.56	0.10	0.12	0.1	Described in the text
	External - ozone depletion	2.31E-06	kg CFC^{-11} eq	349.1	426.7	387.9	8.1E-04	9.9E-04	0.0	Described in the text
	External - terrestrial acidification	0.18	kg SO_2 eq	8.64	10.56	9.60	1.57	1.92	1.7	Described in the text
**CeO2** **Ball milling**										
	Cerium	1.80	kg	1.00	18.00	9.50	1.80	32.40	17.1	The range is obtained for industrial grade Cerium Oxide Powder from different manufacturers in China.
	Grinding	0.44	kWh	6.0E-02	9.0E-02	7.5E-02	2.6E-02	4.0E-02	0.0	The min is from Ref.[33] and the max is obtained for household electricity price in China[44]
	Cooling with chilled water	4.59	kWh	6.0E-02	9.0E-02	7.5E-02	0.28	0.41	0.3	The min is from Ref.[33] and the max is obtained for household electricity price in China[44]
	Water needed for cooling	432.0	kg	2.0E-05	8.1E-04	4.2E-04	8.6E-03	0.35	0.2	min, 0.00002 from Ref.[5] and max, 0.00081, from average tap water price in China[92]
	Ball milling	86.6	kWh	6.0E-02	9.0E-02	7.5E-02	5.20	7.80	6.5	The min is from Ref.[33] and the max is obtained for household electricity price in China[44]
	Labor cost	24.2	$	12.00	66.80	39.40	27.45	72.75	39.9	Described in the text
	Capital cost	24.2	$	0.68	0.84	0.76	16.52	20.19	18.4	Described in the text
	External - climate change	150.63	kg CO_2 eq	0.13	0.15	0.14	18.98	23.20	21.1	Described in the text

ENP/ synthesis methods/ AOF process	Materials /processes	amount	Unit	Min Unit Cost ($)	Max Unit Cost ($)	Mean Unit Cost ($)	Min Total Cost ($)	Max Total Cost ($)	Mean Total Cost ($)	Note on the unit cost
	External - freshwater eutrophication	7.82E-02	kg P eq	4.10	5.02	4.56	0.32	0.39	0.4	Described in the text
	External - ozone depletion	2.19E-05	kg CFC^{-11} eq	349.1	426.7	387.9	7.7E-03	9.4E-03	0.0	Described in the text
	External - terrestrial acidification	0.61	kg SO_2 eq	8.64	10.56	9.60	5.24	6.40	5.8	Described in the text
**Polymer (for coating the surface of 1 kg ENP)**										
**either**	Carboxymethyl cellulose (CMC)	0.17	kg	0.50	5.00	2.75	8.3E-02	0.83	0.5	The range is obtained for industrial and food grade Carboxymethyl Cellulose Powder from different manufacturers in China.
**or**	surfactant (ethylene oxide derivate)	0.17	kg	0.50	2.00	1.25	8.3E-02	0.33	0.2	The range is obtained for industrial grade non-ionic surfactant from different manufacturers in China.
**or**	surfactant (fatty acid derivate)	0.17	kg	0.50	2.00	1.25	8.3E-02	0.33	0.2	The range is obtained for industrial grade non-ionic surfactant from different manufacturers in China.
	NaHCO3	0.11	kg	0.19	0.51	0.35	1.9E-02	0.05	0.0	The range is obtained for industrial and food grade Sodium Bicarbonate from different manufacturers in China.
	mixing for 48 h at 30 rpm	0.02	kWh	0.06	0.09	0.08	1.1E-03	1.7E-03	0.0	The min is from Ref.[33] and the max is obtained for household electricity price in China[44]

ENP/ synthesis methods/ AOF process	Materials /processes	amount	Unit	Min Unit Cost ($)	Max Unit Cost ($)	Mean Unit Cost ($)	Min Total Cost ($)	Max Total Cost ($)	Mean Total Cost ($)	Note on the unit cost
	Centrifugation for 80 min at 27500 rpm	4.00	kWh	0.06	0.09	0.08	0.24	0.36	0.3	The min is from Ref.[33] and the max is obtained for household electricity price in China[44]
	Labor cost	0.8	$	12.00	66.80	39.40	0.90	2.40	1.3	Described in the text
	Capital cost	0.8	$	0.68	0.84	0.76	0.54	0.67	0.6	Described in the text
	External - climate change	3.60	kg CO_2 eq	0.13	0.15	0.14	0.45	0.55	0.5	Described in the text
	External - freshwater eutrophication	1.74E-03	kg P eq	4.10	5.02	4.56	7.2E-03	8.7E-03	0.0	Described in the text
	External - ozone depletion	2.39E-07	kg CFC^{-11} eq	349.1	426.7	387.9	8.4E-05	1.0E-04	0.0	Described in the text
	External - terrestrial acidification	0.02	kg SO_2 eq	8.64	10.56	9.60	0.13	0.16	0.1	Described in the text
**AOF Operation**										
	Ship for transport	12.2	t.km	4.6E-02	6.1E-02	5.4E-02	0.56	0.75	0.7	Distance from production site of the materials (assumed China) to the delivery site (assumed Southern Ocean). Further details are given in the text.
**either**	Ship for distribution	6.8	t.km	4.6E-02	6.1E-02	5.4E-02	0.31	0.41	0.4	Delivery via smaller ships at a rate of 0.451 km/kg, leading to a travel distance of 902 to 4510 km multiplied by five for expected higher efficiency of using ENPs. Further details are given in the text.
**or**	Alternative Aircraft for distribution and monitoring	0.7	km2	1300.00	3879.55	2589.78	939.05	2802.38	1870.7	Based on fairs for aerial spray (agricultural aircraft)[25,26]

ENP/ synthesis methods/ AOF process	Materials /processes	amount	Unit	Min Unit Cost ($)	Max Unit Cost ($)	Mean Unit Cost ($)	Min Total Cost ($)	Max Total Cost ($)	Mean Total Cost ($)	Note on the unit cost
	Ship for monitoring	6.8	t.km	4.6E-02	6.1E-02	5.4E-02	0.31	0.41	0.4	Assuming the same travel distance as the distribution.
	Lorry for land transport	0.30	t.km	0	0	0	0	0	0.0	Included in the cost of ship transport
	Other miscellaneous transportation costs including land transport	19.0	t.km	3.5E-03	4.2E-03	3.8E-03	6.7E-02	7.9E-02	0.1	handling in and out ($26.6 per ton), storage ($57.1 per ton), vanning/devanning ($16.6 per ton), and other administration charges such as CFS charges, Original Documentation fee, re-weighting, ENS Fee Exempt, and Export Customs Clearance Fee ($6.77 per ton)[30]
	Labor (professional)	0.265	hour-person	3.74	6.62	5.18	0.99	1.75	1.4	Assuming a delivery rate of 3.77 kg/h and that one professional and one non-professional worker is needed for operation. Labor hourly wage range is taken from Ref.[31]
	Labor (non-profession)	0.265	hour-person	2.08	3.68	2.88	0.55	0.98	0.8	Assuming a delivery rate of 3.77 kg/h and that one professional and one non-professional worker is needed for operation. Labor hourly wage range is taken from
	Monitoring/sample measurement	0.265	hour-person	3.74	6.62	5.18	0.99	1.75	1.4	Assuming a delivery rate of 3.77 kg/h and that one professional person is needed for sampling and measurement during the operation. Labor hourly wage range is taken from Ref.[31]
	External - climate change	1.6	kg CO_2 eq	0.126	0.154	0.14	0.20	0.24	0.22	Described in the text
	External - freshwater eutrophication	5.8E-05	kg P eq	4.104	5.016	4.56	2.4E-04	2.9E-04	2.6E-04	Described in the text

ENP/ synthesis methods/ AOF process	Materials /processes	amount	Unit	Min Unit Cost ($)	Max Unit Cost ($)	Mean Unit Cost ($)	Min Total Cost ($)	Max Total Cost ($)	Mean Total Cost ($)	Note on the unit cost
	External - ozone depletion	2.6E-07	kg CFC^{-11} eq	349.1	426.6	387.9	9.0E-05	1.1E-04	1.0E-04	Described in the text
	External - terrestrial acidification	3.9E-02	kg SO_2 eq	8.64	10.56	9.6	3.4E-01	0.41	0.37	Described in the text
**Soluble iron**	FeSO4.7H2O	5.0	kg	0.04	0.65	0.35	0.20	3.24	1.7	The range is obtained for industrial and food grade Ferrous Sulfate from different manufacturers in China.
	HCl	0.014	kg	0.15	0.25	0.20	2E-03	3E-03	0.0	The range is obtained for industrial grade 30%-36% HCl from different manufacturers in China.
	Labor cost	1.7	$	12.00	66.80	39.40	1.96	5.18	2.8	Described in the text
	Capital cost	1.7	$	0.68	0.84	0.76	1.18	1.44	1.3	Described in the text
	External - climate change	1.39	kg CO_2 eq	0.13	0.15	0.14	0.17	0.21	0.2	Described in the text
	External - freshwater eutrophication	8.18E-04	kg P eq	4.10	5.02	4.56	3E-03	4E-03	3.7E-03	Described in the text
	External - ozone depletion	9.20E-08	kg CFC^{-11} eq	349.1	426.7	387.9	3E-05	4E-05	3.6E-05	Described in the text
	External - terrestrial acidification	0.01	kg SO_2 eq	8.64	10.56	9.60	7E-02	8E-02	0.1	Described in the text

Table S4. Summary of various environmental impacts obtained from our life cycle assessment. These results are minimum, maximum, and average of impacts obtained for various ENP synthesis methods, iron sulfate, polymers, and AOF processes that are investigated in the present study.

Impact category	Unit	Min	Min, (ENP synthesis methods)	Max	Mean	Case with min Impact of ENP synthesis method	Case with max Impact
**Agricultural land occupation**	m^2a	7.2E-03	0.40	41.3	8.86	Al_2O_3 (Green) Algae	NZVI (Green) tea
**Climate change**	kg CO_2 eq	1.39	6.06	151	23.2	NZVI (Green) Creeper	CeO_2 ball mill
**Fossil depletion**	kg oil eq	0.38	1.65	41.6	6.35	NZVI (Green) Creeper	CeO_2 ball mill
**Freshwater ecotoxicity**	kg 1,4-DB eq	1.0E-02	0.14	3.45	0.64	SiO_2 (Green) wheat husk	CeO_2 ball mill
**Freshwater eutrophication**	kg P eq	5.8E-05	3.2E-03	7.8E-02	1.2E-02	SiO_2 (Green) wheat husk	CeO_2 ball mill
**Human toxicity**	kg 1,4-DB eq	0.10	2.90	65.2	10.35	SiO_2 (Green) wheat husk	CeO_2 ball mill
**Ionising radiation**	kBq U235 eq	9.3E-02	0.80	23.2	3.20	SiO_2 (Green) wheat husk	CeO_2 ball mill
**Marine ecotoxicity**	kg 1,4-DB eq	1.1E-02	0.13	3.03	0.56	SiO_2 (Green) wheat husk	CeO_2 ball mill
**Marine eutrophication**	kg N eq	3.6E-04	2.4E-03	0.11	1.3E-02	Al_2O_3 (Green) Algae	NZVI (Green) coffee
**Metal depletion**	kg Fe eq	5.8E-02	0.28	161	8.94	SiO_2 (Green) wheat husk	NZVI dithionite
**Natural land transformation**	m^2	3.1E-04	1.0E-03	2.3E-02	3.9E-03	SiO_2 (Green) wheat husk	CeO_2 ball mill
**Ozone depletion**	kg CFC^{-11} eq	9.2E-08	4.9E-07	2.2E-05	2.3E-06	SiO_2 (Green) wheat husk	CeO_2 ball mill
**Particulate matter formation**	kg PM10 eq	4.4E-03	1.7E-02	4.0E-01	6.6E-02	SiO_2 (Green) wheat husk	CeO_2 ball mill
**Photochemical oxidant formation**	kg NMVOC	6.0E-03	2.6E-02	0.42	7.4E-02	SiO_2 (Green) wheat husk	CeO_2 ball mill
**Terrestrial acidification**	kg SO_2 eq	8.0E-03	3.3E-02	0.62	0.13	SiO_2 (Green) wheat husk	NZVI dithionite

**Terrestrial ecotoxicity**	kg 1,4-DB eq	1.0E-04	1.1E-03	0.70	5.6E-02	Al_2O_3 (Green) Algae	Al_2O_3 ball mill
**Urban land occupation**	m^2a	3.3E-02	0.52	1822	94.6	NZVI dithionite	NZVI (Green) coffee
**Water depletion**	m^3	1.0E-03	0.13	46.2	2.71	Al_2O_3 (Green) Algae	CeO_2 ball mill

Table S5. Summary of the results of our life cycle costing analysis (left side) for different ENPs and iron sulfate compared with the respective current market prices from small-scale manufacturers (right side).

	**Present LCC analysis***			**Market prices****		
	**Min total cost ($)**	**Max total cost ($)**	**Mean total cost ($)**	**Min total cost ($)**	**Max total cost ($)**	**Mean total cost ($)**
**Iron ENPs**	14	230	83	120	341	244
**SiO_2**	16	113	50	118	186	146
**Al_2O_3**	8.6	40	19	85	189	142
**ZnO**	32	62	43	95	176	148
**CeO_2**	75.8	164	110	138	321	245
**Iron sulfate*****	3.6	10	6.1	1.8	2.8	2.4
**Average of all ENPs**	29	122	61	111	242	185

* The min, max, and mean values are the minimum of minimum, maximum of maximum, and average of mean costs, respectively, obtained of different methods of each ENP types.

** The prices were obtained by inquiring quotes from various current manufacturers of ENPs including Alfa Aesar, nanografi, NanoShel, SAT nano, SS Nano, and US Research Nanomaterials , and current vendors of iron sulfate such as Agrigem, APC Pure, Vitax, and Pro-Kleen in January 2020. We then selected three of the vendors/manufacturers which offered the lowest prices for each ENP type, and reported the average of those prices. Almost all of these manufacturers work at small-scale production of ENPs (i.e., per kg) rather than large scale (per ton).

***In analyzing the LCC for iron sulfate, we have included the costs for acidifying (dissolving) iron sulfate in water as well as capital and labor costs in order to be consistent with other ENPs. Thus, the market price for iron sulfate is lower than our cost estimates although for ENPs the our cost estimates is 2 to 7 times lower than the mean market prices.

Table S6. Summary of selected studies on the toxicity of different engineered nanoparticles to aquatic biota mainly relevant to the marine ecosystem.

ENP	Organism	dose range investigated ($\mu g.L^{-1}$)	safe–toxic dose threshold ($\mu g.L^{-1}$)	criteria value ($\mu g.L^{-1}$)	criteria type used other than EC_{50}	major toxicity	Ref.
**TiO_2**	zebra mussels, Dreissena polymorpha	7–830				increasing Ti tissue concentration	[94]
**TiO_2**	Daphnia magna	45000				Highest uptake for aggregated ENPs	[95]
**TiO_2**	Daphnia magna	25000				larger TiO_2 ENPs were taken up faster	[96]
**TiO_2**	Ceriodaphnia dubia	1000–64000		>64000		concentrM10 dependent up take	[97]
**TiO_2**	Daphnia magna	1000–10000		5500–>500000		mortality	[98]
**TiO_2**	marine phytoplanktons diatoms, chlorophytes, and prymnesiophytes	10–1000				no effect on growth rate	[99]
**TiO_2**	zebrafish: Danio rerio	10–250				alteration of lipid accumulation and ROS in embryos	[100]
**TiO_2**	zebrafish larvae	100–10000				Accumulation in brain, cell death, doe–dependent toxicity	[101]
**TiO_2**	zebrafish (Danio rerio)	1000–4000				negatively affected spermatogenic cells and testicular morphology at high dozes	[102]

ENP	Organism	dose range investigated (μg.L^{-1})	safe–toxic dose threshold (μg.L^{-1})	criteria value (μg.L^{-1})	criteria type used other than EC_{50}	major toxicity	Ref.
**TiO_2**	Cyanobacteria: Anabaena variabilis	500–250000		420–620		reduced growth rate; dose–dependent effect	[103]
**TiO_2**	Lugworm (Arenicola marina)	1–3 g/kg				DNA damage	[104]
**TiO_2**	mussel (Mytilus galloprovincialis)	50–5000				digestive glands	[105]
**TiO_2**	abalone (Haliotis diversicolor supertexta)	100–10000	1000	>10000		not acutely toxic; Oxidative stress	[106]
**TiO_2**	abalone (Haliotis diversicolor supertexta) Embryos	2000–250000	10	56900–345800		hatching inhibition and malformations	[107]
**TiO_2**	Daphnia magna	12500–100000	100000	>100000		Immobilization	[108]
**TiO_2**	Microlalgae: Chlorella sp.	0–1000000		4900–48000	IC_{50}	growth inhibition	[109]
**TiO_2**	Daphnia magna	100–100000		460–100000		immobilization; mortality; and high level of bioaccumulation	[110]
**TiO_2**	Daphnia magna			>100000	EC_{50}	impacts on mortality and reproduction	[111]
**TiO_2**	Daphnia magna			35306–143387	EC_{50} and LC_{50}	immobilization and mortality	[112]
**TiO_2**	microalgae Pseudokirchneriella subcapitata	50–2000		2500–241000		growth inhibition	[59]
**TiO_2**	green alga Chlamydomonas reinhartii	1–100000	1000	–10000		growth inhibition; oxidative stress	[113]

ENP	Organism	dose range investigated (μg.L^{-1})	safe–toxic dose threshold (μg.L^{-1})	criteria value (μg.L^{-1})	criteria type used other than EC_{50}	major toxicity	Ref.
**TiO_2**	Artemia cysts hatching	10–100000		18940	LC_{50}	mortality due to NPs clogging the gut; hatched cysts show increased oxidative stress	[114]
**TiO_2**	D. tertiolecta (Chlorophyceae: Chlamydomonadales)	125000–1000000		20000–95000	EC_{50}	toxicity starting from oxidative stress generation; ROS production the main cause of growth inhibition	[115]
**TiO_2**	marine microalgae Dunaliella tertiolecta	1000–100000		186220–188880	EC_{50}	toxic action due to cell entrapment and agglomeration	[116]
**CuO**	Macoma balthica	200				Body burden increase	[117]
**CuO**	Leptocheirus plumulosus	500–2000 ug/g sediment		8.6 μg/g sediment		Body burden increase	[118]
**Cu**	Daphnia magna	NA		40–1130	LC_{50}	concentration dependent uptake; mortality	[119]
**CuO**	Lymnaea stag nalis	0.3–2466				bioaccumulation	[120]
**CuO**	Potamopyrgus antipodarum	240 μg/g sediments				higher body burden	[121]
**CuO**	Potamopyrgus antipodarum	240 μg/g sediments				Increased Cu content in tissue	[122]
**CuO**	zooplankton (Artemia salina) and goldfish (Carassius auratus)	1000–10000				Accumulation in intestine, gills, and liver	[123]
**Cu**	zebrafish embryos	150–1000				developmental defects during zebrafish embryogenesis	[124]
**CuO**	zebrafish	8–15910	12700	>15910		Embryo mortality and hatching decrease; no toxicity to cells	[125]
**Cu**	zebrafish	250				down–regulating Wnt signalling	[126]

ENP	Organism	dose range investigated (μg.L^{-1})	safe–toxic dose threshold (μg.L^{-1})	criteria value (μg.L^{-1})	criteria type used other than EC_{50}	major toxicity	Ref.
**CuO**	mussel: Mytilus edulis	400000–1000000				impact on lysosomal membrane stability	[127]
**CuO**	Ragworm (Hediste diversicolor); Bivalve mollusc (Scrobicularia plana)					Cholinesterase activity reduction	[128]
**CuO**	Daphnia magna; Thamnocephalus platyurus			2100–3200		extracellular ROS damaging cell membranes	[129]
**CuO**	Daphnia magna; Thamnocephalus platyurus			90000–224000	EC_{50} or LC_{50}	toxicity induced by dissolved metal ions	[130]
**CuO**	Artemia cysts hatching	100–100000		20920	LC_{50}	mortality due to NPs clogging the gut; hatched cysts show increased oxidative stress	[114]
**Cu**	Daphnia magna	500–1000		30–93	LC_{50}	Concentration dependent uptake	[131]
**CeO_2**	Drissena poly morpha	10–100				Body burden increase	[132]
**CeO_2**	brine shrimp Artemia salina	10000–1e6				accumulated in gut; no significant mortality	[133]
**CeO_2**	Sea urchin (Paracentrotus lividus)	100–10000				behavioral impairments; oxidative stress increase	[134]
**CeO_2**	mussels Mytilus galloprovincialis	1000–10000				accumulating only a small fraction in tissues	[135]
**CeO_2**	Daphnia pulex					40–100% uptake	[136]
**CeO_2**	Daphnia similis and Daphnia pulex	1000–100000		260–91790		impact on swimming behaviour; accumulation in cuticle	[137]
**CeO_2**	microalgae	200–25000		5600–6200		growth inhibition	[138]

ENP	Organism	dose range investigated (μg.L^{-1})	safe–toxic dose threshold (μg.L^{-1})	criteria value (μg.L^{-1})	criteria type used other than EC_{50}	major toxicity	Ref.
**CeO_2**	alga Pseudokirchneriella subcapitata	2200–460000		47000–395800	ErC_{20}	growth rate reduction	[139]
**ZnO**	Leptocheirus plumulosus	500–2000 μg/g sediment		763 μg/g sediment		accumulation in tissue	[118]
**ZnO**	Daphnia magna	500–1000		990–1150		Concentration–dependent uptake	[131]
**ZnO**	Daphnia magna	1000				Body burden increase	[140]
**ZnO**	Peringia ulvae	20				solubility–dependent bioaccumulation	[141]
**ZnO**	Danio rerio	1000		1900–15500	LC_{50}	body burden increase	[142]
**ZnO**	Carassius au ratus	1000–10000				accumulation in the intestine, gills, and liver	[123]
**ZnO**	Cyprinus Carpio	50000				increase in Zn content in intestine, gills, and liver	[143]
**ZnO**	marine phytoplankton diatoms, chlorophytes, and prymnesiophytes	10–1000	223–1000			depressed growth rate at concentrations > 223–1000	[99]
**ZnO**	Larval Zebrafish	12–10000				alteration of cancer cell differentiation	[144]
**ZnO**	zebrafish	1000–100000				neurotoxicity	[145]
**ZnO**	Diatoms	10000–80000				dose–dependent growth inhibition	[146]
**ZnO**	algae Dunaliella tertiolecta	100–10000		780–2310		growth rate inhibition	[147]
**ZnO**	microalga Chlorella vulgaris:	10000–50000				reduced growth rate; cell damage	[148]

ENP	Organism	dose range investigated (µg.L^{-1})	safe–toxic dose threshold (µg.L^{-1})	criteria value (µg.L^{-1})	criteria type used other than EC_{50}	major toxicity	Ref.
**ZnO**	microalgae Pseudokirchneriella subcapitata	50–2000		40–70		growth inhibition	[59]
**ZnO**	Daphnia magna	2500–20000	1000–5000	1100–6700		Immobilization	[108]
**ZnO**	Anabaena sp.	100–2000		740–1150		growth inhibition	[149]
**ZnO**	marine diatoms; crustaceans; medaka fish	4000–40000		680–4560	LC_{50} or IC_{50}	toxicity attributed to dissolved ions	[150]
**ZnO**	Daphnia magna; Thamnocephalus platyurus			180–3200	LC_{50}	extracellular ROS damaging cell membranes	[129]
**ZnO**	Daphnia magna	10000–100000		1000–100000		impacts on mortality and reproduction	[111]
**ZnO**	Daphnia magna			622–1511	EC_{50} and LC_{50}	immobilization and mortality	[112]
**ZnO**	Daphnia magna; Thamnocephalus platyurus			1100–16000	EC_{50} or LC_{50}	toxicity induced by dissolved metal ions	[130]
**ZnO**	Corophium volutator	200–1000		200–1000		delayed growth; impact on reproduction	[151]
**ZnO**	mussels Mytilus galloprovincialis	1000–10000				accumulation in tissue	[135]
**ZnO**	fish, Carassius auratus	10–100				gill hyperplasia and liver degeneration	[152]
**ZnO**	Artemia cysts hatching	100–100000		259340–259340	LC_{50}	mortality due to NPs clogging the gut; hatched cysts show increased oxidative stress	[114]
**ZnO**	mussel cells	1–100000		11554–40476	LC_{50}	ionic forms of the metals were more toxic than the nano forms	[153]

ENP	Organism	dose range investigated (μg.L^{-1})	safe–toxic dose threshold (μg.L^{-1})	criteria value (μg.L^{-1})	criteria type used other than EC_{50}	major toxicity	Ref.
**ZnO**	D. tertiolecta (Chlorophyceae: Chlamydomonadales)	5000–1000000		200–7500	EC_{50}	Zn ions release causes Zn accumulation in the cell and thus DNA damages	[115]
**ZnO**	*zebrafish embryos*	10–10000		4289–5538	LC_{50}	ionic forms of the metals were always more toxic than the nano forms	[154]
**Al_2O_3**	Ceriodaphnia dubia	20000–120000				concentration–dependent up take	[155]
**Al_2O_3**	fish, Carassius auratus	10–100				gill hyperplasia and liver degeneration	[152]
**Al_2O_3**	Daphnia magna			114357–162392	EC_{50} and LC_{50}	immobilization and mortality	[112]
**Al_2O_3**	Artemia salina (crustacean filter feeders) larvae	5000–100000		100000	LC_{50}	marginal acute toxicity; toxic effects are mediated by oxidative stress; smaller NPs exhibited a more toxic effect than larger particles	[156]
**Al_2O_3**	green algae Porphyridium aerugineum Geitler	1000–1000000		100000–300000	EC_{50}	shading effects of NP accumulation on the surface of algae could inhibit the photosynthetic activity	[64]
**Fe_2O_3**	Danio rerio	4000–10000				Increased Fe content in tissue; 86% –100% depuration after 24 days	[157]
**Fe_2O_3**	tilapia (Oreochromis niloticus)	100–1000				rapid accumulation in organs and slow elimination	[158]
**Fe_2O_3**	Zebrafish (Danio rerio)	100–100000		36060–53350		delay in embryo hatching; eventually mortality	[159]
**Fe_2O_3**	carp: Labeo rohita	500000				adverse impacts on haematology and gill activity	[160]

ENP	Organism	dose range investigated (μg.L^{-1})	safe–toxic dose threshold (μg.L^{-1})	criteria value (μg.L^{-1})	criteria type used other than EC_{50}	major toxicity	Ref.
**Fe_2O_3**	Aquatic plant: Lemna minor	30000–50000				lipid peroxidation increase with ENP concentration; accumulation in root	[161]
**Fe_2O_3**	Daphnia magna	1250–40000		3540		lethality on embryo	[162]
**Fe_2O_3**	green alga (Chlorella pyrenoidosa)	NA		71000–132000	IC_{50}	oxidative stress	[163]
**Fe_3O_4**	rainbow trout (Oncorhynchus mykiss)	50000–800000		285940–494090		damaging effect on antioxidant system at concentrations >285940	[164]
**Fe_3O_4**	rotifer	10000–500000		722000–1000000		acute toxicity	[165]
**Fe_3O_4**	brine shrimp Artemia salina	10000–1e6				accumulated in gut; no significant mortality	[133]
**Fe_3O_4**	invertebrate zooplankton: Artemia salina cysts and larvae	25000–600000		365000–>600000		Disruption of Mitochondrial morphology	[166]
**Fe_3O_4**	zebrafish	100000				oxidative stress, DNA damage, and apoptosis in liver	[167]
**Fe_3O_4**	zebrafish (Danio rerio)	4000–10000				Increased Fe content in tissue; 86% –100% depuration after 24 days	[157]
**Fe_3O_4**	Ragworm (Hediste diversicolor); Bivalve mollusc (Scrobicularia plana)	10				protein degradation; morphological impairment; bioaccumulation	[128]
**Fe_3O_4**	green alga (Chlorella pyrenoidosa)	NA		33000	IC_{50}	oxidative stress	[163]

ENP	Organism	dose range investigated (µg.L^{-1})	safe–toxic dose threshold (µg.L^{-1})	criteria value (µg.L^{-1})	criteria type used other than EC_{50}	major toxicity	Ref.
**Fe_3O_4**	Sea urchin (Paracentrotus lividus)	100–10000				behavioural impairments; oxidative stress increase	134
**Fe_3O_4**	*D. magna*	5000–500000		654650–4965920	LC_{50}	dose–dependent toxicity	168
**NZVI**	Chlamydomonas reinhardtii	1800–180000				High concentrations caused delayed growth	51
**NZVI**	mussel: Mytilus galloprovincialis	100–10000				30% mortality, significant DNA damage at highest NZVI concentration	169
**NZVI**	phytoplankton and zooplankton (Daphnia magna)	200–100000	300–3000			decrease in growth rates; increasing mortality	170
**NZVI**	green alga (Chlorella pyrenoidosa)	NA		19800–913000	IC_{50}	oxidative stress	163
**SiO_2**	mussel hemocytes (Mytilus galloprovincialis)	1–10				Extracellular oxyradical nitric oxide production	171
**SiO_2**	mussel (Mytilus galloprovincialis)	50–5000				hemocytes and digestive glands	105
**SiO_2**	mussel (Mytilus edulis)					Uptake in gills and hepatopancreas; toxic cell injury	172
**SiO_2 and SiO_2 coated with alumina**	P. subcapitata (Korshikov) Hindak	4600–1000000		10000–1206900	ErC_{50}	bare SiO2 NPs were more toxic than alumina coated SiO2 NPs	173
**SiO_2**	D. tertiolecta (Chlorophyceae: Chlamydomonadales)	125000–2000000		49000–57000	EC_{50}	toxicity starting from oxidative stress generation; ROS production the main cause of growth inhibition	115

ENP	Organism	dose range investigated (µg.L^{-1})	safe–toxic dose threshold (µg.L^{-1})	criteria value (µg.L^{-1})	criteria type used other than EC_{50}	major toxicity	Ref.
**SiO_2 nanostructures**	*D. magna*	780–10000000		220000–7880000	EC_{50}	toxicity was influenced by the surface modifications; Toxicity observed as alterations in the microvilli and mitochondria of the D. magna intestine and some damage to egg cells	[174]
**SiO_2**	*D. magna*	25000–400000		148871–660943	EC_{50} and LC_{50}	dose–dependent toxicity	[175]
**SiO_2**	*D. magna*	500–100000		1730–3704000	LC_{50}	dose–dependent toxicity	[168]
**SiO_2**	*zebrafish embryos*	100–100000		83329–100000	LC_{50}	ionic forms of the metals were always more toxic than the nano forms	[154]
**SiO_2**	mussel cells	1–100000		100000–250000	LC_{50}	ionic forms of the metals were more toxic than the nano forms	[153]
**SiO_2**	*Dunaliella salina green algae*	100–50000		169–851	EC_{50}	reduced growth with an increase of NP concentration and exposure time	[176]
**SiO_2**	Artemia cysts hatching	10–100000		23590–23590	LC_{50}	mortality due to NPs clogging the gut; hatched cysts show increased oxidative stress	[114]
**SiO_2**	*Scenedesmus obliquus*	200000–1600000		144000–388000	EC_{20}	toxicity NPs probably due to their attachment to algal cells surface	[177]
**SiO_2**	marine microalgae Dunaliella tertiolecta	5000–200000		19380–25430	EC_{50}	NP direct action upon membrane integrity	[116]
**Silica–coated iron oxide**	cells of Chinook salmon, Oncorhynchus tshawytscha embryos	10–30				cytotoxicity in cells	[178]

ENP	Organism	dose range investigated (μg.L^{-1})	safe–toxic dose threshold (μg.L^{-1})	criteria value (μg.L^{-1})	criteria type used other than EC_{50}	major toxicity	Ref.
**CdS and CdSe QDs**	snail Peringia ulvae					bioaccumulation	179
**Carboxyl QD**	algae Pseudokirchneriella subcapitata and Ceriodaphnia dubia	11–110	110	37.1–>110	LC_{50}	food chain transfer	180
**CdSe/ZnS QD**	amphipod Leptocheirus plumulosus	11.3–543		11.6–56.1	LC_{50}	trophic transfer; bioavailability	181
**CdS/ZnS QD**	human cell	956.9–19138		4402–17416	IC_{50}	cell death	182
**CdSecore/ZnSshell QDs**	Zebrafish Embryo	38.3–3827.7		1340–8038	LC_{50}	Cadmium accumulation	183
**CdSe/ZnS QDs**	Daphnia magna	–2000		244–2000	LC_{50}	accumulation within the digestive tracts	184
**CdTe QD**	green alga Chlamydomonas reinhartii	1–100000	1000	5000		growth inhibition; oxidative stress	113
**Carbon QD**	zebrafish (Danio rerio), zooplankton (Daphnia magna), and phytoplankton (Scenedesmus obliquus)	5000–200000		74800–232500		mortality and immobility; photosynthesis and nutrition absorption inhibition in a dose– and time–dependent manner	185
**SeCd/ZnS QD**	zebrafish larvae	500–1000				bioaccumulation	186
**polymer–coated CdSe/ZnS QD**	Daphnia magna	–2000		110–3840		dose–dependent mortality	187
**HAp**	catfish cells and zebrafish embryos	10–300				no cell viability effect; lower metabolic activity of cells; axial	188

**ENP**	**Organism**	**dose range investigated (μg.L^{-1})**	**safe–toxic dose threshold (μg.L^{-1})**	**criteria value (μg.L^{-1})**	**criteria type used other than EC_{50}**	**major toxicity**	**Ref.**
						deformation and hatching delay in zebrafish	
**ENP**	Organism	dose range investigated (μg.L^{-1})	safe–toxic dose threshold (μg.L^{-1})	criteria value (μg.L^{-1})	criteria type used other than EC_{50}	major toxicity	Ref.
**TiO_2**	zebra mussels, Dreissena polymorpha	7–830				increasing Ti tissue concentration	94
**TiO_2**	Daphnia magna	45000				Highest uptake for aggregated ENPs	95
**TiO_2**	Daphnia magna	25000				larger TiO_2 ENPs were taken up faster	96
**TiO_2**	Ceriodaphnia dubia	1000–64000		>64000		concentrM10 dependent up take	97
**TiO_2**	Daphnia magna	1000–10000		5500–>500000		mortality	98
**TiO_2**	marine phytoplanktons diatoms, chlorophytes, and prymnesiophytes	10–1000				no effect on growth rate	99
**TiO_2**	zebrafish: Danio rerio	10–250				alteration of lipid accumulation and ROS in embryos	100
**TiO_2**	zebrafish larvae	100–10000				Accumulation in brain, cell death, doe–dependent toxicity	101
**TiO_2**	zebrafish (Danio rerio)	1000–4000				negatively affected spermatogenic cells and testicular morphology at high dozes	102

ENP	Organism	dose range investigated (μg.L^{-1})	safe–toxic dose threshold (μg.L^{-1})	criteria value (μg.L^{-1})	criteria type used other than EC_{50}	major toxicity	Ref.
**TiO_2**	Cyanobacteria: Anabaena variabilis	500–250000		420–620		reduced growth rate; dose–dependent effect	103
**TiO_2**	Lugworm (Arenicola marina)	1–3 g/kg				DNA damage	104
**TiO_2**	mussel (Mytilus galloprovincialis)	50–5000				digestive glands	105
**TiO_2**	abalone (Haliotis diversicolor supertexta)	100–10000	1000	>10000		not acutely toxic; Oxidative stress	106
**TiO_2**	abalone (Haliotis diversicolor supertexta) Embryos	2000–250000	10	56900–345800		hatching inhibition and malformations	107
**TiO_2**	Daphnia magna	12500–100000	100000	>100000		Immobilization	108
**TiO_2**	Microlalgae: Chlorella sp.	0–1000000		4900–48000	IC_{50}	growth inhibition	109
**TiO_2**	Daphnia magna	100–100000		460–100000		immobilization; mortality; and high level of bioaccumulation	110
**TiO_2**	Daphnia magna			>100000	EC_{50}	impacts on mortality and reproduction	111
**TiO_2**	Daphnia magna			35306–143387	EC_{50} and LC_{50}	immobilization and mortality	112
**TiO_2**	microalgae Pseudokirchneriella subcapitata	50–2000		2500–241000		growth inhibition	59
**TiO_2**	green alga Chlamydomonas reinhartii	1–100000	1000	–10000		growth inhibition; oxidative stress	113

ENP	Organism	dose range investigated (µg.L^{-1})	safe–toxic dose threshold (µg.L^{-1})	criteria value (µg.L^{-1})	criteria type used other than EC_{50}	major toxicity	Ref.
**TiO_2**	Artemia cysts hatching	10–100000		18940	LC_{50}	mortality due to NPs clogging the gut; hatched cysts show increased oxidative stress	[114]
**TiO_2**	D. tertiolecta (Chlorophyceae: Chlamydomonadales)	125000–1000000		20000–95000	EC_{50}	toxicity starting from oxidative stress generation; ROS production the main cause of growth inhibition	[115]
**TiO_2**	marine microalgae Dunaliella tertiolecta	1000–100000		186220–188880	EC_{50}	toxic action due to cell entrapment and agglomeration	[116]
**CuO**	Macoma balthica	200				Body burden increase	[117]
**CuO**	Leptocheirus plumulosus	500–2000 ug/g sediment		8.6 µg/g sediment		Body burden increase	[118]
**Cu**	Daphnia magna	NA		40–1130	LC_{50}	concentration dependent uptake; mortality	[119]
**CuO**	Lymnaea stag nalis	0.3–2466				bioaccumulation	[120]
**CuO**	Potamopyrgus antipodarum	240 µg/g sediments				higher body burden	[121]
**CuO**	Potamopyrgus antipodarum	240 µg/g sediments				Increased Cu content in tissue	[122]
**CuO**	zooplankton (Artemia salina) and goldfish (Carassius auratus)	1000–10000				Accumulation in intestine, gills, and liver	[123]
**Cu**	zebrafish embryos	150–1000				developmental defects during zebrafish embryogenesis	[124]
**CuO**	zebrafish	8–15910	12700	>15910		Embryo mortality and hatching decrease; no toxicity to cells	[125]
**Cu**	zebrafish	250				down–regulating Wnt signalling	[126]

ENP	Organism	dose range investigated (μg.L^{-1})	safe–toxic dose threshold (μg.L^{-1})	criteria value (μg.L^{-1})	criteria type used other than EC_{50}	major toxicity	Ref.
**CuO**	mussel: Mytilus edulis	400000–1000000				impact on lysosomal membrane stability	127
**CuO**	Ragworm (Hediste diversicolor); Bivalve mollusc (Scrobicularia plana)					Cholinesterase activity reduction	128
**CuO**	Daphnia magna; Thamnocephalus platyurus			2100–3200		extracellular ROS damaging cell membranes	129
**CuO**	Daphnia magna; Thamnocephalus platyurus			90000–224000	EC_{50} or LC_{50}	toxicity induced by dissolved metal ions	130
**CuO**	Artemia cysts hatching	100–100000		20920	LC_{50}	mortality due to NPs clogging the gut; hatched cysts show increased oxidative stress	114
**Cu**	Daphnia magna	500–1000		30–93	LC_{50}	Concentration dependent uptake	131
**CeO_2**	Drissena poly morpha	10–100				Body burden increase	132
**CeO_2**	brine shrimp Artemia salina	10000–1e6				accumulated in gut; no significant mortality	133
**CeO_2**	Sea urchin (Paracentrotus lividus)	100–10000				behavioral impairments; oxidative stress increase	134
**CeO_2**	mussels Mytilus galloprovincialis	1000–10000				accumulating only a small fraction in tissues	135
**CeO_2**	Daphnia pulex					40–100% uptake	136
**CeO_2**	Daphnia similis and Daphnia pulex	1000–100000		260–91790		impact on swimming behaviour; accumulation in cuticle	137
**CeO_2**	microalgae	200–25000		5600–6200		growth inhibition	138

ENP	Organism	dose range investigated (μg.L^{-1})	safe–toxic dose threshold (μg.L^{-1})	criteria value (μg.L^{-1})	criteria type used other than EC_{50}	major toxicity	Ref.
**CeO_2**	alga Pseudokirchneriella subcapitata	2200–460000		47000–395800	ErC_{20}	growth rate reduction	[139]
**ZnO**	Leptocheirus plumulosus	500–2000 μg/g sediment		763 μg/g sediment		accumulation in tissue	[118]
**ZnO**	Daphnia magna	500–1000		990–1150		Concentration–dependent uptake	[131]
**ZnO**	Daphnia magna	1000				Body burden increase	[140]
**ZnO**	Peringia ulvae	20				solubility–dependent bioaccumulation	[141]
**ZnO**	Danio rerio	1000		1900–15500	LC_{50}	body burden increase	[142]
**ZnO**	Carassius au ratus	1000–10000				accumulation in the intestine, gills, and liver	[123]
**ZnO**	Cyprinus Carpio	50000				increase in Zn content in intestine, gills, and liver	[143]
**ZnO**	marine phytoplankton diatoms, chlorophytes, and prymnesiophytes	10–1000	223–1000			depressed growth rate at concentrations > 223–1000	[99]
**ZnO**	Larval Zebrafish	12–10000				alteration of cancer cell differentiation	[144]
**ZnO**	zebrafish	1000–100000				neurotoxicity	[145]
**ZnO**	Diatoms	10000–80000				dose–dependent growth inhibition	[146]
**ZnO**	algae Dunaliella tertiolecta	100–10000		780–2310		growth rate inhibition	[147]
**ZnO**	microalga Chlorella vulgaris:	10000–50000				reduced growth rate; cell damage	[148]

ENP	Organism	dose range investigated (µg.L^{-1})	safe–toxic dose threshold (µg.L^{-1})	criteria value (µg.L^{-1})	criteria type used other than EC_{50}	major toxicity	Ref.
**ZnO**	microalgae Pseudokirchneriella subcapitata	50–2000		40–70		growth inhibition	[59]
**ZnO**	Daphnia magna	2500–20000	1000–5000	1100–6700		Immobilization	[108]
**ZnO**	Anabaena sp.	100–2000		740–1150		growth inhibition	[149]
**ZnO**	marine diatoms; crustaceans; medaka fish	4000–40000		680–4560	LC_{50} or IC_{50}	toxicity attributed to dissolved ions	[150]
**ZnO**	Daphnia magna; Thamnocephalus platyurus			180–3200	LC_{50}	extracellular ROS damaging cell membranes	[129]
**ZnO**	Daphnia magna	10000–100000		1000–100000		impacts on mortality and reproduction	[111]
**ZnO**	Daphnia magna			622–1511	EC_{50} and LC_{50}	immobilization and mortality	[112]
**ZnO**	Daphnia magna; Thamnocephalus platyurus			1100–16000	EC_{50} or LC_{50}	toxicity induced by dissolved metal ions	[130]
**ZnO**	Corophium volutator	200–1000		200–1000		delayed growth; impact on reproduction	[151]
**ZnO**	mussels Mytilus galloprovincialis	1000–10000				accumulation in tissue	[135]
**ZnO**	fish, Carassius auratus	10–100				gill hyperplasia and liver degeneration	[152]
**ZnO**	Artemia cysts hatching	100–100000		259340–259340	LC_{50}	mortality due to NPs clogging the gut; hatched cysts show increased oxidative stress	[114]
**ZnO**	mussel cells	1–100000		11554–40476	LC_{50}	ionic forms of the metals were more toxic than the nano forms	[153]

ENP	Organism	dose range investigated (μg.L^{-1})	safe–toxic dose threshold (μg.L^{-1})	criteria value (μg.L^{-1})	criteria type used other than EC_{50}	major toxicity	Ref.
**ZnO**	D. tertiolecta (Chlorophyceae: Chlamydomonadales)	5000–1000000		200–7500	EC_{50}	Zn ions release causes Zn accumulation in the cell and thus DNA damages	[115]
**ZnO**	*zebrafish embryos*	10–10000		4289–5538	LC_{50}	ionic forms of the metals were always more toxic than the nano forms	[154]
**Al_2O_3**	Ceriodaphnia dubia	20000–120000				concentration–dependent up take	[155]
**Al_2O_3**	fish, Carassius auratus	10–100				gill hyperplasia and liver degeneration	[152]
**Al_2O_3**	Daphnia magna			114357–162392	EC_{50} and LC_{50}	immobilization and mortality	[112]
**Al_2O_3**	Artemia salina (crustacean filter feeders) larvae	5000–100000		100000	LC_{50}	marginal acute toxicity; toxic effects are mediated by oxidative stress; smaller NPs exhibited a more toxic effect than larger particles	[156]
**Al_2O_3**	green algae Porphyridium aerugineum Geitler	1000–1000000		100000–300000	EC_{50}	shading effects of NP accumulation on the surface of algae could inhibit the photosynthetic activity	[64]
**Fe_2O_3**	Danio rerio	4000–10000				Increased Fe content in tissue; 86% –100% depuration after 24 days	[157]
**Fe_2O_3**	tilapia (Oreochromis niloticus)	100–1000				rapid accumulation in organs and slow elimination	[158]
**Fe_2O_3**	Zebrafish (Danio rerio)	100–100000		36060–53350		delay in embryo hatching; eventually mortality	[159]
**Fe_2O_3**	carp: Labeo rohita	500000				adverse impacts on haematology and gill activity	[160]

ENP	Organism	dose range investigated (μg.L^{-1})	safe–toxic dose threshold (μg.L^{-1})	criteria value (μg.L^{-1})	criteria type used other than EC_{50}	major toxicity	Ref.
**Fe_2O_3**	Aquatic plant: Lemna minor	30000–50000				lipid peroxidation increase with ENP concentration; accumulation in root	[161]
**Fe_2O_3**	Daphnia magna	1250–40000		3540		lethality on embryo	[162]
**Fe_2O_3**	green alga (Chlorella pyrenoidosa)	NA		71000–132000	IC_{50}	oxidative stress	[163]
**Fe_3O_4**	rainbow trout (Oncorhynchus mykiss)	50000–800000		285940–494090		damaging effect on antioxidant system at concentrations >285940	[164]
**Fe_3O_4**	rotifer	10000–500000		722000–1000000		acute toxicity	[165]
**Fe_3O_4**	brine shrimp Artemia salina	10000–1e6				accumulated in gut; no significant mortality	[133]
**Fe_3O_4**	invertebrate zooplankton: Artemia salina cysts and larvae	25000–600000		365000–>600000		Disruption of Mitochondrial morphology	[166]
**Fe_3O_4**	zebrafish	100000				oxidative stress, DNA damage, and apoptosis in liver	[167]
**Fe_3O_4**	zebrafish (Danio rerio)	4000–10000				Increased Fe content in tissue; 86% –100% depuration after 24 days	[157]
**Fe_3O_4**	Ragworm (Hediste diversicolor); Bivalve mollusc (Scrobicularia plana)	10				protein degradation; morphological impairment; bioaccumulation	[128]
**Fe_3O_4**	green alga (Chlorella pyrenoidosa)	NA		33000	IC_{50}	oxidative stress	[163]

ENP	Organism	dose range investigated (μg.L^{-1})	safe–toxic dose threshold (μg.L^{-1})	criteria value (μg.L^{-1})	criteria type used other than EC_{50}	major toxicity	Ref.
**Fe_3O_4**	Sea urchin (Paracentrotus lividus)	100–10000				behavioural impairments; oxidative stress increase	[134]
**Fe_3O_4**	*D. magna*	5000–500000		654650–4965920	LC_{50}	dose–dependent toxicity	[168]
**NZVI**	Chlamydomonas reinhardtii	1800–180000				High concentrations caused delayed growth	[51]
**NZVI**	mussel: Mytilus galloprovincialis	100–10000				30% mortality, significant DNA damage at highest NZVI concentration	[169]
**NZVI**	phytoplankton and zooplankton (Daphnia magna)	200–100000	300–3000			decrease in growth rates; increasing mortality	[170]
**NZVI**	green alga (Chlorella pyrenoidosa)	NA		19800–913000	IC_{50}	oxidative stress	[163]
**SiO_2**	mussel hemocytes (Mytilus galloprovincialis)	1–10				Extracellular oxyradical nitric oxide production	[171]
**SiO_2**	mussel (Mytilus galloprovincialis)	50–5000				hemocytes and digestive glands	[105]
**SiO_2**	mussel (Mytilus edulis)					Uptake in gills and hepatopancreas; toxic cell injury	[172]
**SiO_2 and SiO_2 coated with alumina**	P. subcapitata (Korshikov) Hindak	4600–1000000		10000–1206900	ErC_{50}	bare SiO2 NPs were more toxic than alumina coated SiO2 NPs	[173]
**SiO_2**	D. tertiolecta (Chlorophyceae: Chlamydomonadales)	125000–2000000		49000–57000	EC_{50}	toxicity starting from oxidative stress generation; ROS production the main cause of growth inhibition	[115]

ENP	Organism	dose range investigated (μg.L^{-1})	safe–toxic dose threshold (μg.L^{-1})	criteria value (μg.L^{-1})	criteria type used other than EC_{50}	major toxicity	Ref.
**SiO_2 nanostructures**	*D. magna*	780–10000000		220000–7880000	EC_{50}	toxicity was influenced by the surface modifications; Toxicity observed as alterations in the microvilli and mitochondria of the D. magna intestine and some damage to egg cells	[174]
**SiO_2**	*D. magna*	25000–400000		148871–660943	EC_{50} and LC_{50}	dose–dependent toxicity	[175]
**SiO_2**	*D. magna*	500–100000		1730–3704000	LC_{50}	dose–dependent toxicity	[168]
**SiO_2**	*zebrafish embryos*	100–100000		83329–100000	LC_{50}	ionic forms of the metals were always more toxic than the nano forms	[154]
**SiO_2**	mussel cells	1–100000		100000–250000	LC_{50}	ionic forms of the metals were more toxic than the nano forms	[153]
**SiO_2**	*Dunaliella salina green algae*	100–50000		169–851	EC_{50}	reduced growth with an increase of NP concentration and exposure time	[176]
**SiO_2**	Artemia cysts hatching	10–100000		23590–23590	LC_{50}	mortality due to NPs clogging the gut; hatched cysts show increased oxidative stress	[114]
**SiO_2**	*Scenedesmus obliquus*	200000–1600000		144000–388000	EC_{20}	toxicity NPs probably due to their attachment to algal cells surface	[177]
**SiO_2**	marine microalgae Dunaliella tertiolecta	5000–200000		19380–25430	EC_{50}	NP direct action upon membrane integrity	[116]
**Silica–coated iron oxide**	cells of Chinook salmon, Oncorhynchus tshawytscha embryos	10–30				cytotoxicity in cells	[178]

ENP	Organism	dose range investigated (μg.L^{-1})	safe–toxic dose threshold (μg.L^{-1})	criteria value (μg.L^{-1})	criteria type used other than EC_{50}	major toxicity	Ref.
**CdS and CdSe QDs**	snail Peringia ulvae					bioaccumulation	179
**Carboxyl QD**	algae Pseudokirchneriella subcapitata and Ceriodaphnia dubia	11–110	110	37.1–>110	LC_{50}	food chain transfer	180
**CdSe/ZnS QD**	amphipod Leptocheirus plumulosus	11.3–543		11.6–56.1	LC_{50}	trophic transfer; bioavailability	181
**CdS/ZnS QD**	human cell	956.9–19138		4402–17416	IC_{50}	cell death	182
**CdSecore/ZnSshell QDs**	Zebrafish Embryo	38.3–3827.7		1340–8038	LC_{50}	Cadmium accumulation	183
**CdSe/ZnS QDs**	Daphnia magna	2000		244–2000	LC_{50}	accumulation within the digestive tracts	184
**CdTe QD**	green alga Chlamydomonas reinhartii	1–100000	1000	5000		growth inhibition; oxidative stress	113
**Carbon QD**	zebrafish (Danio rerio), zooplankton (Daphnia magna), and phytoplankton (Scenedesmus obliquus)	5000–200000		74800–232500		mortality and immobility; photosynthesis and nutrition absorption inhibition in a dose– and time–dependent manner	185
**SeCd/ZnS QD**	zebrafish larvae	500–1000				bioaccumulation	186
**polymer–coated CdSe/ZnS QD**	Daphnia magna	2000		110–3840		dose–dependent mortality	187
**HAp**	catfish cells and zebrafish embryos	10–300				no cell viability effect; lower metabolic activity of cells; axial	188

ENP	Organism	dose range investigated (µg.L^{-1})	safe–toxic dose threshold (µg.L^{-1})	criteria value (µg.L^{-1})	criteria type used other than EC_{50}	major toxicity	Ref.
						deformation and hatching delay in zebrafish	

References

1 Pati, P., McGinnis, S. & Vikesland, P. J. Life cycle assessment of "green" nanoparticle synthesis methods. *Environmental Engineering Science* **31**, 410-420 (2014).

2 Frischknecht, R. *et al.* The ecoinvent database: overview and methodological framework (7 pp). *The international journal of life cycle assessment* **10**, 3-9 (2005).

3 Sun, Q., Feitz, A. J., Guan, J. & Waite, T. D. Comparison of the reactivity of nanosized zero-valent iron (nZVI) particles produced by borohydride and dithionite reduction of iron salts. *Nano* **3**, 341-349 (2008).

4 Kantürk, A. & Pişkin, S. Innovation in sodium borohydride production process from borosilicate glass with sodium under hydrogen atmosphere:"high pressure process". *International journal of hydrogen energy* **32**, 3981-3986 (2007).

5 Visentin, C., da Silva Trentin, A. W., Braun, A. B. & Thomé, A. Lifecycle assessment of environmental and economic impacts of nano-iron synthesis process for application in contaminated site remediation. *Journal of cleaner production* **231**, 307-319 (2019).

6 Kozma, G., Rónavári, A., Kónya, Z. & Kukovecz, A. Environmentally benign synthesis methods of zero-valent iron nanoparticles. *ACS Sustainable Chemistry & Engineering* **4**, 291-297 (2016).

7 Li, S., Yan, W. & Zhang, W.-x. Solvent-free production of nanoscale zero-valent iron (nZVI) with precision milling. *Green Chemistry* **11**, 1618-1626 (2009).

8 Seyedi, S. M., Rabiee, H., Shahabadi, S. M. S. & Borghei, S. M. Synthesis of Zero-Valent Iron Nanoparticles Via Electrical Wire Explosion for Efficient Removal of Heavy Metals. *CLEAN–Soil, Air, Water* **45**, 1600139 (2017).

9 Salavati-Niasari, M., Javidi, J. & Dadkhah, M. Ball milling synthesis of silica nanoparticle from rice husk ash for drug delivery application. *Combinatorial chemistry & high throughput screening* **16**, 458-462 (2013).

10 Buazar, F. Impact of biocompatible nanosilica on green stabilization of subgrade soil. *Scientific reports* **9**, 1-9 (2019).

11 Akl, M. A., Aly, H. F., Soliman, H. M. A., Aref, M. E. & ElRahman, A. Preparation and characterization of silica nanoparticles by wet mechanical attrition of white and yellow sand. *J Nanomed Nanotechnol* **4**, 2 (2013).

12 Koopi, H. & Buazar, F. A novel one-pot biosynthesis of pure alpha aluminum oxide nanoparticles using the macroalgae Sargassum ilicifolium: A green marine approach. *Ceramics International* **44**, 8940-8945 (2018).

13 André, B., Coulet, M. V., Esposito, P. H., Rufino, B. & Denoyel, R. High-energy ball milling to enhance the reactivity of aluminum nanopowders. *Materials Letters* **110**, 108-110 (2013).

14 Salah, N. *et al.* High-energy ball milling technique for ZnO nanoparticles as antibacterial material. *International journal of nanomedicine* **6**, 863 (2011).

15 Yadav, T. P. & Srivastava, O. N. Synthesis of nanocrystalline cerium oxide by high energy ball milling. *Ceramics International* **38**, 5783-5789 (2012).

16 Goedkoop, M., De Schryver, A., Oele, M., Durksz, S. & de Roest, D. Introduction to LCA with SimaPro 7. *PRé Consultants, The Netherlands* (2008).

17 Harrison, D. P. A method for estimating the cost to sequester carbon dioxide by delivering iron to the ocean. *International Journal of Global Warming* **5**, 231-254 (2013).

18 Banu, J. R., Kavitha, S., Gunasekaran, M. & Kumar, G. Microalgae based biorefinery promoting circular bioeconomy-techno economic and life-cycle analysis. *Bioresource technology* **302**, 122822 (2020).

19 Davis, R. *et al.* Process design and economics for the conversion of algal biomass to biofuels: algal biomass fractionation to lipid-and carbohydrate-derived fuel products. (National Renewable Energy Lab.(NREL), Golden, CO (United States), 2014).

20 https://www.netsuite.co.uk/.

21 https://fundsquire.co.uk/how-much-does-payroll-cost-companies-percentage-by-industry/.

22 https://www.ons.gov.uk/economy/economicoutputandproductivity/productivitymeasures/bulletins/labourcostsandlabourincomeuk/2021.

23 Coale, K. H. *et al.* IronEx-I, an in situ iron-enrichment experiment: Experimental design, implementation and results. *Deep Sea Research Part II: Topical Studies in Oceanography* **45**, 919-945 (1998).

24 Kadar, E., Rooks, P., Lakey, C. & White, D. A. The effect of engineered iron nanoparticles on growth and metabolic status of marine microalgae cultures. *Science of the total environment* **439**, 8-17 (2012).

25 https://www.airag.com.au/reducing-aerial-spray-costs.

26 https://www.agaviation.org/Files/asabe/2016/dharmasena-martin-naaa-2016-presentation.pdf.

27 https://www.freightos.com/freight-resources/ocean-freight-explained/

28 www.searates.com.

29 https://www.dsv.com/en/our-solutions/modes-of-transport/sea-freight/shipping-container-dimensions/dry-container.

30 https://www.chinaimportal.com/blog/shipping-costs-when-importing-from-china-a-complete-guide/.

31 https://www.statista.com/statistics/233886/minimum-wage-per-hour-in-china-by-city-and-province/.

32 Piccinno, F., Hischier, R., Seeger, S. & Som, C. From laboratory to industrial scale: a scale-up framework for chemical processes in life cycle assessment studies. *Journal of Cleaner Production* **135**, 1085-1097 (2016).

33 Visentin, C., da Silva Trentin, A. W., Braun, A. B. & Thomé, A. Life cycle sustainability assessment of the nanoscale zero-valent iron synthesis process for application in contaminated site remediation. *Environmental Pollution* **268**, 115915 (2021).

34 Pourzahedi, L. & Eckelman, M. J. Comparative life cycle assessment of silver nanoparticle synthesis routes. *Environmental Science: Nano* **2**, 361-369 (2015).

35 Falinski, M. M. *et al.* A framework for sustainable nanomaterial selection and design based on performance, hazard, and economic considerations. *Nature nanotechnology* **13**, 708-714 (2018).

36 Marimón-Bolívar, W. & González, E. E. Green synthesis with enhanced magnetization and life cycle assessment of Fe3O4 nanoparticles. *Environmental Nanotechnology, Monitoring & Management* **9**, 58-66 (2018).

37 Slotte, M. & Zevenhoven, R. Energy requirements and life cycle assessment of production and product integration of silver, copper and zinc nanoparticles. *Journal of Cleaner Production* **148**, 948-957 (2017).

38 Meyer, D. E., Curran, M. A. & Gonzalez, M. A. (ACS Publications, 2009).

39 Feijoo, S. *et al.* Comparative life cycle assessment of different synthesis routes of magnetic nanoparticles. *Journal of Cleaner Production* **143**, 528-538 (2017).

40 de Baar, H. J. W., Gerringa, L. J. A., Laan, P. & Timmermans, K. R. Efficiency of carbon removal per added iron in ocean iron fertilization. *Marine Ecology Progress Series* **364**, 269-282 (2008).

41 Strong, A. L., Cullen, J. J. & Chisholm, S. W. Ocean fertilization: Science, policy, and commerce. *Oceanography* **22**, 236-261 (2009).

42 Acevedo-Trejos, E., Brandt, G., Bruggeman, J. & Merico, A. Mechanisms shaping size structure and functional diversity of phytoplankton communities in the ocean. *Scientific reports* **5**, 1-8 (2015).

43 https://www.theworldcounts.com/challenges/climate-change/global-warming/global-co2-emissions/story.

44 https://www.statista.com. https://www.statista.com/statistics/267380/iron-ore-mine-production-by-country/.

45 IPCC. *Mitigation of Climate Change, Working Group III contribution to the Sixth Assessment Report of the Intergovernmental Panel on Climate Change*. (2022).

46 Graca, B., Zgrundo, A., Zakrzewska, D., Rzodkiewicz, M. & Karczewski, J. Origin and fate of nanoparticles in marine water–Preliminary results. *Chemosphere* **206**, 359-368 (2018).

47 Wells, M. L. & Goldberg, E. D. Occurrence of small colloids in sea water. *Nature* **353**, 342-344 (1991).

48 https://www.epa.gov/energy/greenhouse-gases-equivalencies-calculator-calculations-and-references.

49 Pádrová, K. *et al.* Trace concentrations of iron nanoparticles cause overproduction of biomass and lipids during cultivation of cyanobacteria and microalgae. *Journal of Applied Phycology* **27**, 1443-1451, doi:10.1007/s10811-014-0477-1 (2014).

50 Kadar, E., Rooks, P., Lakey, C. & White, D. A. The effect of engineered iron nanoparticles on growth and metabolic status of marine microalgae cultures. *The Science of the total environment* **439**, 8-17, doi:10.1016/j.scitotenv.2012.09.010 (2012).

51 Adeleye, A. S. *et al.* Influence of Phytoplankton on Fate and Effects of Modified Zerovalent Iron Nanoparticles. *Environmental science & technology* **50**, 5597-5605, doi:10.1021/acs.est.5b06251 (2016).

52 He, M. *et al.* Improvement on lipid production by Scenedesmus obliquus triggered by low dose exposure to nanoparticles. *Scientific reports* **7**, 15526, doi:10.1038/s41598-017-15667-0 (2017).

53 Seo, J. Y. *et al.* Downstream integration of microalgae harvesting and cell disruption by means of cationic surfactant-decorated Fe3O4 nanoparticles. *Green Chemistry* **18**, 3981-3989, doi:10.1039/c6gc00904b (2016).

54 Hu, Y. R., Wang, F., Wang, S. K., Liu, C. Z. & Guo, C. Efficient harvesting of marine microalgae Nannochloropsis maritima using magnetic nanoparticles. *Bioresour Technol* **138**, 387-390, doi:10.1016/j.biortech.2013.04.016 (2013).

55 Kang, N. K. *et al.* Enhancing lipid productivity of Chlorella vulgaris using oxidative stress by TiO2 nanoparticles. *Korean Journal of Chemical Engineering* **31**, 861-867, doi:10.1007/s11814-013-0258-6 (2014).

56 Deng, X. Y. *et al.* Biological effects of TiO2 and CeO2 nanoparticles on the growth, photosynthetic activity, and cellular components of a marine diatom Phaeodactylum tricornutum. *The Science of the total environment* **575**, 87-96, doi:10.1016/j.scitotenv.2016.10.003 (2017).

57 Hazeem, L. J. *et al.* Cumulative effect of zinc oxide and titanium oxide nanoparticles on growth and chlorophyll a content of Picochlorum sp. *Environmental science and pollution research international* **23**, 2821-2830, doi:10.1007/s11356-015-5493-4 (2016).

58 Zahra, Z. *et al.* Phycobiliproteins Production Enhancement and Lipidomic Alteration by Titanium Dioxide Nanoparticles in Synechocystis sp. PCC 6803 Culture. *Journal of agricultural and food chemistry* **66**, 8522-8529, doi:10.1021/acs.jafc.8b01522 (2018).

59 Neale, P. A., Jämting, Å. K., O'Malley, E., Herrmann, J. & Escher, B. I. Behaviour of titanium dioxide and zinc oxide nanoparticles in the presence of wastewater-derived organic matter and implications for algal toxicity. *Environmental Science: Nano* **2**, 86-93, doi:10.1039/c4en00161c (2015).

60 Jeon, H.-S., Park, S. E., Ahn, B. & Kim, Y.-K. Enhancement of biodiesel production in Chlorella vulgaris cultivation using silica nanoparticles. *Biotechnology and Bioprocess Engineering* **22**, 136-141 (2017).

61 San, N. O. *et al.* Novel one-step synthesis of silica nanoparticles from sugarbeet bagasse by laser ablation and their effects on the growth of freshwater algae culture. *Particuology* **17**, 29-35, doi:10.1016/j.partic.2013.11.003 (2014).

62 Ren, H. Y. *et al.* Enhanced microalgal growth and lipid accumulation by addition of different nanoparticles under xenon lamp illumination. *Bioresour Technol* **297**, 122409, doi:10.1016/j.biortech.2019.122409 (2020).

63 Saxena, A., Prakash, K., Phogat, S., Singh, P. K. & Tiwari, A. Inductively coupled plasma nanosilica based growth method for enhanced biomass production in marine diatom algae. *Bioresource Technology* **314**, 123747 (2020).

64 Karunakaran, G., Suriyaprabha, R., Rajendran, V. & Kannan, N. Toxicity evaluation based on particle size, contact angle and zeta potential of SiO2 and Al2O3 on the growth of green algae. *Advances in nano research* **3**, 243 (2015).

65 Ahn, B., Park, S. E., Oh, B. K. & Kim, Y. K. Effect of nanoparticle on cellular growth and lipid production in Chlorella vulgaris culture. *Biotechnology progress* **34**, 929-933, doi:10.1002/btpr.2641 (2018).

66 Mahawar, H., Prasanna, R., Singh, S. B. & Nain, L. Influence of Silver, Zinc Oxide and Copper Oxide Nanoparticles on the Cyanobacterium Calothrix elenkinii. *BioNanoScience* **8**, 802-810, doi:10.1007/s12668-018-0543-2 (2018).

67 Pham, T.-L. Effect of Silver Nanoparticles on Tropical Freshwater and Marine Microalgae. *Journal of Chemistry* **2019**, 1-7, doi:10.1155/2019/9658386 (2019).

68 Sendra, M., Blasco, J. & Araújo, C. V. M. Is the cell wall of marine phytoplankton a protective barrier or a nanoparticle interaction site? Toxicological responses of Chlorella autotrophica and Dunaliella salina to Ag and CeO2 nanoparticles. *Ecological Indicators* **95**, 1053-1067, doi:10.1016/j.ecolind.2017.08.050 (2018).

69 Sendra, M., Yeste, P. M., Moreno-Garrido, I., Gatica, J. M. & Blasco, J. CeO2 NPs, toxic or protective to phytoplankton? Charge of nanoparticles and cell wall as factors which cause changes in cell complexity. *The Science of the total environment* **590-591**, 304-315, doi:10.1016/j.scitotenv.2017.03.007 (2017).

70 Zhang, P. *et al.* Distribution and bioavailability of ceria nanoparticles in an aquatic ecosystem model. *Chemosphere* **89**, 530-535, doi:10.1016/j.chemosphere.2012.05.044 (2012).

71 Mykhaylenko, N. F. & Zolotareva, E. K. The Effect of Copper and Selenium Nanocarboxylates on Biomass Accumulation and Photosynthetic Energy Transduction Efficiency of the Green Algae Chlorella Vulgaris. *Nanoscale Res Lett* **12**, 147, doi:10.1186/s11671-017-1914-2 (2017).

72 Castro-Bugallo, A., Gonzalez-Fernandez, A., Guisande, C. & Barreiro, A. Comparative responses to metal oxide nanoparticles in marine phytoplankton. *Archives of environmental contamination and toxicology* **67**, 483-493, doi:10.1007/s00244-014-0044-4 (2014).

73 Ji, J., Long, Z. & Lin, D. Toxicity of oxide nanoparticles to the green algae Chlorella sp. *Chemical Engineering Journal* **170**, 525-530, doi:10.1016/j.cej.2010.11.026 (2011).

74 Morelli, E., Cioni, P., Posarelli, M. & Gabellieri, E. Chemical stability of CdSe quantum dots in seawater and their effects on a marine microalga. *Aquat Toxicol* **122-123**, 153-162, doi:10.1016/j.aquatox.2012.06.012 (2012).

75 Morelli, E., Salvadori, E., Bizzarri, R., Cioni, P. & Gabellieri, E. Interaction of CdSe/ZnS quantum dots with the marine diatom Phaeodactylum tricornutum and the green alga Dunaliella tertiolecta: a biophysical approach. *Biophysical chemistry* **182**, 4-10, doi:10.1016/j.bpc.2013.06.007 (2013).

76 Rudic, V. *et al*. Red Algae <I>Porphyridium cruentum</I> Growth Stimulated by CdSe Quantum Dots Covered with Thioglycerol. *Journal of Nanoelectronics and Optoelectronics* **7**, 681-687, doi:10.1166/jno.2012.1416 (2012).

77 Conradt, R. Prospects and physical limits of processes and technologies in glass melting. *Journal of Asian Ceramic Societies* **7**, 377-396 (2019).

78 https://www.genlab.co.uk/25-high-temperature-ovens-500c.

79 https://www.retsch.com/.

80 Campion, N. Advancing Life Cycle Assessment: Perspectives from the Building and Healthcare Industries. (2015).

81 https://cms.esi.info/Media/documents/ColeP_mixers_ML.pdf.

82 https://www.fritsch-international.com/sample-preparation/applications-solutions/.

83 https://docs.rs-online.com/c9c5/0900766b81158007.pdf.

84 https://www.retsch.com/dltmp/www/55d3669d-2fa8-4491-8e80-41f7bc282b86-c77541695d35/brochure_catalogue_general_en.pdf.

85 Suryanarayana, C. Mechanical alloying and milling. *Progress in materials science* **46**, 1-184 (2001).

86 https://www.miw.co.uk/our-range/water-coolers/standard-floor-water-cooler-cold-ambient.

87 Koltun, P. & Tharumarajah, A. Life cycle impact of rare earth elements. *International Scholarly Research Notices* **2014** (2014).

88 Phenrat, T. *et al*. Stabilization of aqueous nanoscale zerovalent iron dispersions by anionic polyelectrolytes: adsorbed anionic polyelectrolyte layer properties and their effect on aggregation and sedimentation. *Journal of Nanoparticle Research* **10**, 795-814 (2008).

89 https://www.accela.eu/files/products/227/cr30nx_en.pdf.

90 USGS. Mineral Commodity Summaries. *US Geological Survey* (2020).

91 https://www.chemcentral.co.uk.

92 https://www.chinadaily.com.cn.

93 https://www.shopairproducts.co.uk/product.php?id=123532.

94 Bourgeault, A. *et al.* The challenge of studying TiO2 nanoparticle bioaccumulation at environmental concentrations: crucial use of a stable isotope tracer. *Environmental science & technology* **49**, 2451-2459 (2015).

95 Kwon, D., Jeon, S. K. & Yoon, T. H. Impact of agglomeration on the bioaccumulation of sub-100 nm sized TiO2. *Colloids and Surfaces B: Biointerfaces* **116**, 277-283 (2014).

96 Kwon, D., Nho, H. W. & Yoon, T. H. Transmission electron microscopy and scanning transmission X-ray microscopy studies on the bioaccumulation and tissue level absorption of TiO2 nanoparticles in Daphnia magna. *Journal of nanoscience and nanotechnology* **15**, 4229-4238 (2015).

97 Dalai, S. *et al.* Different modes of TiO2 uptake by Ceriodaphnia dubia: Relevance to toxicity and bioaccumulation. *Aquatic toxicology* **152**, 139-146 (2014).

98 Lovern, S. B. & Klaper, R. Daphnia magna mortality when exposed to titanium dioxide and fullerene (C60) nanoparticles. *Environmental Toxicology and Chemistry: An International Journal* **25**, 1132-1137 (2006).

99 Miller, R. J. *et al.* Impacts of metal oxide nanoparticles on marine phytoplankton. *Environmental science & technology* **44**, 7329-7334 (2010).

100 Verma, S. K. *et al.* Mechanistic insight into ROS and neutral lipid alteration induced toxicity in the human model with fins (Danio rerio) by industrially synthesized titanium dioxide nanoparticles. *Toxicology research* **7**, 244-257 (2018).

101 Hu, Q., Guo, F., Zhao, F. & Fu, Z. Effects of titanium dioxide nanoparticles exposure on parkinsonism in zebrafish larvae and PC12. *Chemosphere* **173**, 373-379, doi:10.1016/j.chemosphere.2017.01.063 (2017).

102 Kotil, T., Akbulut, C. & Yon, N. D. The effects of titanium dioxide nanoparticles on ultrastructure of zebrafish testis (Danio rerio). *Micron* **100**, 38-44, doi:10.1016/j.micron.2017.04.006 (2017).

103 Cherchi, C. & Gu, A. Z. Impact of titanium dioxide nanomaterials on nitrogen fixation rate and intracellular nitrogen storage in Anabaena variabilis. *Environmental science & technology* **44**, 8302-8307 (2010).

104 Galloway, T. *et al.* Sublethal toxicity of nano-titanium dioxide and carbon nanotubes in a sediment dwelling marine polychaete. *Environmental pollution* **158**, 1748-1755 (2010).

105 Canesi, L. *et al.* Biomarkers in Mytilus galloprovincialis exposed to suspensions of selected nanoparticles (Nano carbon black, C60 fullerene, Nano-TiO2, Nano-SiO2). *Aquat Toxicol* **100**, 168-177, doi:10.1016/j.aquatox.2010.04.009 (2010).

106 Zhu, X., Zhou, J. & Cai, Z. The toxicity and oxidative stress of TiO2 nanoparticles in marine abalone (Haliotis diversicolor supertexta). *Marine pollution bulletin* **63**, 334-338, doi:10.1016/j.marpolbul.2011.03.006 (2011).

107 Zhu, X., Zhou, J. & Cai, Z. TiO2 nanoparticles in the marine environment: impact on the toxicity of tributyltin to abalone (Haliotis diversicolor supertexta) embryos. *Environ Sci Technol* **45**, 3753-3758, doi:10.1021/es103779h (2011).

108 Cupi, D., Hartmann, N. B. & Baun, A. The influence of natural organic matter and aging on suspension stability in guideline toxicity testing of silver, zinc oxide, and titanium dioxide nanoparticles with Daphnia magna. *Environ Toxicol Chem* **34**, 497-506, doi:10.1002/etc.2855 (2015).

109 Lin, D., Ji, J., Long, Z., Yang, K. & Wu, F. The influence of dissolved and surface-bound humic acid on the toxicity of TiO(2) nanoparticles to Chlorella sp. *Water Res* **46**, 4477-4487, doi:10.1016/j.watres.2012.05.035 (2012).

110 Zhu, X., Chang, Y. & Chen, Y. Toxicity and bioaccumulation of TiO2 nanoparticle aggregates in Daphnia magna. *Chemosphere* **78**, 209-215, doi:10.1016/j.chemosphere.2009.11.013 (2010).

111 Wiench, K. *et al.* Acute and chronic effects of nano- and non-nano-scale TiO(2) and ZnO particles on mobility and reproduction of the freshwater invertebrate Daphnia magna. *Chemosphere* **76**, 1356-1365, doi:10.1016/j.chemosphere.2009.06.025 (2009).

112 Zhu, X., Zhu, L., Chen, Y. & Tian, S. Acute toxicities of six manufactured nanomaterial suspensions to Daphnia magna. *Journal of Nanoparticle Research* **11**, 67-75, doi:10.1007/s11051-008-9426-8 (2008).

113 Wang, J., Zhang, X., Chen, Y., Sommerfeld, M. & Hu, Q. Toxicity assessment of manufactured nanomaterials using the unicellular green alga Chlamydomonas reinhardtii. *Chemosphere* **73**, 1121-1128, doi:10.1016/j.chemosphere.2008.07.040 (2008).

114 Rekulapally, R., Chavali, L. N. M., Idris, M. M. & Singh, S. Toxicity of TiO2, SiO2, ZnO, CuO, Au and Ag engineered nanoparticles on hatching and early nauplii of Artemia sp. *PeerJ* **6**, e6138 (2019).

115 Schiavo, S., Oliviero, M., Miglietta, M., Rametta, G. & Manzo, S. Genotoxic and cytotoxic effects of ZnO nanoparticles for Dunaliella tertiolecta and comparison with SiO2 and TiO2 effects at population growth inhibition levels. *Science of the Total Environment* **550**, 619-627 (2016).

116 Manzo, S. *et al.* The diverse toxic effect of SiO 2 and TiO 2 nanoparticles toward the marine microalgae Dunaliella tertiolecta. *Environmental Science and Pollution Research* **22**, 15941-15951 (2015).

117 Dai, L., Syberg, K., Banta, G. T., Selck, H. & Forbes, V. E. Effects, Uptake, and Depuration Kinetics of Silver Oxide and Copper Oxide Nanoparticles in a Marine Deposit Feeder, Macoma balthica. *ACS Sustainable Chemistry & Engineering* **1**, 760-767, doi:10.1021/sc4000434 (2013).

118 Hanna, S. K., Miller, R. J., Zhou, D., Keller, A. A. & Lenihan, H. S. Accumulation and toxicity of metal oxide nanoparticles in a soft-sediment estuarine amphipod. *Aquatic toxicology* **142-143**, 441-446, doi:10.1016/j.aquatox.2013.09.019 (2013).

119 Xiao, Y., Peijnenburg, W. J., Chen, G. & Vijver, M. G. Toxicity of copper nanoparticles to Daphnia magna under different exposure conditions. *The Science of the total environment* **563-564**, 81-88, doi:10.1016/j.scitotenv.2016.04.104 (2016).

120 Croteau, M. N., Misra, S. K., Luoma, S. N. & Valsami-Jones, E. Bioaccumulation and toxicity of CuO nanoparticles by a freshwater invertebrate after waterborne and dietborne exposures. *Environ Sci Technol* **48**, 10929-10937, doi:10.1021/es5018703 (2014).

121 Pang, C. *et al.* Bioaccumulation, toxicokinetics, and effects of copper from sediment spiked with aqueous Cu, nano-CuO, or micro-CuO in the deposit-feeding snail, Potamopyrgus antipodarum. *Environ Toxicol Chem* **32**, 1561-1573, doi:10.1002/etc.2216 (2013).

122 Ramskov, T. *et al.* Bioaccumulation and effects of different-shaped copper oxide nanoparticles in the deposit-feeding snail Potamopyrgus antipodarum. *Environ Toxicol Chem* **33**, 1976-1987, doi:10.1002/etc.2639 (2014).

123 Ates, M., Arslan, Z., Demir, V., Daniels, J. & Farah, I. O. Accumulation and toxicity of CuO and ZnO nanoparticles through waterborne and dietary exposure of goldfish (Carassius auratus). *Environmental toxicology* **30**, 119-128, doi:10.1002/tox.22002 (2015).

124 Zhang, Y. *et al.* Transcriptional responses and mechanisms of copper nanoparticle toxicology on zebrafish embryos. *J Hazard Mater* **344**, 1057-1068, doi:10.1016/j.jhazmat.2017.11.039 (2018).

125 Thit, A., Skjolding, L. M., Selck, H. & Sturve, J. Effects of copper oxide nanoparticles and copper ions to zebrafish (Danio rerio) cells, embryos and fry. *Toxicology in vitro : an international journal published in association with BIBRA* **45**, 89-100, doi:10.1016/j.tiv.2017.08.010 (2017).

126 Xu, J. *et al.* Copper impairs zebrafish swimbladder development by down-regulating Wnt signaling. *Aquat Toxicol* **192**, 155-164, doi:10.1016/j.aquatox.2017.09.018 (2017).

127 Hu, W. *et al.* Toxicity of copper oxide nanoparticles in the blue mussel, Mytilus edulis: a redox proteomic investigation. *Chemosphere* **108**, 289-299, doi:10.1016/j.chemosphere.2014.01.054 (2014).

128 Buffet, P. E. *et al.* Behavioural and biochemical responses of two marine invertebrates Scrobicularia plana and Hediste diversicolor to copper oxide nanoparticles. *Chemosphere* **84**, 166-174, doi:10.1016/j.chemosphere.2011.02.003 (2011).

129 Heinlaan, M., Ivask, A., Blinova, I., Dubourguier, H. C. & Kahru, A. Toxicity of nanosized and bulk ZnO, CuO and TiO2 to bacteria Vibrio fischeri and crustaceans Daphnia magna and Thamnocephalus platyurus. *Chemosphere* **71**, 1308-1316, doi:10.1016/j.chemosphere.2007.11.047 (2008).

130 Blinova, I., Ivask, A., Heinlaan, M., Mortimer, M. & Kahru, A. Ecotoxicity of nanoparticles of CuO and ZnO in natural water. *Environ Pollut* **158**, 41-47, doi:10.1016/j.envpol.2009.08.017 (2010).

131 Xiao, Y., Vijver, M. G., Chen, G. & Peijnenburg, W. J. Toxicity and accumulation of Cu and ZnO nanoparticles in Daphnia magna. *Environ Sci Technol* **49**, 4657-4664, doi:10.1021/acs.est.5b00538 (2015).

132 Garaud, M. *et al.* Multibiomarker assessment of cerium dioxide nanoparticle (nCeO2) sublethal effects on two freshwater invertebrates, Dreissena polymorpha and Gammarus roeseli. *Aquat Toxicol* **158**, 63-74, doi:10.1016/j.aquatox.2014.11.004 (2015).

133 Gambardella, C. *et al.* Effects of selected metal oxide nanoparticles on Artemia salina larvae: evaluation of mortality and behavioural and biochemical responses. *Environmental monitoring and assessment* **186**, 4249-4259, doi:10.1007/s10661-014-3695-8 (2014).

134 Falugi, C. *et al.* Toxicity of metal oxide nanoparticles in immune cells of the sea urchin. *Marine environmental research* **76**, 114-121, doi:10.1016/j.marenvres.2011.10.003 (2012).

135 Montes, M. O., Hanna, S. K., Lenihan, H. S. & Keller, A. A. Uptake, accumulation, and biotransformation of metal oxide nanoparticles by a marine suspension-feeder. *Journal of hazardous materials* **225-226**, 139-145, doi:10.1016/j.jhazmat.2012.05.009 (2012).

136 Auffan, M. *et al.* Role of molting on the biodistribution of CeO2 nanoparticles within Daphnia pulex. *Water Res* **47**, 3921-3930, doi:10.1016/j.watres.2012.11.063 (2013).

137 Artells, E. *et al.* Exposure to cerium dioxide nanoparticles differently affect swimming performance and survival in two daphnid species. *PLoS One* **8**, e71260, doi:10.1371/journal.pone.0071260 (2013).

138 Manier, N., Bado-Nilles, A., Delalain, P., Aguerre-Chariol, O. & Pandard, P. Ecotoxicity of non-aged and aged CeO2 nanomaterials towards freshwater microalgae. *Environ Pollut* **180**, 63-70, doi:10.1016/j.envpol.2013.04.040 (2013).

139 Van Hoecke, K., De Schamphelaere, K. A., Van der Meeren, P., Smagghe, G. & Janssen, C. R. Aggregation and ecotoxicity of CeO(2) nanoparticles in synthetic and natural waters with variable pH, organic matter concentration and ionic strength. *Environ Pollut* **159**, 970-976, doi:10.1016/j.envpol.2010.12.010 (2011).

140 Skjolding, L. M. *et al.* Uptake and depuration of gold nanoparticles in Daphnia magna. *Ecotoxicology* **23**, 1172-1183, doi:10.1007/s10646-014-1259-x (2014).

141 Khan, F. R. *et al.* Stable isotope tracer to determine uptake and efflux dynamics of ZnO Nano- and bulk particles and dissolved Zn to an estuarine snail. *Environ Sci Technol* **47**, 8532-8539, doi:10.1021/es4011465 (2013).

142 Skjolding, L. M., Winther-Nielsen, M. & Baun, A. Trophic transfer of differently functionalized zinc oxide nanoparticles from crustaceans (Daphnia magna) to zebrafish (Danio rerio). *Aquat Toxicol* **157**, 101-108, doi:10.1016/j.aquatox.2014.10.005 (2014).

143 Hao, L., Chen, L., Hao, J. & Zhong, N. Bioaccumulation and sub-acute toxicity of zinc oxide nanoparticles in juvenile carp (Cyprinus carpio): a comparative study with its bulk counterparts. *Ecotoxicology and environmental safety* **91**, 52-60 (2013).

144 Kim, B. *et al.* Magnesium aminoclay enhances lipid production of mixotrophic Chlorella sp. KR-1 while reducing bacterial populations. *Bioresour Technol* **219**, 608-613, doi:10.1016/j.biortech.2016.08.034 (2016).

145 Kteeba, S. M. *et al.* Exposure to ZnO nanoparticles alters neuronal and vascular development in zebrafish: Acute and transgenerational effects mitigated with dissolved organic matter. *Environ Pollut* **242**, 433-448, doi:10.1016/j.envpol.2018.06.030 (2018).

146 Peng, X., Palma, S., Fisher, N. S. & Wong, S. S. Effect of morphology of ZnO nanostructures on their toxicity to marine algae. *Aquatic Toxicology* **102**, 186-196 (2011).

147 Manzo, S., Miglietta, M. L., Rametta, G., Buono, S. & Di Francia, G. Toxic effects of ZnO nanoparticles towards marine algae Dunaliella tertiolecta. *The Science of the total environment* **445-446**, 371-376, doi:10.1016/j.scitotenv.2012.12.051 (2013).

148 Zhang, H., Huang, Q., Xu, A. & Wu, L. Spectroscopic probe to contribution of physicochemical transformations in the toxicity of aged ZnO NPs to Chlorella vulgaris: new insight into the variation of toxicity of ZnO NPs under aging process. *Nanotoxicology* **10**, 1177-1187, doi:10.1080/17435390.2016.1196252 (2016).

149 Tang, Y., Li, S., Lu, Y., Li, Q. & Yu, S. The influence of humic acid on the toxicity of nano-ZnO and Zn2+ to the Anabaena sp. *Environmental toxicology* **30**, 895-903, doi:10.1002/tox.21964 (2015).

150 Wong, S. W., Leung, P. T., Djurisic, A. B. & Leung, K. M. Toxicities of nano zinc oxide to five marine organisms: influences of aggregate size and ion solubility. *Analytical and bioanalytical chemistry* **396**, 609-618, doi:10.1007/s00216-009-3249-z (2010).

151 Fabrega, J. *et al.* Sequestration of zinc from zinc oxide nanoparticles and life cycle effects in the sediment dweller amphipod Corophium volutator. *Environ Sci Technol* **46**, 1128-1135, doi:10.1021/es202570g (2012).

152 Benavides, M., Fernandez-Lodeiro, J., Coelho, P., Lodeiro, C. & Diniz, M. S. Single and combined effects of aluminum (Al2O3) and zinc (ZnO) oxide nanoparticles in a freshwater fish, Carassius auratus. *Environmental science and pollution research international* **23**, 24578-24591, doi:10.1007/s11356-016-7915-3 (2016).

153 Katsumiti, A. *et al.* Cytotoxicity of Au, ZnO and SiO2 NPs using in vitro assays with mussel hemocytes and gill cells: relevance of size, shape and additives. *Nanotoxicology* **10**, 185-193 (2016).

154 Lacave, J. M. *et al.* Effects of metal-bearing nanoparticles (Ag, Au, CdS, ZnO, SiO2) on developing zebrafish embryos. *Nanotechnology* **27**, 325102 (2016).

155 Pakrashi, S., Dalai, S., Chandrasekaran, N. & Mukherjee, A. Trophic transfer potential of aluminium oxide nanoparticles using representative primary producer (Chlorella ellipsoides) and a primary consumer (Ceriodaphnia dubia). *Aquatic toxicology* **152**, 74-81, doi:10.1016/j.aquatox.2014.03.024 (2014).

156 Ates, M. *et al.* Evaluation of alpha and gamma aluminum oxide nanoparticle accumulation, toxicity, and depuration in Artemia salina larvae. *Environmental toxicology* **30**, 109-118 (2015).

157 Zhang, Y., Zhu, L., Zhou, Y. & Chen, J. Accumulation and elimination of iron oxide nanomaterials in zebrafish (Danio rerio) upon chronic aqueous exposure. *Journal of environmental sciences* **30**, 223-230, doi:10.1016/j.jes.2014.08.024 (2015).

158 Ates, M. *et al.* Chronic exposure of tilapia (Oreochromis niloticus) to iron oxide nanoparticles: Effects of particle morphology on accumulation, elimination, hematology and immune responses. *Aquat Toxicol* **177**, 22-32, doi:10.1016/j.aquatox.2016.05.005 (2016).

159 Zhu, X., Tian, S. & Cai, Z. Toxicity assessment of iron oxide nanoparticles in zebrafish (Danio rerio) early life stages. *PLoS One* **7**, e46286, doi:10.1371/journal.pone.0046286 (2012).

160 Remya, A. S. *et al.* Iron oxide nanoparticles to an Indian major carp, Labeo rohita: Impacts on hematology, iono regulation and gill Na+/K+ ATPase activity. *Journal of King Saud University - Science* **27**, 151-160, doi:10.1016/j.jksus.2014.11.002 (2015).

161 Souza, L. R. R., Bernardes, L. E., Barbetta, M. F. S. & da Veiga, M. Iron oxide nanoparticle phytotoxicity to the aquatic plant Lemna minor: effect on reactive oxygen species (ROS) production and chlorophyll a/chlorophyll b ratio. *Environmental science and pollution research international* **26**, 24121-24131, doi:10.1007/s11356-019-05713-x (2019).

162 Magro, M., De Liguoro, M., Franzago, E., Baratella, D. & Vianello, F. The surface reactivity of iron oxide nanoparticles as a potential hazard for aquatic environments: A study on Daphnia magna adults and embryos. *Sci Rep* **8**, 13017, doi:10.1038/s41598-018-31483-6 (2018).

163 Lei, C., Zhang, L., Yang, K., Zhu, L. & Lin, D. Toxicity of iron-based nanoparticles to green algae: Effects of particle size, crystal phase, oxidation state and environmental aging. *Environ Pollut* **218**, 505-512, doi:10.1016/j.envpol.2016.07.030 (2016).

164 Ozgur, M. E. *et al.* The Toxicity Assessment of Iron Oxide (Fe(3)O(4)) Nanoparticles on Physical and Biochemical Quality of Rainbow Trout Spermatozoon. *Toxics* **6**, doi:10.3390/toxics6040062 (2018).

165 Mashjoor, S., Yousefzadi, M., Zolgharnain, H., Kamrani, E. & Alishahi, M. Organic and inorganic nano-Fe3O4: Alga Ulva flexuosa-based synthesis, antimicrobial effects and acute toxicity to briny water rotifer Brachionus rotundiformis. *Environ Pollut* **237**, 50-64, doi:10.1016/j.envpol.2018.02.036 (2018).

166 Zhu, S. *et al.* Developmental toxicity of Fe3O4 nanoparticles on cysts and three larval stages of Artemia salina. *Environ Pollut* **230**, 683-691, doi:10.1016/j.envpol.2017.06.065 (2017).

167 Zheng, M., Lu, J. & Zhao, D. Effects of starch-coating of magnetite nanoparticles on cellular uptake, toxicity and gene expression profiles in adult zebrafish. *The Science of the total environment* **622-623**, 930-941, doi:10.1016/j.scitotenv.2017.12.018 (2018).

168 Shariati, F., Poordeljoo, T. & Zanjanchi, P. The Acute Toxicity of SiO 2 and Fe 3 O 4 Nano-particles on Daphnia magna. *Silicon*, 1-6 (2020).

169 Kadar, E., Tarran, G. A., Jha, A. N. & Al-Subiai, S. N. Stabilization of engineered zero-valent nanoiron with Na-acrylic copolymer enhances spermiotoxicity. *Environ Sci Technol* **45**, 3245-3251, doi:10.1021/es1029848 (2011).

170 Keller, A. A., Garner, K., Miller, R. J. & Lenihan, H. S. Toxicity of nano-zero valent iron to freshwater and marine organisms. *PloS one* **7** (2012).

171 Canesi, L. *et al.* In vitro effects of suspensions of selected nanoparticles (C60 fullerene, TiO2, SiO2) on Mytilus hemocytes. *Aquat Toxicol* **96**, 151-158, doi:10.1016/j.aquatox.2009.10.017 (2010).

172 Koehler, A., Marx, U., Broeg, K., Bahns, S. & Bressling, J. Effects of nanoparticles in Mytilus edulis gills and hepatopancreas–a new threat to marine life? *Marine environmental research* **66**, 12-14 (2008).

173 Van Hoecke, K. *et al.* Influence of alumina coating on characteristics and effects of SiO2 nanoparticles in algal growth inhibition assays at various pH and organic matter contents. *Environment international* **37**, 1118-1125 (2011).

174 Puerari, R. C. *et al.* Acute and chronic toxicity of amine-functionalized SiO2 nanostructures toward Daphnia magna. *Ecotoxicology and Environmental Safety* **212**, 111979 (2021).

175 Yang, S., Ye, R., Han, B., Wei, C. & Yang, X. Ecotoxicological effect of nano-silicon dioxide particles on Daphnia magna. *Integrated Ferroelectrics* **154**, 64-72 (2014).

176 Ayatallahzadeh Shirazi, M., Shariati, F. & Ramezanpour, Z. Toxicity effects of SiO2 nanoparticles on green micro-algae Dunaliella salina. *International Journal of Nanoscience and Nanotechnology* **12**, 269-275 (2016).

177 Wei, C. *et al.* Effects of silica nanoparticles on growth and photosynthetic pigment contents of Scenedesmus obliquus. *Journal of environmental sciences* **22**, 155-160 (2010).

178 Srikanth, K., Trindade, T., Duarte, A. C. & Pereira, E. Cytotoxicity and oxidative stress responses of silica-coated iron oxide nanoparticles in CHSE-214 cells. *Environmental science and pollution research international* **24**, 2055-2064, doi:10.1007/s11356-016-7870-z (2017).

179 Khan, F. R. *et al.* Dietary bioavailability of cadmium presented to the gastropod Peringia ulvae as quantum dots and in ionic form. *Environ Toxicol Chem* **32**, 2621-2629, doi:10.1002/etc.2348 (2013).

180 Bouldin, J. L. *et al.* Aqueous toxicity and food chain transfer of quantum dots™ in freshwater algae and Ceriodaphnia dubia. *Environmental Toxicology and Chemistry: An International Journal* **27**, 1958-1963 (2008).

181 Jackson, B. P., Bugge, D., Ranville, J. F. & Chen, C. Y. Bioavailability, toxicity, and bioaccumulation of quantum dot nanoparticles to the amphipod Leptocheirus plumulosus. *Environ Sci Technol* **46**, 5550-5556, doi:10.1021/es202864r (2012).

182 Kalinowska, D., Grabowska-Jadach, I., Drozd, M. & Pietrzak, M. Comparative studies of biological activity of cadmium-based quantum dots with different surface modifications. *Applied Nanoscience* **8**, 309-321, doi:10.1007/s13204-018-0787-8 (2018).

183 King-Heiden, T. C. *et al.* Quantum dot nanotoxicity assessment using the zebrafish embryo. *Environmental science & technology* **43**, 1605-1611 (2009).

184 Lewinski, N. A. *et al.* Quantification of water solubilized CdSe/ZnS quantum dots in Daphnia magna. *Environmental science & technology* **44**, 1841-1846 (2010).

185 Yao, K. *et al.* Effects of Carbon Quantum Dots on Aquatic Environments: Comparison of Toxicity to Organisms at Different Trophic Levels. *Environ Sci Technol* **52**, 14445-14451, doi:10.1021/acs.est.8b04235 (2018).

186 Zarco-Fernandez, S. *et al.* Bioconcentration of ionic cadmium and cadmium selenide quantum dots in zebrafish larvae. *Chemosphere* **148**, 328-335, doi:10.1016/j.chemosphere.2015.12.077 (2016).

187 Pace, H. E., Lesher, E. K. & Ranville, J. F. Influence of stability on the acute toxicity of CdSe/ZnS nanocrystals to Daphnia magna. *Environ Toxicol Chem* **29**, 1338-1344, doi:10.1002/etc.168 (2010).

188 Zhao, X. *et al.* Evaluating the toxicity of hydroxyapatite nanoparticles in catfish cells and zebrafish embryos. *Small* **9**, 1734-1741, doi:10.1002/smll.201200639 (2013).
